# Bacterial population and biodegradation potential in chronically crude oil-contaminated marine sediments are strongly linked to temperature

**DOI:** 10.1038/srep11651

**Published:** 2015-06-29

**Authors:** Rafael Bargiela, Francesca Mapelli, David Rojo, Bessem Chouaia, Jesús Tornés, Sara Borin, Michael Richter, Mercedes V. Del Pozo, Simone Cappello, Christoph Gertler, María Genovese, Renata Denaro, Mónica Martínez-Martínez, Stilianos Fodelianakis, Ranya A. Amer, David Bigazzi, Xifang Han, Jianwei Chen, Tatyana N. Chernikova, Olga V. Golyshina, Mouna Mahjoubi, Atef Jaouanil, Fatima Benzha, Mirko Magagnini, Emad Hussein, Fuad Al-Horani, Ameur Cherif, Mohamed Blaghen, Yasser R. Abdel-Fattah, Nicolas Kalogerakis, Coral Barbas, Hanan I. Malkawi, Peter N. Golyshin, Michail M. Yakimov, Daniele Daffonchio, Manuel Ferrer

**Affiliations:** 1 Institute of Catalysis, Consejo Superior de Investigaciones Científicas, Madrid, Spain; 2 Department of Food, Environmental and Nutritional Sciences (DeFENS), University of Milan, Milan, Italy; 3 Centro de Metabolómica y Bioanálisis (CEMBIO), Facultad de Farmacia, Universidad CEU San Pablo, Campus Montepríncipe, Madrid, Spain; 4 Ribocon GmbH, Bremen, Germany; 5 Institute for Coastal Marine Environment, Consiglio Nazionale delle Ricerche, Messina, Italy; 6 School of Biological Sciences, Bangor University, Bangor, UK; 7 School of Environmental Engineering, TU-Crete, Chania, Greece; 8 Environmental Biotechnology Department, Genetic Engineering and Biotechnology Research Institute, City for Scientific Research & Technology Applications, Alexandria, Egypt; 9 EcoTechSystems Ltd., Ancona, Italy; 10 BGI Tech Solutions Co., Ltd, Main Building, Beishan Industrial Zone, Yantian District, Shenzhen, China; 11 LR Biotechnology and Bio-Geo Resources Valorization (LR11ES31), Higher Institute for Biotechnology - University of Manouba, Biotechpole of Sidi Thabet, 2020, Sidi Thabet, Ariana, Tunisia; 12 Laboratory of Microorganisms and Active Biomolecules, University of Tunis El Manar, Tunis, Tunisia; 13 Laboratory of Microbiology, Biotechnology and Environment, University Hassan II – Ain Chock, Casablanca, Morocco; 14 Department of Biological Sciences, Yarmouk University, Irbid, Jordan; 15 Faculty of Marine Sciences, The University of Jordan-Aqaba, Jordan; 16 Bioprocess Development Department, Genetic Engineering and Biotechnology Research Institute, City for Scientific Research & Technology Applications, Alexandria, Egypt; 17 Hamdan Bin Mohammad Smart University, Academic City, Dubai, United Arab Emirates; 18 King Abdullah University of Science and Technology, BESE Division, Thuwal, 23955-6900, Kingdom of Saudi Arabia

## Abstract

Two of the largest crude oil-polluted areas in the world are the semi-enclosed Mediterranean and Red Seas, but the effect of chronic pollution remains incompletely understood on a large scale. We compared the influence of environmental and geographical constraints and anthropogenic forces (hydrocarbon input) on bacterial communities in eight geographically separated oil-polluted sites along the coastlines of the Mediterranean and Red Seas. The differences in community compositions and their biodegradation potential were primarily associated (P < 0.05) with both temperature and chemical diversity. Furthermore, we observed a link between temperature and chemical and biological diversity that was stronger in chronically polluted sites than in pristine ones where accidental oil spills occurred. We propose that low temperature increases bacterial richness while decreasing catabolic diversity and that chronic pollution promotes catabolic diversification. Our results further suggest that the bacterial populations in chronically polluted sites may respond more promptly in degrading petroleum after accidental oil spills.

The chemical diversity of crude oil components and environmental constraints such as depth, O_2_ concentration, temperature, and nutrient input strongly influence microbial populations and the biodegradation processes they mediate in response to accidental oil spills in seawater and sediments^1^,^2^,^3^,^4^. In particular, the relative abundance of ubiquitous yet specialized hydrocarbonoclastic bacteria (HCB) of genera *Alcanivorax*, *Marinobacter*, *Oleispira*, *Thalassolituus*, *Oleiphilus*, *Cycloclasticus*, and *Neptunomonas*^5^,^6^, as well as the expression of catabolic genes involved in oil component degradation and genes relevant to carbon, nitrogen, phosphorous, sulfur, and iron cycling, are modulated by variations in crude oil input in seawater and marine sediments^1^,^2^,^3^,^4^,^7^,^8^,^9^,^10^,^11^,^12^.

Compared to sites of accidental crude oil spills, such as the Gulf of Mexico^13^ or the NW coast of Spain^14^, the Mediterranean Sea, particularly the southern part of the basin, has been neglected in studies of marine oil pollution, even though this region hosts large numbers of pipeline terminals, oil refineries, offshore platforms and 20% of global crude oil traffic, with consequent bunker accidents^15^,^16^,^17^,^18^. Notably, this region represents just 1% of the total marine surface of the planet. Several studies and reports have also demonstrated that numerous areas in the Mediterranean Sea are also polluted with toxic compounds other than crude oil components (UNEP/WHO, 1999), leading to a synergistic increase in overall toxicity^19^,^20^. Additionally, in contrast to open-sea areas, the Mediterranean Sea is a semi-enclosed basin with a water turnover time of 70–90 years, with a consequent rapid accumulation of toxic chemical species. This chronic crude oil input likely induces variation in bacterial populations; however the effects of parameters such as water temperature, O_2_ concentration and crude oil input on the distribution and degradation potential of these populations are unknown.

The release of thousands of tons of petroleum hydrocarbons (PHs) originating from anthropogenic activity affects the marine environment and causes severe ecological and economical damage. Once released at the sea surface, PHs undergo both weathering processes (evaporation of volatile fraction, photo-oxidation) and emulsification^12^,^15^; as a result, significant amount of PHs become heavier, form tar and settle on sediments^21^. Marine sediments are often fine-grained, and the abundance of clay minerals coupled with high organic loads encourages sorption of the most hydrophobic PHs. Sedimentary accretion can result in the burial of hydrocarbons in zones of low redox potential. As a consequence, hydrocarbons have been found in fine-grained coastal sediments many decades after a spill due to slow anaerobic biodegradation. Indeed, the analysis of organic contamination in superficial sediments performed in this study revealed high concentrations of different classes of hydrocarbon pollutants originating from human activities. For all these reasons, sediment samples were the focus of the present investigation.

This study is the first to report basin-wide trends of marine bacterial diversity, ecology, and biodegradation potential (using a meta-network approach) in seven major chronically oil-polluted and geographically separated sites along a latitude gradient across the Mediterranean Sea. The chosen Mediterranean regions are characterized by heavy industrialization and dense urbanization with large tanker traffic transporting crude and refined oil to and from the refineries located at these sites^15^,^16^,^17^,^18^,^19^,^20^. We hypothesized that temperature can drive major measurable catabolic changes in the resident microbial communities. For this reason, an oil terminal site in the Gulf of Aqaba in the Red Sea, an area subject to intense maritime traffic and chronic pollution and of particular relevance in light of global warming predictions^22^, was also included for comparative purposes. It is noteworthy that the Red Sea represents the potential future of the Mediterranean Sea as a result of its high seawater temperature^22^. By implementing an integrative metagenomic and metabolomics approach, our study highlights major changes at the level of bacterial components at colder and warmer sites in the Mediterranean Sea basin and the Red Sea. Thus, a positive impact of lower temperature on the total bacterial population was found, with a marked negative effect on catabolic diversity. A comparative analysis with pollutant-degrading webs established after the Horizon Oil Spill in the Gulf of Mexico provides further evidence suggesting that chronic pollution favors catabolic diversification.

## Results

### Study sites

The oil-polluted sites were located along the northern and southern sides of the Mediterranean Sea and the northern Red Sea^16^ and included, in order of latitude coordinates, the following (the site code is in parentheses): 1) the site of the 1991 Haven tanker shipwreck (HAV) in the northernmost part of the Ligurian Sea, Genoa, Italy; 2) the harbor of Messina (MES), Sicily, Italy; 3) the coast adjacent to an oil refinery unit in the Elefsina Bay (ELF), northwest of Athens, Greece; 4) the harbor of Priolo (PRI) Gargallo, Syracuse, Italy; 5) the Bizerte lagoon (BIZ), located in Northern Tunisia; 6) the lagoon of Mar Chica (MCh), located on the north-west Mediterranean coast of Morocco; 7) the El-Max site (ELMAX), located on the coast west of Alexandria, Egypt; and 8) the Gulf of Aqaba (AQ), along the Jordanian coast at the northern end of the Red Sea. Information on the sites is provided in the Supplementary Methods.

The eight studied sediment sites exhibited the following characteristics (average values) at the time of sampling (Supplementary Table S1): *i*) a temperature ranging from 13.0 (for ELF) to 26.5 °C (for AQ); *ii*) a pH of 6.85 (for PRI) to 8.62 (for MCh); *iii*) an oxygen concentration (measured at the water level immediately above the sediment) ranging from anoxic (for PRI) to micro-aerobic (for MES: 1.0–2.2 mg/L) to aerobic (maximum 22.0 mg/L for MCh); *iv*) a conductivity ranging from 13.1 (for BIZ) to 89.0 (for AQ) ms/cm; *v*) a total concentration of 116 (for BIZ) to 260,000 (for HAV) mg petroleum hydrocarbons/kg sediment; *vi*) a total NH_4_^+^ concentration of 0.65 (for BIZ) to 61 μmol/L (for MCh); *vii*) a total Ca^2+^ concentration of 35.8 (for BIZ) to 430 mg/L (for MCh); *viii*) a dissolved organic carbon (DOC) from 5.0 (for HAV) to 143 mg/L (for ELF); *ix*) a particulate organic carbon (POC) from 1.20 (for ELF) to 2.29 μM (for AQ); *x*) a total concentration of microelements of 67.3 (for MCh) to 411 nM (for ELMAX); and *xi*) cell counts ranging from ~1.90e^+09^ ± 1.15e^+09^ (for HAV) to ~2.22e^+08^ ± 1.41e^+08^ (for MES) cell/g sediment. The warmest area was located, as expected, in the Gulf of Aqaba.

The rationale behind the sampling strategy was to target sites with aged and chronic contamination and in diverse environmental locations in the Mediterranean basin and in the North Red Sea. Accordingly, the samples investigated were inevitably rather heterogeneous (e.g., distinct O_2_, conductivity, and NH_4_^+^, Ca^2+^, DOC, POC, microelements and hydrocarbon inputs). Nonetheless, the samples are representative of some of the most prevalent types of chronically polluted sites distributed within the highly diverse Mediterranean Sea and Red Sea^16^. Moreover, they constitute the basis of proof-of-concept for an integrated, multi-omics approach (metagenome- and metabolome-wide scan), contributing to unraveling the core environmental parameters that regulate microbial population and catabolic activities in chronically polluted marine sites.

### Bacterial populations can be categorized based on site temperature

The polluted sediments were first investigated in terms of the taxonomic diversity and composition of the resident bacterial communities using bar-coding of PCR-amplified bacterial 16S rRNA gene fragments obtained by 454 pyro-sequencing. The number of final reads and the number of Operational Taxonomic Units at 97% similarity (OTU_97_) varied among the samples, with good coverage of the bacterial community diversity in all cases (Supplementary Table S2). The sequencing results demonstrated that each of the sediments hosts a distinct bacterial community (Supplementary Fig. S1), with richness shifting from low Shannon index values, as in the case of samples AQ (3.18) and MCh (4.07), to high Shannon index values, such as those recorded for samples ELF (8.10), PRI (7.39), and HAV (7.32) (Supplementary Table S2).

**Figure 1:**
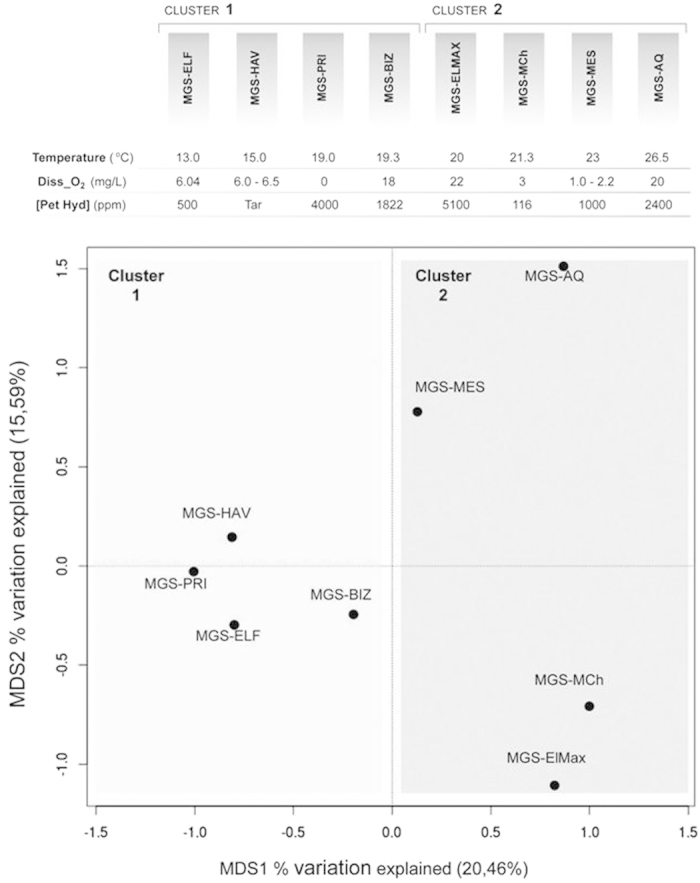
Principal coordinates analysis (PCoA) clustering Mediterranean Sea and Aqaba Gulf (Red Sea) polluted sediments according to the OTU_97_ composition generated from 16S rRNA gene pyrosequencing results. According to the sample distribution along MDS1, two clusters were identified through *t*-tests (*P* = 0.0017). The two clusters are indicated in the figure by light and dark grey boxes.

None of the total 18,435 OTU_97_ identified, which were distributed among 72 taxonomic groups across all phyla (Supplementary Table S3), was common to all the communities examined, suggesting a high metabolic heterogeneity among the clusters of samples (see Supplementary Results and Discussion). To explore bacterial community shifts between the different sediments, principal coordinates analysis was applied to depict similarity according to the OTU_97_ composition of the associated bacterial communities. Based on the sediment distribution in MDS1 (Fig. 1), which explains 20.46% of the total variation observed within the bacterial communities, the samples can be divided into two clusters (*t*-test, *P* = 0.0017): sediments collected at sites HAV, PRI, BIZ, and ELF formed Cluster 1, whereas those collected at sites AQ, MES, MCh, and ELMAX formed Cluster 2. The latter set of samples is characterized by a higher seawater temperature (from 20.0 to 26.5 °C) compared to the samples of Cluster 1 (from 13.0 to 19.3 °C). Moreover, two distinct subgroups are visible within Cluster 2: one corresponding to samples with the highest temperatures (MES [23.0 °C] and AQ [26.5 °C]) and another corresponding to samples with the lowest temperatures (MCh [21.3 °C] and ELMAX [20.0 °C]). No other environmental parameters among those examined herein explained these separations at the level of global species distribution. Taxonomic groups associated with the distinct clusters are discussed in Supplementary Results and Discussion. Taken together, we speculate that site temperature (Fig. 1) is a crucial driving factor controlling the overall species distribution in the chronically polluted sites investigated in this study in comparison to other environmental constraints such as geography, O_2_ concentration and crude oil inputs. Note, however, that these factors, alone or in combination with temperature, are also important secondary factors that influence the distribution of particular sets of bacterial groups (Supplementary Table S3 and Results and Discussion).

To evaluate whether community shifts might facilitate the establishment of a basin-scale distribution of catabolic capacities, particularly biodegradation capacities, we employed the recently developed PiCRUST analysis^23^. However, this technique, which is proposed to derive functional information from taxonomic data, failed to identify such differences at the investigated sites (Supplementary Table S4). Considering that one crucial goal of this research was to better understand the differential potential of microbial communities to degrade crude oil contaminants in chronically crude oil-contaminated marine sediments, we explored a second approach based on AromaDeg analysis^24^. AromaDeg is a web-based resource with an up-to-date and manually curated database with an associated query system that exploits phylogenomic analysis of the degradation of aromatic compounds. This database addresses systematic errors produced by standard methods of protein function prediction, such as PiCRUST, by improving the accuracy of functional classification of key genes, particularly those encoding proteins of aromatic degradation. In brief, each query sequence from a genome or metagenome that matches a given protein family of AromaDeg is associated with a key catabolic enzyme for an aromatic degradation reaction^24^,^25^,^26^. Individual reactions, and thus the corresponding substrate pollutants and intermediate degradation products (see Supplementary Table S5A), can be manually linked to reconstruct catabolic networks, as has been successfully reported for microbial communities from polluted soils^27^. As the number of samples and degradation reactions examined in this study was high, we decided to design an in-house script that allowed the automatic reconstruction of such networks in a graphical format. The script allowed visualization and comparison of the relative abundance (rel. ab.) of genes encoding catabolic enzymes (to avoid artifacts due to differences in sample size) assigned to distinct degradation reactions and the substrate pollutants or intermediates possibly degraded by each of the communities. The complete workflow, including the scripts and commands used for catabolic network reconstruction, is described in the Supplementary Methods, and the results are presented below.

### Reconstruction of catabolic networks: *in silico* prediction and experimental validations

Based on metagenomics data sets (meta-sequences), we identified a total of 238,449 potential protein-coding genes (≥20 amino acids long) (Supplementary Table S6). Among them, 2,011 (or 0.84% of the total) are genes encoding catabolic enzymes with matches in AromaDeg^24^,^27^. The rel. ab. of catabolic genes assigned to presumptive degradation reactions and the substrate pollutants or intermediates possibly degraded by each of the communities are shown in Supplementary Fig. S2 and Table S5B.

Due to the limited sequence coverage (≤2.9 Gbp of meta-sequences per sample), the reconstructed pathways were incomplete, as has been reported recently^27^. Thus, we refined the search for enzyme-encoding genes to fill the network gaps by examining a set of related genome sequence annotations established on the basis of 16S rRNA phylogenetic affiliations for each sample, as with PiCRUST analysis^23^. Of 610,277 potential protein-coding genes associated with OTU_97_ assignations, 13,440 were selected as matching AromaDeg^24^; these genes are presumptively involved in pollutant catabolism (Supplementary Fig. S3 and Table S5B).

As expected, the number of substrate pollutants or intermediates predicted as being potentially degraded on the basis of DNA and 16S rRNA data sets differed largely for those samples with the lowest DNA sequence coverage, namely HAV (DNA: 14; 16S rRNA: 40) and PRI (DNA: 3; 16S rRNA: 38); only minor differences (from 3 to 6 pollutants) were observed for the other samples for which high coverage was obtained (Supplementary Table S5B).

**Figure 2:**
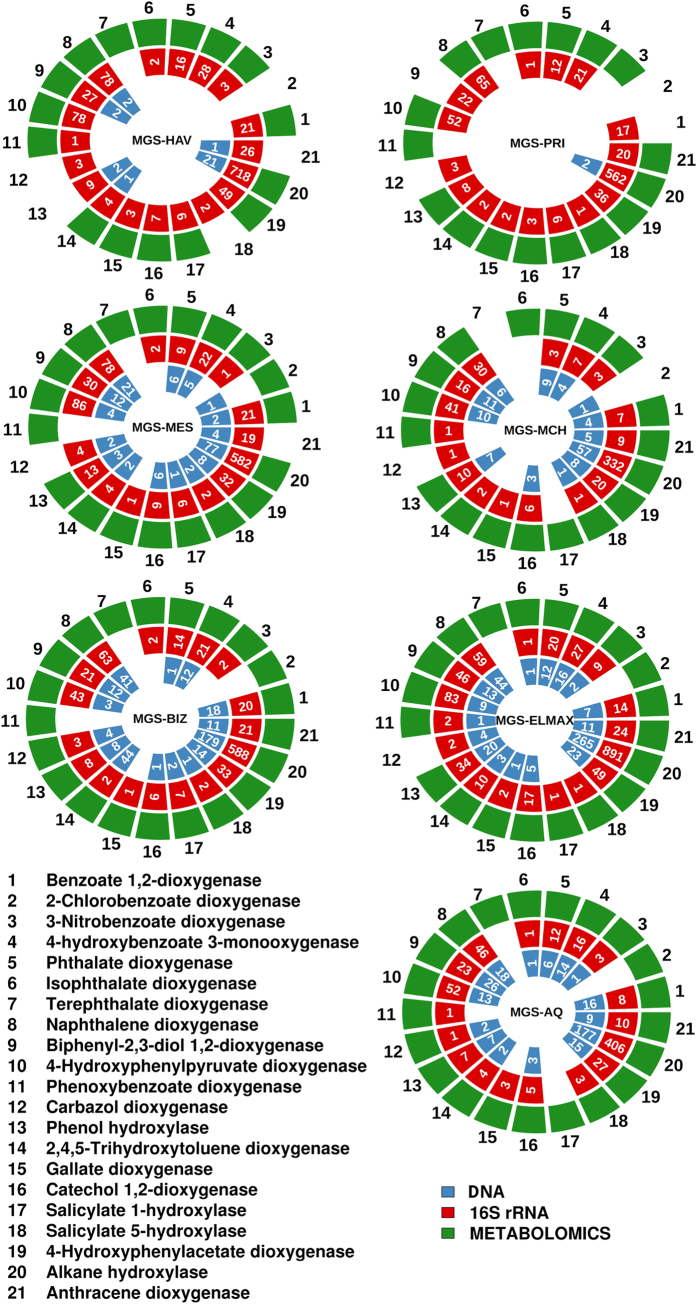
Enrichment and metabolomics-based experimental validation (green color) of degradation capacities mediated by presumptive enzymes encoded by catabolic genes expected on the basis of DNA (blue color) and 16S rRNA (red color) data sets. The number of genes encoding catabolic enzymes per each of the datasets is given inside the colored boxes. Briefly, enrichment cultures in ONR7a medium were performed, as described in Supplementary Methods, for each of the sediment samples in the presence of a pollutant mixture (10 mM total concentration) containing the following pollutants as a unique carbon source: naphthalene, anthracene, 2,3-dihydroxybiphenyl, 3,4-phenoxybenzoate, carbazole, phenol, 2,4,5-trihydroxytoluene, gallate, tetradecane, benzoate, 4-chlorobenzoate, 3-nitrobenzoate, 4-hydroxybenzoate, phthalate, isophthalate, terephthalate, and 4-hydroxyphenylpyruvate. Triplicate cultures for each duplicate sediment samples per site were set up. Two control experiments (in triplicates) were used: i) cultures without the addition of sediments but with chemicals; ii) cultures plus sediments but without the addition of chemicals. After 3 weeks of incubation, the rel. ab. of mass signatures of all the tested pollutants (data available in Supplementary Table S7A) and 9 key degradation intermediates including catechol, chlorocatechol, salicylate, muconate, gentisate, protocatechuate, homogentisate, myristate and homoprotocatechuate (data available in Supplementary Table S7B) was linked to the presence of 21 key genes encoding catabolic enzymes involved either in their degradation (in case of initial pollutants) or their production (in case of intermediates). See Supplementary Methods for descriptions of the links. Quantification was performed by target analysis using GC-Q-MS and LC-QTOF-MS. Colored box indicates DNA-, 16S rRNA- or metabolite-based signatures for a given catabolic gene. Confidence greater than 90% as indicated in Supplementary Table S5. Note: sample ELF was not included for the validation experiment, as no DNA data sets were available.

Experimental validations were conducted to further prove whether the addition of 16S rRNA data could impact interpretation of the results. Briefly, we used a 3-week enrichment protocol to evaluate the degradation of 17 pollutants expected to be degraded based on the DNA and 16S rRNA data sets (Supplementary Table S5B). These pollutants were selected on the availability of standards and the possibility of designing appropriate analytical procedures (see Fig. 2 legend). After 3 weeks of incubation, the rel. ab. of the 17 initial pollutants and of the 9 key degradation intermediates produced during their degradation (see Fig. 2 legend) was quantified by targeted analysis by gas chromatography-mass spectrometry (GC-Q-MS) and liquid chromatography-mass spectrometry (LC-QTOF-MS). Full details for the enrichment and analytical procedures and degradation efficiency can be found in Supplementary Methods and Results and Discussion. The rel. ab. of the mass signatures of all tested pollutants (data available in Supplementary Table S7A) and key degradation intermediates (Supplementary Table S7B) can be further linked to the presence of 21 key genes encoding catabolic enzymes involved either in their degradation (in the case of initial pollutants) or their production (in the case of intermediates). As shown in Fig. 2, a good agreement between the experimental validations and 16S rRNA-based predictions were found for all samples. Such a level of agreement was not found when considering the DNA-based predictions, which is most likely due, as mentioned above, to the fact that catabolic capacities were incomplete due to low sequence coverage. Therefore, biases were not introduced by refining the catabolic network using 16S rRNA data and in fact showed the predictive power of the combined DNA and 16S rRNA approaches. Note that, based on experimental metabolomics evidences, we were able to calculate confidence values, that give an estimation of the possibility that a given chemical is degraded based on a minimum number of catabolic genes (Supplementary Fig. S4) identified in the meta-sequence data sets. Calculated values are given in Supplementary Table S5.

**Figure 3:**
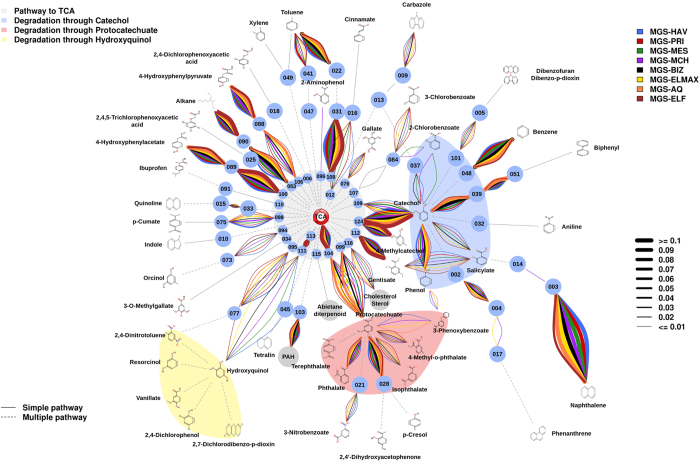
Potential aerobic and/or anaerobic degradation networks of alkanes and aromatics via di- and trihydroxylated intermediates in the investigated communities (see color code) after combining DNA- (Supplementary Fig. S2) and 16S rRNA-based (Supplementary Fig. S3) analyses. The biodegradation network reconstruction was performed as described in Supplementary Methods. Briefly, predicted open reading frames (ORFs) in the DNA or 16S rRNA-derived meta-sequences were filtered by score (>45) and e-value (<10e^−3^) according to their similarity to the sequences of key aromatic catabolic gene families involved in the degradation of aromatic pollutants and alkanes^24^,^27^. After a manual check, a final list of gene sequences encoding enzymes potentially involved in degradation was prepared. For network reconstruction, each sequence was assigned to a metabolic substrate and a product with an assigned code, and the putative substrates and products processed in the sample were connected, creating a metabolic network using appropriate scripts and commands (for details, see Supplementary Methods). The rel. ab. of each catabolic gene assigned to degradation reactions, as represented by the thickness of the lines in the figure, and the complete list of substrates possibly degraded by the communities are summarized in Supplementary Table S5. Confidence greater than 90% as indicated in Supplementary Table S5. Note that rel. ab. refers to the total number of genes in a given sample to avoid artifacts due to differences in sample size. The codes for the chemical species in each pathway are as described in Supplementary Fig. S2 and Table S5A.

According to the above considerations, a final biodegradation meta-network comprising degradation reactions assigned to genes encoding catabolic enzymes identified as present in the metagenomes (DNA) and those annotated for the most similar organisms in the communities based on 16S rRNA data was created (Fig. 3). The total rel. ab. of catabolic genes assigned to the final presumptive degradation reactions and the total number (45) and identity of substrate pollutants or intermediates possibly degraded, with a confidence of at least 90%, by each of the communities are summarized in Supplementary Table S5B. Such a biodegradation meta-network provides insight into whether each population has selected and evolved different biodegradation capacities on the basis of environmental constraints, which is discussed below.

### Temperature is the dominant factor that regulates biodegradation capacities

We found that the members of all communities have the potential to contribute to the cycling of the majority of the 45 chemical species (substrate pollutants or intermediates) found to be presumptively degraded (Fig. 3). This finding suggests that variable combinations of bacterial populations from different phyla (Supplementary Fig. S1) could fulfill overlapping and/or complementary functional roles required to complete the degradation of these pollutants. This result was also confirmed experimentally, as the mass signatures of 26 of 45 (or 58%) pollutants (17 initial substrates and 9 intermediates) were identified in the corresponding enrichment cultures (Fig. 2; Supplementary Table S7).

**Figure 4:**
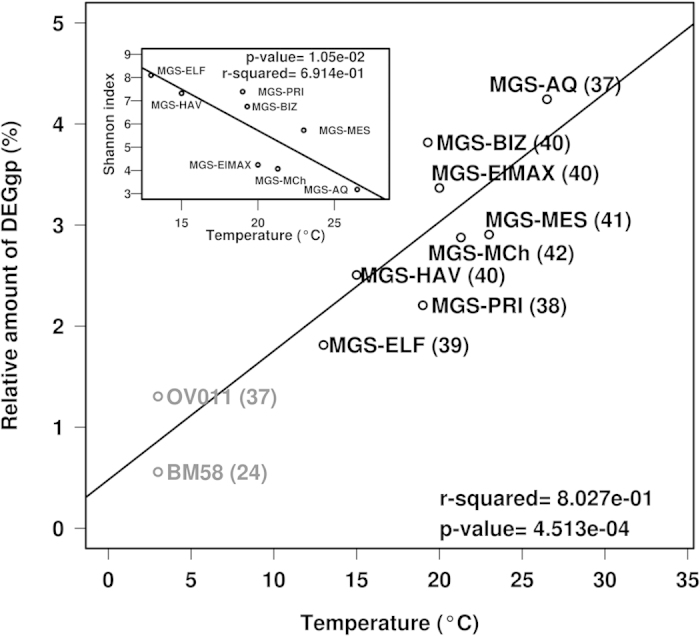
Temperature as an environmental factor driving the size of biodegradation meta-webs at the eight studied sites. A significant positive correlation (r^2^ ∼ 0.8; *P* = 4.5e^−4^*; t*-test) was found between the relative percentage of genes encoding enzymes participating in biodegradation steps (DEGgp) based on the total number of genes (to avoid artifacts due to differences in sample size) (Σ_DNA+16SrRNA_ predictions; see Supplementary Table S5B) and site temperature. The corresponding data for the BM058 and OV011 sites from the Deepwater Horizon oil spill were also included in the correlation analysis. The total number of unique polluting chemicals (initial substrates or intermediates inferred from both DNA and 16S rRNA data) presumptively accepted as substrates for enzymes in each microbial population is shown in brackets. The Shannon index, as a measure of biodiversity, negatively correlates (r^2^ ∼ 0.69; *P* = 0.0105*; t*-test) with site temperature, as shown in the inset graph. Note that a positive correlation (r^2^ ≤ 0.78; *P* ≤ 3.8e^−3^*; t*-test) was also found when considering only gene content based on 16S rRNA or DNA data sets (Supplementary Fig. S5).

As a second observation, we noted that the rel. ab. of genes encoding enzymes participating in biodegradation steps (Fig. 3) referred to the total number of genes (to avoid artifacts due to differences in sample size), ranged from 1.8% (for ELF) to 4.21% (for AQ) and was positively correlated with site temperature (Fig. 4). Thus, the highest numbers were obtained at those sites with the highest temperatures (r^2^ ∼ 0.8; *P* = 4.5e^−4^). Importantly, in contrast to the results for enzyme-coding genes, species richness negatively correlated with temperature (Fig. 4 inset; r^2^ ∼ 0.69; *P* = 0.0105). This suggests that at chronically polluted sites, lower temperature most likely results in an increase in total biodiversity (Fig. 3 inset) while promoting the loss of catabolic (i.e., pollutant degradation) biodiversity, regardless of the geographic location and other environmental constraints.

**Figure 5:**
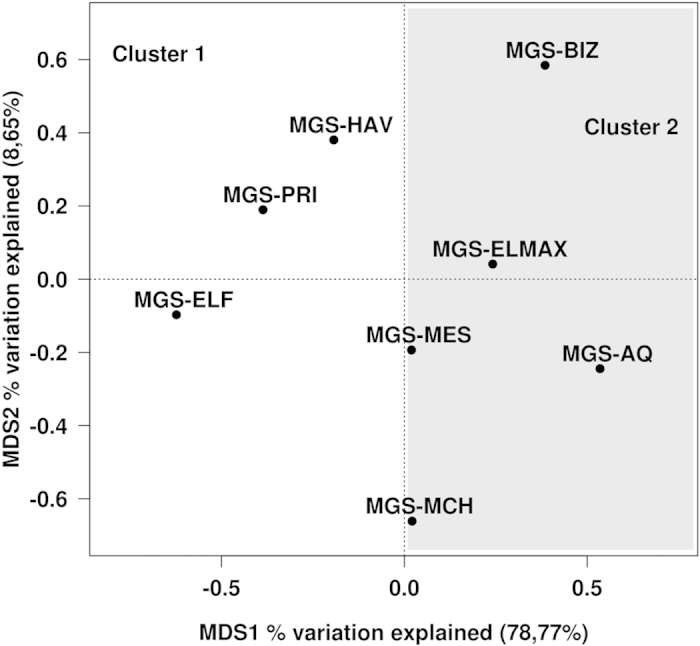
Principal coordinates analysis (PCoA) showing the clustering of catabolic gene distributions (DNA + 16S rRNA) in the Mediterranean Sea and Aqaba Gulf (Red Sea) polluted sediments. According to the sample distribution along MDS1, two clusters were identified, as indicated by light and dark grey boxes.

To explore catabolic capacity shifts between the studied sediments, principal coordinates analysis was applied to depict similarity according to the rel. ab. of genes assigned to particular reactions/pathways. As stated above, the sediments were divided into two distinct clusters (Fig. 5; *t*-test, *P* < 0.05). Sediments collected at sites HAV, PRI and ELF formed Cluster 1, whereas those collected at sites AQ, MES, MCh, ELMAX and BIZ formed Cluster 2; the latter is characterized by a higher seawater temperature (from 19.3 to 26.5 °C) compared to Cluster 1 (from 13.0 to 19.0 °C), thus supporting that temperature is also a driving factor determining the distribution of catabolic genes. Such a distribution is similar to that of the bacterial population-wide scan (Fig. 1), except for BIZ, which had a different result. This finding suggests that a transition in biodegradation capacity may occur around 19–20 °C, though further evidence is required to confirm this assertion.

**Figure 6:**
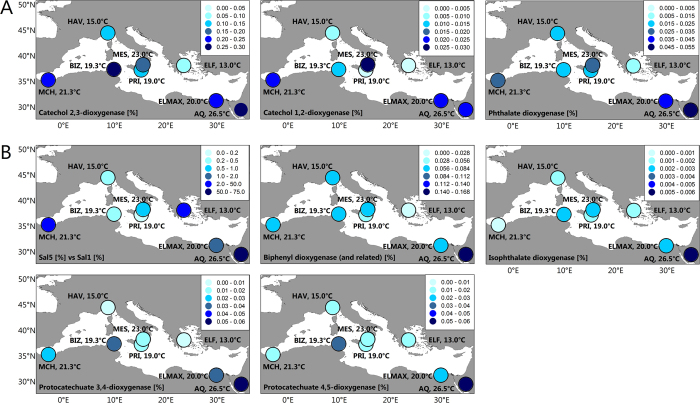
Multi-panel map of the spatial distribution of catabolic genes abundance (DNA + 16S rRNA) in the study area. Panels (A) and (B) represent genes most representative of low- and high-temperature sites, respectively. Site temperatures are indicated in the panels. The values are represented by colored dots. See the legend in each panel as a reference. Spatial distributions of gene percentages in the study area (for details see Supplementary Table S5) were produced using Golden Software Surfer 8.0. The data are plotted as colored dots showing the true values at each sampling station. Note, than in Panel B, the first map illustrates the relative percentage of genes encoding salicylate-5-hydrolyases (Sal5) as compared to salicylate-1-hydroxylases (Sal1). Reactions associated to genes encoding enzymes in panels, as follows: Catechol-2,3-dioxygenase (XylE): catechol ⇒ cis,cis-2-hydroxy-6-oxohexa-2,4-dienoate (code 124, Fig. 3); Catechol-1,2-dioxygenase (CatA): catechol ⇒ cis,cis-muconic acid (code 109, Fig. 3); Phathalate dioxygenase (OphA): phthalate ⇒ Protocatechuate; Salicylate-5-hydrolyase (Sal5 or NahGH): salicylate ⇒ gentisate; Salicylate-1-hydrolyase (Sal1): salicylate ⇒ catechol; Biphenyl dioxygenase (Bph): biphenyl ⇒ biphenyl-2-3-diol (code 51, Fig. 3); Isophathalate dioxygenase: isophthalate ⇒ protocatechuate; Protocatechuate 3,4-dioxygenase: Protocatechuate ⇒ 3-carboxy-cis,cis-muconate (code 104, Fig. 3); Protocatechuate 4,5-dioxygenase: Protocatechuate ⇒ 2-hydroxy-4-carboxymuconate-6-semialdehyde (code 099, Fig. 3).

Major differences were found in the catabolism of at least 20 substrate pollutants. Among them, the effect of temperature is particularly noticeable when examining the rel. ab. of genes encoding enzymes acting on catechol and phthalate (Fig. 6A). Thus, the rel. ab. of CatA catechol 1,2-intradiol (*t*-test; *P* = 0.0181) and XylE catechol 2,3-extradiol (*t*-test; *P* = 0.01965) ring-cleavage dioxygenases as well as OphA phthalate dioxygenases (*t*-test; *P* = 0.01301) positively correlated with the site temperature: the lowest rel. ab. was found at sites with the lowest temperatures (here, ≤19.3 °C) (Fig. 6A). High temperature was also found to be an important driver for the establishment of bacterial species with preferential capacity to degrade naphthalene via gentisate (through NahGH salicylate 5-hydroxylases) rather than via catechol (through salicylate 1-hydroxylases) (Fig. 6B). This was particularly noticeable in the two aerobic sites with the highest seawater temperature, namely, MES (21.3 °C) and AQ (26.5 °C), where only NahGH but not salicylate 1-hydroxylases were found. Note both genes encoding enzymes were found in other sediments, albeit to different extents (Fig. 6B). Temperature was found to also be a relevant factor affecting the presence and abundance of genes encoding catabolic enzymes involved in catechol (by routes others than that from naphthalene) and protocatechuate production, which was most evident in the warmest (26.5 °C) AQ site. Thus, genomic signatures were identified that suggest that all communities exhibited the potential to produce catechol through phenol (via PhO), biphenyl (via Bph and Ben), benzene (via benzene dioxygenase) and polycyclic aromatics (via 1-hydroxy-2-naphthoate dioxygenase, and Dfd), with the first three substrates being those pollutants that are most likely to result in catechol production (Fig. 3). However, biphenyl to catechol conversion (via Bph) was particularly enriched at the site with the highest seawater temperature (AQ site): up to 6.54-fold compared to the abundance at other sites (Fig. 6B). In addition, all communities also displayed the potential to produce protocatechuate from phthalate (via OphA), iso-phthalate (via isophthalate dioxygenase), methyl-phthalate (via aromatic demethylase), 4-hydroxybenzoate (via 4-hydroxybenzoate 3-monooxygenase) and 3-phenoxybenzoate (via phenoxybenzoate dioxygenase) (Fig. 3). However, the conversion of phthalate (via OphA) and isophthalate (via isophthalate dioxygenase) to protocatechuate was especially enriched at the AQ site (up to 3.23-fold; *P* = 5.4e^−3^) compared to the relative contribution at other sites (Fig. 6B). Gene signatures for the further degradation of protocatechuate via protocatechuate 4,5-dioxygenase and protocatechuate 3,4-dioxygenase were observed at all sites to a similar degree, though these genes were both most abundant at the AQ site (from 1.85- to 4.94-fold, depending the site; *P* = 1.2e^−5^) (Fig. 6B).

The spatial distributions of genes encoding enzymes supporting other distinct biodegradation capacities in the study areas are summarized in Supplementary Fig. S6.

### Genes encoding enzymes for crude oil degradation are prevalent in chronically polluted sites independently of environmental constraints

**Figure 7:**
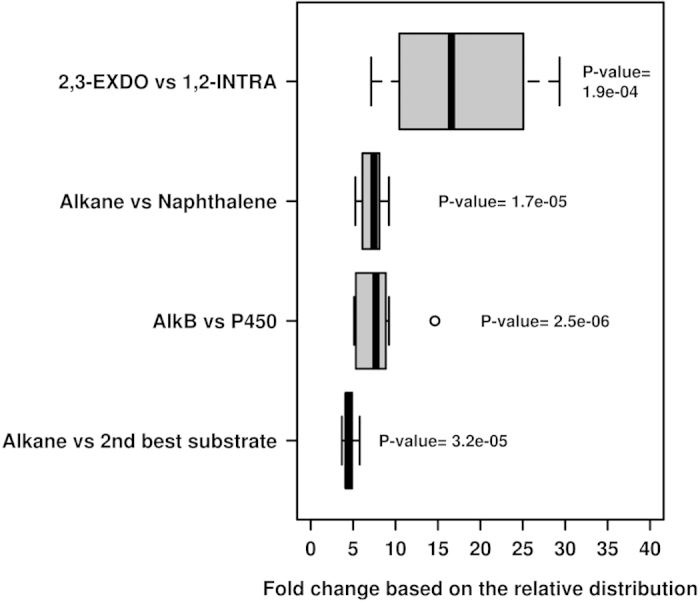
Box plot of the relative contribution of genes involved in degradation steps. Percentages were calculated on the basis of the data obtained from the DNA and 16S rRNA gene content analysis. *P*-values correspond to Student’s *t*-test of the average of the relative contribution of the enzyme classes, as based on the Welch approximation.

A number of major common catabolic features were observed for all sites, independent of the environmental constraints, when analyzing the rel. ab. of catabolic genes (Fig. 3; Supplementary Table S5). First, alkanes were predicted to be the best pollutant substrates for all communities, as based on the overabundance of genes encoding alkane-degrading enzymes (AlkB and, possibly, P450) compared to the second most abundant gene class, namely, those encoding gentisate (in HAV, PRI and MES)- and catechol (in the remaining samples)-modifying enzymes (4.53-fold average; *P* = 3.2e^−5^) (Fig. 7); note that the rel. ab. level of AlkB- and P450-coding genes ranged from 0.68% (for ELF) to 1.36% (for AQ) and that AlkB was, on average, 7.93-fold (*P* = 2.5e^−6^) more abundant than P450 in all samples (Fig. 7). This suggests that alkanes are more efficiently degraded than recalcitrant polycyclic aromatics in the chronically polluted sites investigated here. A high rel. ab. level of genes encoding fatty acid hydroxylases, ranging from 0.016% (for PRI) to 0.065% (for BIZ), was also observed. These results are in agreement with previous studies that demonstrated that AlkB-coding enzymes and related enzymes (e.g., alkane hydroxylases) are the most prevalently expressed at elevated alkane concentrations^2^,^9^,^11^. This finding supports the consideration that chronic pollution, regardless of the crude oil input (from 116 ppm at the BIZ site to 260,000 ppm in HAV; Supplementary Table S1), also results in a preferential enrichment of bacteria possessing genes involved in alkane degradation (Fig. 3 and Fig. 7), regardless of the geographic location and environmental constraints. Accordingly, we speculate that the microbial populations inhabiting chronically polluted sites might be highly adapted, and respond more rapidly, to the degradation of petroleum components after an accidental oil spill than populations at ‘clean’ seawater, where *alk*B genes constitute minor components^2^,^9^,^11^. Second, the rel. ab. level of genes encoding enzymes for the degradation of polyaromatic compounds (such as Ndo, PhdA, Dfd) was, on average, 5.87-fold lower (*P* = 3.55e^−6^) than that of AlkB (Fig. 7), as was also reported at the Deepwater Horizon oil spill^2^. A third common attribute was an overabundance of genes encoding XylE catechol 2,3-extradiol (EXDO) compared to CatA catechol 1,2-intradiol (INTRA) ring-cleavage dioxygenases (19.78-fold average; *P* = 1.9e^−4^; Fig. 7), which suggests that EXDO cleavage processes are of greater importance than INTRA cleavage processes at all the studied sites, regardless of geographic location and environmental constraints.

### Biodegradation signatures in the Deepwater Horizon oil spill

**Figure 8:**
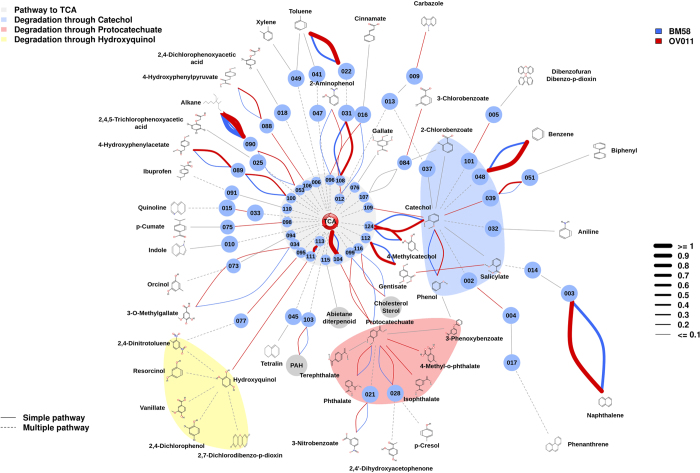
Potential aerobic and/or anaerobic degradation networks of alkanes and aromatics via di- and trihydroxylated intermediates in the investigated communities in the BM058 and OV011 sites within the Deepwater Horizon oil spill. The color code used for the respective pathways is shown. Confidence greater than 90% as indicated in Supplementary Table S5. For details about the graphical representation of the network, the thickness of the lines and the codes for the chemical species, see the legend to Fig. 3.

Accumulated evidence has demonstrated differences in microbial community structures and biodegradation gene contents in different plume sites within the Deepwater Horizon oil spill^2^,^12^; the differences relate to the amount of time that the respective sites were exposed to hydrocarbons as well as to the crude oil concentration. Two different samples from the Deepwater Horizon oil spill, BM058 (Longitude: −88.4375; Latitude: 28.672222; JGI project ID 403207; taxon IDs 2088090017 and 2081372002) and OV011 (Longitude: −88.4375; Latitude: 28.672222; JGI project ID 403191; taxon ID 2081372001), the metagenomic contents of which have been recently reported^2^,^12^, were further analyzed using the meta-network procedure applied in this study. To this end, meta-sequences corresponding to samples BM058 and OV011 were obtained from the Joint Genome Institute webpage (https://img.jgi.doe.gov/; see accession numbers above) and analyzed in the same way as the meta-sequences generated in this study. A total of 1,174 of 137,924 potential protein-coding genes covering unique proteins were identified. The rel. ab. of sequences presumptively involved in biodegradation compared to the total number of sequences (Supplementary Table S5A) was 2.3-fold higher at the OV011 site (1.30%) compared to BM058 (0.56%), in agreement with the closer location of OV011 to the wellhead of the oil spill^6^ and with previous observations demonstrating that genes encoding enzymes for biodegradation are prevalent in the plume^2^. Altogether, this result suggests that the meta-network approach applied in the study can be of interest for investigating catabolic diversity in any type of sample. The diversity of genes encoding catabolic enzymes further reveals genomic evidence for the presumptive metabolism of 37 (for OV011) and 24 (for BM058) different substrates and/or their intermediates (Fig. 8; Supplementary Table S5B), indicating that the hydrocarbon input also positively influenced the size of the biodegradation meta-network. A comparison of the OV011 and BM058 meta-webs further revealed the absence of the presumptive genes encoding XylE catechol 2,3-extradiol dioxygenase at BM058, suggesting a positive correlation between the presence of XylE genes and the concentration of crude oil (or the distance to the plume). This agrees with the fact that compared to CatA catechol 1,2-intradiol dioxygenase, XylE genes were most abundant in the chronically contaminated sites investigated herein (Fig. 7).

A direct comparison between the Mediterranean and Red Sea sites and those at the Deepwater Horizon oil spill first highlighted that the number of presumptive substrate pollutants (≤37) is slightly lower than those potentially metabolized in the eight studied sites in the Mediterranean and Red Seas (45 in total, Fig. 3; Supplementary Table S5B). Second, the rel. ab. of total sequences encoding catabolic enzymes in the OV011 (1.30%) and BM058 (0.56%) sites was significantly lower than that in the chronically polluted sites investigated (Fig. 4). This is in agreement with the lower temperature at the Deepwater Horizon oil spill (<3 °C)^2^,^6^, compared to those at the sites investigated herein (from 13.0 to 26.5 °C) and the loss of catabolic genes at the lower temperatures demonstrated in this study (Fig. 4). Notably, among catabolic genes, AlkB-, P450- and fatty acid hydroxylase-coding genes, presumptively involved in the initial degradation of crude oil components, accounted for a total of 0.65% (or 50% of the total) and 0.23% (or 41% of the total) at OV011 and BM58, respectively; however, the rel. ab. was higher in the chronically polluted sites: from 0.81% (for PRI) to 1.46% (for AQ). This suggests that chronic pollution may also positively influence the abundance of genes encoding proteins for the initial degradation of major crude oil components such as alkanes.

### Metabolome-wide scan revealed strong ecotype-chemical species associations

**Figure 9:**
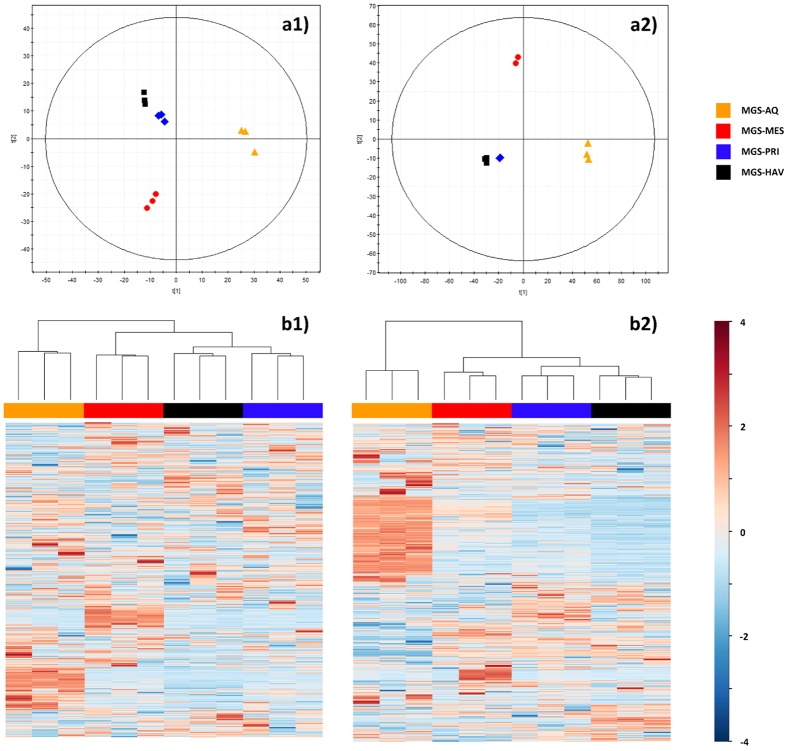
Metabolite profiles in chronically-polluted sites. **a**) Principal Components Analysis (PCA) plot for models built with the filtered set of metabolome data (metabolites extracted in triplicates). a1) LC-MS (−): 2 components, R^2^ = 0.338, Q^2^ = 0.013. a2) LC-MS (+): 2 components, R^2^ = 0.491, Q^2^ = 0.210. **b**) Hierarchical clustering analysis performed with the filtered masses, whereby abundances were scaled by mean-centering and dividing by the standard deviation of each variable. b1) LC-MS (−). b2) LC-MS (+).

The data presented above suggest that environmental constraints, particularly temperature, impact biodiversity and pollutant-degrading populations and catabolic web structures. We further examined whether the chemical diversity at the sampling sites, which may be a direct consequence of chronic pollution^16^, also had a significant effect on ecosystem diversity and catabolism. Rather than examining the chemical diversity of crude oil components^4^, we analyzed a large number of metabolites by a combination of mass spectrometry (MS) with liquid chromatography (LC) separation, which yields rapid and quantitative results in a single analysis in a non-biased manner^28^. Due to sample limitations, only four samples (PRI, HAV, MES and AQ) could be examined. Nonetheless, these samples are representative of the clusters previously identified and are characterized by moderately low (15–19 °C) to moderately high (23–26.5 °C) temperatures, a high level of chronic pollution (≥1,000 ppm total petroleum hydrocarbons) and a wide range of O_2_ concentrations (from anoxic to 20.0 mg/L). Metabolites were directly extracted from the sediment samples (see Supplementary Methods) and analyzed. The profiles obtained were complex due to the high number of metabolite signals present in the samples (Supplementary Table S8). During data treatment (*t*-test, *P* < 0.05), the list of significantly different masses obtained in positive mode was reduced from 114,050 after alignment to 3,390 after filtering and from 54,358 to 1,485 in LC-MS negative mode. The filtering criteria were based on the selection of masses that were present in at least 100% of the replicate samples per group. Classification of the samples revealed clusters (Fig. 9) in which PRI and HAV were the most similar with respect to chemical diversity. The robustness of the analytical procedure was demonstrated by the tight clustering of the sample replicates (Fig. 9, panels A1 and A2), confirming that the separation among the groups was due to real biological variability and was not generated randomly. A direct comparison with the clusters found by examining the bacterial diversity established by the 16S rRNA pyrotag analysis (Fig. 1) and the distribution of genes encoding enzymes involved in biodegradation (Fig. 4) revealed similar trends as for the chemical species (Fig. 9).

## Discussion

Polluted environments generally host a finely tuned restricted set of microbes^5^,^6^, with the phylotype richness depending highly on the chemical species released to the environment and the pollution level^1^,^2^,^3^,^4^. Moreover, multiple environmental factors have been shown to alter marine microbial communities^7^,^8^,^9^,^10^,^11^,^12^. Nonetheless, it remains to be determined how multiple environmental pressures impact such populations and which of these, if any, is the dominant driving factor determining the final catabolic outcomes. Of particular significance is that information on microbial populations and functional content in marine ecosystems where oil spills have occurred is limited in the specialized literature for the Mediterranean and Red Seas, both of which receive major crude oil inputs^16^. This study went beyond descriptive studies of community composition^16^ and utilized taxonomic bar-coding, metagenomic prediction platforms for inferring biodegradation activities, and a metabolome-wide scan to provide deeper insight into the common and distinctive populations and biodegradation biomarkers and deficits associated with seven geographically separated major oil-polluted sites along the coastlines of the Mediterranean Sea and one from the Red Sea. The total concentration of hydrocarbons in the sediments exceeds that of clean seawater (15 ppm) by at least 7.7- to 17,000-fold (Supplementary Table S1), supporting the importance of investigating the degradation processes occurring in polluted sediments, which are influenced by accumulation phenomena.

The results of this study draw attention to the huge undiscovered pool of bacterial species populating the Mediterranean Sea, including the northern and southern parts of the basin, and the Gulf of Aqaba in the Red Sea. These microorganisms might play important roles in the cycling of prevalent and persistent pollutants as well as in total carbon cycling. We demonstrated that the microbial populations in the chronically polluted sites investigated have the presumptive capacity to degrade at least 45 polluting chemicals, and the ability to degrade 26 of these substrates (including initial pollutants and intermediates) was experimentally confirmed in microcosms using targeted metabolomics. This number is slightly higher (up to 1.8-fold) than that observed at the Deepwater Horizon oil spill sites. Our findings further revealed that the sites investigated herein, which are subjected to chronic anthropogenic forces (hydrocarbon input), exhibit selection for bacterial species other than the ubiquitous specialized HCB (see Supplementary Results and Discussion). In addition, although the relative percentage of gene sequences encoding enzymes for biodegradation range from 0.56 to 1.30% in the cold (<3 °C)^2^,^12^ OV011 and BM058 sites at the Deepwater Horizon oil spill, this number, which increased with site temperature, was up to 5.6-fold higher in the moderately warmer sites examined in this study (from 13 to 26.5 °C) (Fig. 4). This finding is of practical importance because it suggests that compared to bacteria inhabiting clean sites where random or accidental oil spills occur, warmer chronically polluted sites might promote the establishment of catabolically adaptable microbial populations, other than HCB, that can respond to an accidental oil spill to a higher degree than previously thought. In relation to this, our results also suggest that approximately half of the genes encoding catabolic enzymes in the Deepwater Horizon oil spill sites, referred to as the total genes, encode enzymes mediating the initial attack of alkanes. Conversely, in the chronically polluted sites of the Mediterranean and Red Seas examined here, such genes accounted for approximately 33–36%, suggesting that the chronic exposure to pollutants may have played a role in catabolic diversification (Fig. 3 and Fig. 7).

We are aware that OV011 and BM058 are highly polluted samples, as they were collected from the Gulf of Mexico approximately one week after the Horizon Oil Spill occurred (cruise date: 27.04.2010–2.06.2010)^2^,^12^; however, they can also be considered to be characterized by a state of natural chronic pollution from crude oil seepage. Therefore, it is also plausible that the different chemical species accumulated in the naturally influenced deep Gulf of Mexico (yet to be investigated) compared to the more anthropogenically influenced sites examined herein may play a role in the observed differences. The fact that the differences reported here may also be derived from the distinct types of samples that were compared (sediments in this study vs seawater in the OV011 and BM058 samples) cannot be ruled out. Regardless, it should also be highlighted that some of the dominant presumptive bacteria and catabolic genes identified herein may not be the most active degraders in the examined sediment samples, as has been recently demonstrated when comparing untreated and bio-stimulated soils^29^, and that the determination of their biodegradation efficiency will require further investigation.

It should also be noted that the sequencing depth (≤2.9 Gbp per sample) presented here may be considered low. However, the main objective of the metagenomic survey was not to access an extensive sequencing depth but rather to obtain sufficient sequence data to allow a determination of whether distinct biodegradation capacities might be linked to environmental constraints and community shifts, which was achieved in the present investigation. This issue was partially resolved by examining genomes established on the basis of phylogenetic affiliations^23^ performed at a higher depth. Our study is also the first to highlight the profound shifts in taxonomic and catabolic ecotypes in both basins, which apparently were highly influenced by the seawater temperature, independent of the other environmental constraints (i.e., O_2_ and total hydrocarbon concentration). Although temperature has been demonstrated to contribute to marine and soil microbial diversity evolution^30^,^31^, our data show for the first time its association with the specific loss (low temperature) or gain (high temperature) of catabolic biodiversity as well as with the modulation of the microbial capacity to preferentially degrade certain aromatic pollutants (e.g., naphthalene, gentisate, catechol, phthalate). As an example, the sites with lower temperatures contained the highest bacterial diversity while being characterized by a lower presence of catabolic genes, particularly for catechol and phthalate metabolism. This suggests a positive impact of lower temperature on the total bacterial population while having a marked negative effect on catabolic diversity. In addition, our data suggest that the accumulation of chemical species in the semi-enclosed Mediterranean basin and the Red Sea might contribute to the establishment of such temperature-guided populations and the channeling recalcitrant accumulated pollutant substrates into distinct catabolic pathways. Recently, it has been demonstrated that the abundance of pollutant-degrading bacteria decreased as a consequence of biological treatments such as bio-stimulation and supplementation with pollutants in slurry-phase bioreactors operating for over 3 years at 23 °C^29^. This contrasts with the results of the present study, pointing to the catabolic diversification as a consequence of chronic pollution. It is plausible that the differences in the time frames (decades of contamination in the sites investigated herein vs 3 years) and the chemical diversity (multiple pollutants and chemicals in natural sites vs one (anthracene) in the reported enrichment culture) in the two investigations may be responsible for the observed variation. In addition, the differences could also be due to the fact that Dunlevy and cols^29^ investigated phenomena occurring in a bio-reactor-scale system in which enrichment cultures (with their limits) were analyzed, whereas our study reports trends occurring in the environment.

Taken together, for chronically polluted sites, investigating the sediment and water temperature might reveal regular patterns of behavior with predictive value. We believe that these findings, for which no previous evidence exists in the scientific literature, potentially open new research avenues for investigating novel site-tailored bioremediation approaches based on site characteristics as well as for establishing global biodegradation webs based on genomic signatures, pollutant types and geoclimate constraints.

## Methods

### Experimental settings and data analysis

The full description of the materials and methods used for the: a) environmental measurements, sample collection, and nucleic acid extraction, b) SSU rRNA hypervariable tag analysis, including the workflow scripts and commands, c) DNA sequencing and gene calling, d) biodegradation network reconstruction, including description of scripts and commands for graphics, e) enrichment cultures and target metabolomics for experimental validations of biodegradation capacities, f) metabolomic fingerprint analysis of sediment samples, and g) cell counts is available in the Supplementary Methods.

## Additional Information

**Accession numbers.** The projects have been registered as an umbrella BioProject at NCBI with the IDs PRJNA222659 [for MGS-HAV], PRJNA222657 [for MGS-MES], PRJNA222658 [for MGSPRI], PRJNA222660 [for MGS-BIZ], PRJNA222661 [for MGS-MCh], PRJNA222667 [for MGS-AQ], PRJNA222665 [for MGS-BIZ], and PRJNA222666 [for MGS-ELMAX]. The Whole Genome Shotgun projects have been deposited at DDBJ/EMBL/GenBank under the accession numbers AZIB00000000 [for MGS-HAV], AZIC00000000 [for MGS-MES], AZIF00000000 [for MGS-PRI], AZID00000000 and AZII0100000 [for MGS-BIZ], AZIE00000000 [for MGS-MCh], AZIG0100000 [for MGS-AQ], and AZIJ0100000 [for MGS-ELMAX]. All original non-chimeric SSU rRNA hypervariable tag 454 sequences are archived at the EBI European Read Archive under accession number PRJEB5322. Note that code ‘MGS’ refers to MetaGenome Source (see Supplementary Methods)

**How to cite this article**: Bargiela, R. *et al*. Bacterial population and biodegradation potential in chronically crude oil-contaminated marine sediments are strongly linked to temperature. *Sci. Rep*. **5**, 11651; doi: 10.1038/srep11651 (2015).

## Supplementary Material

Supplementary Information

Supplementary Table S1

Supplementary Table S2
