# Supplementary material for: Bacterial population and biodegradation potential in chronically crude oil-contaminated marine sediments are strongly linked to temperature
Source: Sci Rep. 2015 Jun 29;5:11651. doi: 10.1038/srep11651 (PMC4484246; doi:10.1038/srep11651)
Supplement: Supplementary Information [file srep11651-s1.pdf]

Bacterial population and biodegradation potential in chronically crude oil-contaminated marine sediments are strongly linked to temperature

Rafael Bargiela, Francesca Mapelli, David Rojo, Bessem Chouaia, Jesús Tornés, Sara Borin, Michael Richter, Mercedes V. Del Pozo, Simone Cappello, Christoph Gertler, María Genovese, Renata Denaro, Mónica Martínez-Martínez, Stilianos Fodelianakis, Ranya A. Amer, David Bigazzi, Xifang Han, Jianwei Chen, Tatyana N. Chernikova, Olga V. Golyshina, Mouna Mahjoubi, Atef Jaouani, Fatima Benzha, Mirko Magagnini, Emad Hussein, Fuad Al-Horani, Ameur Cherif, Mohamed Blaghen, Yasser R. Abdel-Fattah, Nicolas Kalogerakis, Coral Barbas, Hanan I. Malkawi, Peter N. Golyshin, Michail M. Yakimov, Daniele Daffonchio, Manuel Ferrer

SUPPLEMENTARY MATERIAL

Supplementary Results and Discussion

Bacterial diversity through 16S rRNA pyrotag analysis: general comments. Overall, Proteobacteria was the most abundant (50-100% total sequences) at all sites with the exception of MCh, which was dominated by Bacteroidetes (77.9% total sequences) (Supplementary Fig. S1). The bacterial community at the AQ site contained the highest percentage of Proteobacteria (100%), whereas this phylum was detected at the lowest level (below 50%) at the MES and MCh sites. Note, that no sequences in our dataset were affiliated with the genus *Alcanivorax* or other typical specialized hydrocarbonoclastic (HCB) bacteria. The lack of detection of such species may be due to the very high relative abundance of other genera, as reported in the Deepwater Horizon oil spill[3], that are likely more adapted to the unique environmental constraints characterizing the investigated sites. Note also that, although bacteria of the genus *Alcanivorax* were not enriched during the Deep Horizon Oil Spill, in some cases sequences and/or cultured isolates were detected, which may also be possible to occur in the samples herein analysed.

Among those bacteria belonging to the Proteobacteria phylum, Gammaproteobacteria was the predominant class in the sediments AQ, HAV, PRI, MES, and BIZ (comprising 87.6, 55.8, 33.4, 31.3, and 23.4% of the total bacterial community, respectively) (Supplementary Fig. S1B). In addition, Gammaproteobacteria was also the richest class in term of diversity and was represented by 17 different genera/unclassified families (Supplementary Table S3), including *Marinobacter*, *Pseudoalteromonas*, and *Cycloclasticus*, which comprise populations well known for their oil biodegrading capabilities[32,33]. The sediment of the AQ site was primarily colonized by the genera *Alteromonas* and *Psychrobacter*, which represented 20.5 and 63.7% of the total bacterial community, respectively. Mercury-resistant *Psychrobacter* strains were previously isolated from the sediment of a coastal lagoon in Italy and have been suggested as a possible tool in the bioremediation of mercury-contaminated sediments[34]. The adaptation of this genus to a broad class of pollutants is further suggested by the ability of *Psychrobacter* strains isolated from Antarctica to degrade poly-chlorinated biphenyls (PCB) in the laboratory[35]. The isolation of strains able to degrade naphthalene, phenanthrene, anthracene, and pyrene during microcosm assays suggests that the genus *Alteromonas* was a key player during the biodegradation of PAH during an oil spill along the Korean shoreline[36].

The bacterial community of the polluted sediments collected at site ELF primarily comprised Deltaproteobacteria (47% of the total bacterial community, Supplementary Fig. S1B), which was represented by six different genera (Supplementary Table S3). A high abundance of Deltaproteobacteria primarily represented by sulfate-reducing bacteria (SRB) was previously

reported for other natural environments characterized by the presence of oil hydrocarbons and anoxic conditions. These environmental conditions favor the establishment of bacteria belonging to the cluster *Desulfosarcina-Desulfococcus*, which are involved in oil biodegradation processes[37,38], and bacteria belonging to the family *Syntrophobacteraceae*, which has been implicated in metal attenuation and retention in sediments[39] and constituted 24.7% of the total community in the ELF site. Deltaproteobacteria were also detected at the HAV site (6.6% of the bacterial community, Supplementary Fig. S1B), where the phylum Proteobacteria comprises 92% of the bacterial community (Supplementary Fig. S1A), encompassing all the known classes except for Zetaproteobacteria. Sequences affiliated with the class Betaproteobacteria were detected exclusively at this site (Supplementary Fig. S1B), where the most abundant class was Gammaproteobacteria, including, among others and in addition to the prevalent group of unclassified sequences (42.1% of the bacterial community), the oil-degrading genera *Pseudoalteromonas* and *Cycloclasticus* (Supplementary Table S3). A similar bacterial community composition was observed at the PRI site, where Delta-, Epsilon-, and Gammaproteobacteria were the major phylogenetic groups (Supplementary Fig. S1B). Moreover, among the most abundant members of the proteobacterial communities at the MCh site, the *Arcobacter* genus, belonging to the Epsilonproteobacteria, represented 7% of the sequences detected by bar-coded pyrosequencing. This genus was recently cultivated from oil-polluted sediments in enrichment cultures supplemented with phenanthrene[40]. Finally, at the ELMAX site, the most abundant class was represented by Alphaproteobacteria (Supplementary Fig. S1B), a phylogenetic class widespread in sea-water samples[41] and comprising, in our dataset, 14 different genera (Supplementary Table S3) that formed 46.3% of the total bacterial community.

Apart from Proteobacteria, we found that other members were significantly enriched. The ElMAX bacterial community also contained a high percentage of sequences affiliated with the genus *Bacillus* (Supplementary Table S3), whose presence has been already reported in several hydrocarbon-degrading consortia[37,42]. MCh was dominated by the class Flavobacteria of the phylum Bacteroidetes (77.9%; Supplementary Fig. S1A). The Bacteroidetes phylum is frequently found in nutrient-rich habitats and has been recognized as a key actor in the carbon cycle in marine environments due to the ability of its members to degrade high-molecular-weight organic matter and biopolymers such as protein and polysaccharides[43]. The first genomic data available for the Bacteroidetes group was related to the genus *Gramella*[43], which represented 4.1% of the total bacterial community at the MCh site (Supplementary Table S3). Bacteroidetes at the MCh site was primarily represented by the genus *Salinimicrobium* (Supplementary Table S3), formerly isolated from saline soils tidal flat sediments[44]. The Bacteroidetes phylum was also abundantly present at the MES site, together with the classes Planctomycetes (Supplementary Fig. S1A-B)

and Gammaproteobacteria, which belong to the family Piscirickettsiaceae (Supplementary Table S1). A high percentage of the bacteria colonizing the MES sediment belonged to the candidate division OD1 (Supplementary Table S3) that was previously found in anoxic, sulfur-rich aquatic ecosystems[45] in which their ecological role remains unknown. Finally, Planctomycetes of the order Phycisphaerales was particularly abundant in the MES site (Supplementary Table S3). A preponderance of the Proteobacteria phylum was also observed at the lagoon of BIZ site (Supplementary Fig. S1), although at this site, Flavobacteria was the most abundant class (Supplementary Table S3), similar to MCh.

Biogeography of total and crude oil degrading bacterial populations

Low temperature (here, ≤ 19.3°C) and restricted oxygen (here, ≤ 6.5 mg/L) seems to favour the establishment of bacteria belonging to seven different genera/unclassified families (Supplementary Table S3). Those belonging to the families *Desulfobulbaceae*, *Helicobacteraceae* and *Acidimicrobiales* developed primarily (from 0.33 to 18.09% total sequences) at sites with lower temperature (here, ≤ 19.3°C) and restricted oxygen (here, ≤ 6.5 mg/L), namely, ELF, HAV, PRI and BIZ, which formed the Cluster 1 described in Fig. 1. The low relative representation (≤ 0.047%) of these bacterial families at ELMAX (20.0°C), which had a high O_2 concentration (18.0 mg/L), and their absence in the moderately warmer MES site (23.0°C), which was micro-anaerobic (O_2: 1.0-2.2 mg/L), support members of these families associated to cold- and O_2-restricted sediments. In addition, bacteria belonging to the families *Syntrophobacteraceae* and *Thermodesulfovibrionaceae* (from 0.1 to 24.7%) were only found at sites with a temperature ≤19.0°C (ELF, HAV and PRI), and those belonging to the families *Phycisphaerae* and *Desulfobacteraceae* (from 0.1 to 2.3%) at the sites with temperatures ≤ 15.0°C (ELF and PRI).

In addition to temperature, other factors, alone or in combination with temperature, are also important secondary factors that influenced the distribution of particular sets of bacterial groups. As summarized in Supplementary Table S3, ELMAX contained the highest number (14, representing 74.1% total sequences) of habitat-specific groups, followed by HAV and MES (3 each, representing 11.98% and 11.47%, respectively), MCh and AQ (2 each, representing 13.53% and 64.39%, respectively) and BIZ (1, accounting 1.85%), whereas none was exclusive at the PRI and ELF sites. Among habitat-specific groups, members unambiguously classified within the genera/families *Caulobacteraceae, Bradyrhizobiaceae, Methylobacteriaceae, Sinobacteraceae*, TM7, *Propopnibacterium, Methylobacterium, Bradyrhizobium, Planomicrobium, Chitinophagaceae, Staphylococcu*s, *Anaerococcus*, *Thiomicrospira*, and *Corynebacterium* (in order from higher to lower percentage of sequences referred to the total) were only found in ELMAX, AB16 in HAV, *Spirochaetes* in BIZ, *Gramella* and *Bacillacea* in MCh, *Mesorhizobium* and candidate division OD1 in

MES and *Nautella* and *Psychrobacter* in AQ. In addition, notable dominance of members of the genera *Salinimicrobium* at the MCh site (73.8% total sequences), *Alteromonas* (20.5%) at the AQ site and of the class Planctomycetes (15.86%) at the MES site, were also observed (Supplementary Table S3).

Finally, bacteria belonging to the genera *Cycloclasticus* (from 0.19 to 2.97%) and *Sulfuricurvum* (from 0.22 to 10.93%; Supplementary Table S3) were restricted to geographically related sites, namely, sites in the more northern part of the whole marine north-south transect we have considered (HAV, PRI, MES and BIZ). While *Cycloclasticus* appeared be selected (≥ 6.3-fold in terms of relative percentage of total sequences) in sites with restricted oxygen (here, ≤6.5 mg/L) and higher crude oil input (HAV; tar sample), *Sulfuricurvum* was most abundant (≥ 10.3-fold) in highly contaminated anoxic sites (PRI; 4,000 ppm total hydrocarbons). Furthermore, bacterial members associated to *Phycisphaerales* and *Piscirickettsiaceae* were only associated with more northern sites, namely HAV, PRI and MES, although it was particularly enriched (24.25%) at the warmer (23.0ºC) and micro-aerobic (1.0-2.2 mg/L O_2) MES site.

Degradation efficiency of pollutants in enrichment cultures by targeted metabolomics

Targeted GC-Q-MS and LC-QTOF-MS were used to confirm the degradation of 17 pollutants, expected to be degraded to different extents by each of the microbial populations. For that, we determined their abundance level (Supplementary Table S7A) as well as the presence and abundance of 9 key degradation intermediates produced during their degradation (Supplementary Table S7B), in enrichment cultures performed as described in Supplementary Methods. The extent of the degradation efficiency, by meaning of the remaining concentration of chemical species at the end of the three-week incubation time, as compared to the initial concentration and control tests, was calculated.

As shown in Supplementary Table S7A, for AQ all pollutants were degraded, with relative degradation values ranging from ~99 to 41%, after three-weeks incubations. For BIZ, all tested pollutants were degraded, with relative degradation values ranging from ~97 to 3.7%. For ELMAX, all tested pollutants but one (carbazole) were degraded, with relative degradation values ranging from ~98 to 29%. For HAV, all tested pollutants but four (chlorobenzoate, carbazole, phenol and anthracene) were degraded, with relative degradation values ranging from ~98 to 4.4%. For MCh, all tested pollutants but three (chlorobenzoate, terephthalate and carbazole) were degraded, with relative degradation values ranging from ~96 to 54%. For MES, all tested pollutants but two (anthracene and carbazole) were degraded, with relative degradation values ranging from ~99 to 47%. For PRI, all tested pollutants but four (benzoate, chlorobenzoate, 2,3-

dihydroxibiphenyl and carbazole) were degraded, with relative degradation values ranging from ~89 to 30%.

As shown in Supplementary Table S7B, examination of the production of intermediate species revealed that the 9 selected intermediates were significantly produced in all microcosms, except gentisate, which was not found in HAV, and chlorocatechol which was slightly produced in MCh.

Since the setup microcosms herein might not mimic environmental conditions and not all microbes present in the original community may grow under the culture conditions we cannot rule out the possibility that the experimental measurements of substrate pollutants and intermediates might not be synonymous with the presence of genomic signatures. Having said that by linking the presence of each of the chemical species with the gene encoding catabolic enzymes involved in their transformation, we were able to link metabolite data with sequencing (DNA and 16S rRNA) data sets (Fig. 2), and good agreement with our *in silico* predictions was observed. This demonstrates that the enrichment conditions herein used were appropriate to detect the degradation reactions herein evaluated.

Supplementary Methods

Sampling sites and sample codes. The investigated sites included the following, in order of latitude coordinates: (1) the Gulf of Genoa in the northernmost part of the Ligurian Sea (Genoa, Italy; 44° 22'25.75″N, 8° 41'59.58″E), where the Haven tanker sunk (*HAV*) in 1991[47]. MT Haven, formerly Amoco Milford Haven, was a very large crude carrier, leased to Troodos Shipping. In 1991, while loaded with 144,000 tonnes (1 million barrels) of crude oil, the ship exploded, caught fire and sank off the coast of Genoa (Italy), and flooding the Mediterranean with up to 50,000 tonnes of crude oil. It broke in two and sank after burning for three days and since this event the Mediterranean coast and sediments of Italy and France was polluted, especially around Genoa; (2) the harbor of Messina (*MES*) (Sicily, Italy; 38°11'42.267"N, 15°34'25.014"E), a marine harbor that generally suffers chronic petroleum pollution because of intensive maritime traffic and its limited hydrodynamic regimen and restricted area[48,49]; (3) the coast adjacent to an oil refinery unit in the Elefsina Bay (*ELF*) northwest of Athens (Greece; 38°2'16.28"N, 23°30'45.85"E), which is a contaminated shallow coast where petroleum hydrocarbons seep out intermittently from an adjacent oil refinery unit[50]; (4) the harbor of Priolo (*PRI*) Gargallo (Siracusa, Italy; 37°10'27.462"N, 15°12'7.505"E), which is characterized by heavy industrialization and intensive tanker traffic transporting both crude and refined oil[51]; (5) the Bizerte lagoon (*BIZ*) located in Northern Tunisia (37°16'08.9"N, 9°53'20.1"E), which is highly populated and urbanized and is subject to a pollution load determined by petroleum components in the area adjacent to an oil

refinery[52,53]; (6) the lagoon of Mar Chica (*MCh*), located on the north-west Mediterranean coast of Morocco (35°11'57,1"N, 2°55'37,6"O), which is among the largest lagoons in the south coast of the Mediterranean Sea and was in the past exposed to continuous pollution by the town of Nador on its southwestern shore[54-58]; (7) the El-Max (*ELMAX*) site located on the western side of the city of Alexandria, Egypt (31°9'31.20"N, 29°50'28.20"E), which is the most contaminated seashore in the Alexandria region and exceeds legal environmental pollution limits for heavy metals, poly-aromatic hydrocarbons (PAH), and crude oil-derived pollutants[59]; (8) the Gulf of Aqaba (*AQ*) along the Jordanian coast at the northern end of the Red Sea (30°22'42"N, 25°24'57"E), which is the northernmost tropical sea ecosystem and contains a major oil terminal moving between 20-30 million tons yearly characterized by frequent pollution with accidental oil spills at the oil terminal as well as spills (with high sulfur concentrations) during loading and unloading of ships at the industrial jetty[60-63]. When necessary, the samples were named based on the code 'MGS', which refers to MetaGenome Source, followed by a short name indicating the origin of the sample, as follows: MGS-HAV (Haven tanker at the Gulf of Genoa); MGS-MES (the harbor of Messina); MGS-PRI (the harbor of Priolo Gargallo); MGS-MCh (the lagoon of Mar Chica); MGS-BIZ (the Bizerte lagoon); MGS-ELMAX (El-Max site); MGS-ELF (Elefsina site); and MGS-AQ (Gulf of Aqaba).

Sample collection, environmental measurements, and nucleic acid extraction. Sediment site duplicates (5.0 kg) were collected at a water depth of 1.0 to 78.0 m (October 2011) by scuba. Analytical procedures, in triplicates per each of the duplicates, are as follows. Temperature, salinity, pH, redox potentials and dissolved oxygen were measured immediately by a portable multiparametric probe analyser (WP 600 Series Meters Eutech instruments Pte Ltd Singapore). Determination of oxygen concentration was carried out using the Winkler method with an automatic endpoint detection burette 716 DNS Titrino (Metrohm AG, Herisau, Switzerland). Samples for measurements of NO_3^-, NO_2^- and PO_4^{3-} and nutrient concentrations were stored at −20 °C and were determined later in triplicate in the laboratory using a "SEAL AutoAnalyzer Quaatro" following classical methods[64] with slight modifications adapted for sediments. Briefly, 1 kg of melted sediments were placed in the PVC tube of 15 cm diameter and 50 cm length. Holes of 0.1 cm diameter were drilled at the bottom of the tube and sealed with a rubber tape before filling. For the retrieval of porewater the tapes covering the sampling holes were cut open with a paper knife. A MicroRhizon sampler (Rhizosphere Research Products, Wageningen, Netherlands) of 2 cm length and 1 mm diameter connected to a 1 mL syringe was inserted horizontally and porewater were drawn out gently. Conductivity calibration was carried out with a KCl 0.01 mol/L control solution. Reference solutions with pH values of 7.0 and 9.0 were employed for pH

meter. Ammonium was determined using the indophenol blue technique (IOC, 1983). The dissolved organic carbon content was determined by the dichromate wet oxidation method[65,66]; total organic matter content was calculated by multiplying the values of the organic carbon by 1.8. The amount of total extracted and resolved hydrocarbons (TERHC), was determined as follows. Briefly, TERCH were extracted from sediments following the 3550C EPA (Environmental Protection Agency) procedure. Briefly, 500 mL mixture of CH_2Cl_2:CH_3COCH_3 (1:1, vol/vol) was added to 1,000 x *g* of dry sediments, sonicated for 2 minutes in ultrasound bath (Branson 1200 Ultrasonic Cleaner, Branson USA). Samples were further shaked at 150 rpm for 30 minutes, centrifuged for 10 minutes at 5,000 x *g* and the supernatant was passed through a ceramic column filled with anhydrous Na_2SO_4 (Sigma-Aldrich, Milan). Same treatment of sediments was repeated with 500 mL of CH_2Cl_2 and the obtained solvents were combined and volatilized to the dryness. Residues were re-suspended in CH_2Cl_2 prior the gas chromatography (GC) analysis. All measures were performed using a Master GC DANI Instruments (Development Analytical Instruments), equipped with SSL injector and FID detector. Sample (1 μL) was injected in splitless mode at 330 °C. The analytical column was a Restek Rxi-5 Sil MS with Integra-Guard, 30m x 0.25 mm (ID x 0.25 μm film thickness). The helium carrier gas was maintained at a constant flow of 1.5 mL/min. TERCH were calculated using the mean response factors of *n*-alkanes, i.e. individual *n*-alkane concentrations from *n*-C_{15} to *n*-C_{40}, pristane and phytane were calculated for each sample. The amount of analyzed TERCH was expressed as ppm (part per million) or mg/kg.

Nucleic acid extraction was performed directly from 10 g of sediment duplicate samples using the PowerMaxSoil® DNA Isolation Kit (MoBio, CA, USA) according to supplier's recommendations. Once extracted, DNA concentration was measured by using PicoGreen Quantification Reagent (Invitrogen, ORE, USA), and equal amount of both (1 μg each) was mixed for further analysis (SSU rRNA hypervariable tag analysis and DNA sequencing).

SSU rRNA hypervariable tag analysis. Pyrotag assays were performed using universal-bacterial primers targeting the variable regions of the 16S rRNA, V1-V3 (27 F mod 5' – AGRGTTTGATCMTGGCTCAG – 3'; 519 R mod bio 5' – GTNTTACNGCGGCKGCTG – 3'), amplifying a fragment of approximately 400 bp. The amplified 16S rRNA regions contain sufficient nucleotide variability to enable the identification of bacterial species[67,68]. Multiplex identifiers (MIDs) specific to each sample were used: TCCAGTAC for HAV, TCCAGGTG for PRI, TCATCTCC for BIZ, TCATGGTT for ELMAX, TCATTGTT for MCh, TCCACGTG for MES, TCAGTAAG for AQ, and TAGGATGA for ELF.

PCR reactions were performed in a final volume of 50 μL with 40 ng of sample DNA, 0.3 μmol/L of each primers, 1× PCR Buffer with 1.5 mmol/L of $MgCl_2$, 0.2 mmol/L of each dNTP

and 1U of HotStarTaq Plus Master Mix Kit (Qiagen, Valencia, CA, USA). The PCR cycling procedure was as follows: 94 °C for 3 min followed by 28 cycles at 94 °C for 30 sec, 53 °C for 40 sec and 72 °C for 1min; a final elongation at 72 °C for 5 min was performed. After PCRs, all amplicons were purified using Agencourt Ampure beads (Agencourt Bioscience Corporation, MA, USA) and an equal amount was sequenced using Roche FLX 454 titanium. PCR and next-generation 454 pyrosequencing were performed at MR DNA laboratories (Shallowater, TX – U.S.).

A first-quality filter was applied to remove sequences shorter than 300 bp, longer than 500 bp, or with an average quality score (Phred score) of less than 30 (0.1% per-base error probability). The high-quality 16S rRNA gene sequences obtained by 454 pyrosequencing were then analyzed using QIIME[69] as follows: the sequences were clustered into operational taxonomic units (OTUs) based on a threshold of 97% for sequence identity using UCLUST[70]. The combination of applied quality filtering and clustering threshold at 97% guarantee that the influence of erroneous reads is minimized[71]. A representative sequence from each OTU was selected and aligned to Greengenes (13_8 release; http://greengenes.lbl.gov/) using PyNast[72]. After OTU clustering, Chimeraslayer was used in order to remove all chimeric OTUs from the dataset. Sequence identification was, then, conducted using Ribosomal Database Project classifiers with default parameters[72]. For each sample, the Shannon index and rarefaction curves of the observed species were estimated to analyze the species sampling coverage. The OTU diversity within and between samples (alpha and beta diversity) was estimated using QIIME (http://qiime.org/) workflow scripts. The QIIME workflow, including the commands used for bacterial SSU rRNA hypervariable tag analysis, are indicated below:

	**QIIME command**
**#seq filter and sample assign**	- split_libraries.py -m *.mapping.txt -f *.fasta -q *.qual -o split_library_output -r -l 300 -L 500 -s 30 -b hamming_8
**#otu table creation**	- pick_de_novo_otus.py -i split_library_output/seqs.fna -o otus -a -O 4
**#Inside the dir otus, remove the file *.tree**	
**#chimera removal**	- identify_chimeric_seqs.py -i pynast_aligned_seqs/seqs_rep_set_aligned.fasta -a /home/qiime_software/chimeraslayer-4.29.2010-release/RESOURCES/rRNA16S.gold.NAST_ALIGNED.fasta -m ChimeraSlayer -o otus/chimeric_seqs.txt<br>- filter_fasta.py -f otus/pynast_aligned_seqs/seqs_rep_set_aligned.fasta -o otus/pynast_aligned_seqs/non_chimeric_seqs_rep_set_aligned.fasta -s otus/chimeric_seqs.txt -n<br>- filter_alignment.py -i otus/pynast_aligned_seqs/non_chimeric_rep_set_aligned.fasta -m /home/qiime_software/lanemask_in_1s_and_0s -o otus/pynast_aligned_seqs/<br>- make_phylogeny.py -i

	otus/pynast_aligned_seqs/non_chimeric_rep_set_aligned_pfiltered.fasta -o otus/rep_phylo.tre<br>- make_otu_table.py -i otus/uclust_picked_otus/seqs_otus.txt -o otus/non_chimeric_otu_table.biom -e otus/chimeric_seqs.txt -t otus/rdp_assigned_taxonomy/seqs_rep_set_tax_assignments.txt
**#stat**	- biom summarize-table -i otus/otu_table.biom -o otus/otu_table_summary.txt
**#cleaning the OTU table (removing OTUs contaminants)**	- biom convert -i otus/non_chimeric_otu_table.biom -o otus/table.from_biom.txt -b #convert the biom to txt<br>- biom convert -i otus/non_chimeric_otu_table.biom -o otus/table.from_biom_w_taxonomy.txt -b --header-key taxonomy<br>- copy table.txt to cleaning directory and use the cleaning script<br>- filter_otus_from_otu_table.py -i otu/non_chimeric_otu_table.biom -o non_chimeric_otu_table_cleaned.biom -e non_chimeric_otu_table_cleaned.txt
**#taxa summary**	- summarize_taxa_through_plots.py -i otus/non_chimeric_otu_table_cleaned.biom -o wf_taxa_summary -m Fasting_Map.txt
**#alpha div**	- echo "alpha_diversity:metrics shannon,PD_whole_tree,chao1,observed_species" > alpha_params.txt<br>- alpha_rarefaction.py -i otus/non_chimeric_otu_table_cleaned.biom -m mapping_file.txt -o wf_arare/ -p alpha_params.txt -t otus/rep_phylo.tre
**#beta div**	- beta_diversity_through_plots.py -i otus/non_chimeric_otu_table_cleaned.biom -m Fasting_Map.txt -o wf_bdiv_even146/ -t otus/rep_phylo.tre -e cutoff_value<br>- make_2d_plots.py -i (un)weighted_unifrac_pc.txt -m Fasting_Map.txt (-e flag [cutoff_value] is 6000)

Beta diversity was assessed after construction of weighted and unweighted Unifrac distances[73] with weighted Unifrac accounting for differences in the relative abundance of microbial community members. To remove noise from the data, including potential rare contaminants, we removed OTUs that did not meet the criterion of being present in at least 0.05% of the total number of reads using an in-house script, as follows:

```
# Remove OTUs present under a fixed threshold

# python clean_OTU_mod.py [otu_table] [threshold]

import sys



inF=open(sys.argv[1].strip(),"r")

thr=int(sys.argv[2].strip())

assert (thr > 0); 'you should enter a positive number'
```

```
outname="%s_cleaned" %(sys.argv[1].strip())
out=open(outname,"w")

for oline in inF.xreadlines():
        if oline[0]=="#":
                out.write(oline)
        else:
                line=oline.strip().split("\t")
                line.pop(0)
                tot=0.0
                for a in line:
                        tot+=float(a)
                if tot<float(thr): #this number is the limit of the OUTs number to keep
                        out.write(oline)

inF.close()
out.close()
```

The Shannon diversity index was calculated by PAST software[72]. Library coverage was calculated for each library using the equation $C = [1 - (n1/N)] \times 100$, where n1 is the number of singleton OTUs and N is the total number of reads in the library.

DNA sequencing, assembly, and gene calling. Sequencing of AQ was performed with a Roche 454 GS FLX Ti sequencer (454 Life Sciences, Branford, CT, USA) at Lifesequencing S.L. (Valencia, Spain) in a single picotiter plate. Assembly was performed using a Roche Newbler assembler v. 2.5.3 using the default parameters, and potential protein-coding genes were predicted and annotated as described previously[75]. All other DNA samples were sequenced by pair-end sequencing (Illumina Hiseq 2000) at Beijing Genomics Institute (BGI; China); software

MetaGeneMark (version 2.10, default parameters) was used to predict potential protein-coding genes based on the assembly results.

For Illumina Hiseq 2000 sequencing and data processing, the DNA samples were sequenced following standard pipelines in Illumina platform. Data filtration was done by in-house scripts, listed as follows:

(1) removing reads with 3 N bases removing reads contaminated by adapter(15 bases overlapped by reads and adapter)

(2)Remove reads with 20 bp low quality (20) bases

(3)Remove duplication contamination.

The removal reads process is simultaneously read1 and read2 operation, finally obtained can be used for subsequent analysis to quality data (Clean Data)[76].

For assembly for Illumina Hiseq 2000 sequences, SOAPdenovo (Version 1.0, http://soap.genomics.org.cn/soapdenovo.html) was used to assemble filtered data and assembly results with the best N_{50} contig length were optimized by in-house scripts previously described[77].

The data statistics, assembly results data, number of open reading frames (ORF) and number of ORF with assigned function for each of set of sequences are shown in Supplementary Table S6.

Biodegradation network reconstruction

Data for the *in silico* degradation network reconstruction were based in three different sequence datasets, resulting in three different reconstructions. The first dataset was built according with the similarity comparison (score > 45; e-value $< 10e^{-3}$) between the gene sequences from the metagenomes of the samples and the sequences from AromaDeg database[24,27]. The second dataset was based on the results from the 16S rRNA phylogenetic affiliations, building a putative metagenome for each of the samples based on the detected taxons. For that, we downloaded the genome proteins (from the NCBI website) belonging to one of the closest species inside these taxons, and query sequences that matches a given protein family of the AromaDeg[24,27] were selected. The last dataset was the result of joining both prior data sets, metagenome-based and 16S rRNA-based data sets. These data sets were used to create a nodes Table (Supplementary Table S5) on the basis of which we develop a network reconstruction under *R* language[78,79], that is described below.

Step 1: Creating the nodes Table

Each connection in the network represents a step in the degradation pathway (a degradation reaction), connecting a product with its substrate (nodes), which is assigned to a gene encoding a

catabolic enzyme. An in house code is used to identify enzymes and compounds in the Table, formed by two numbers. The first number indicates their location according to the whole degradation pathway: 01 when is an initial or peripheral step, 02 when is a first stage intermediate product, and 03 when is an intermediate product close connected with the tricarboxylic acid (TCA) acid/pathway. The second is an arbitrary number which identify each specific element (catabolic gene/enzyme and reaction substrate or product). The follow scheme (Scheme 1) summarized the codes:

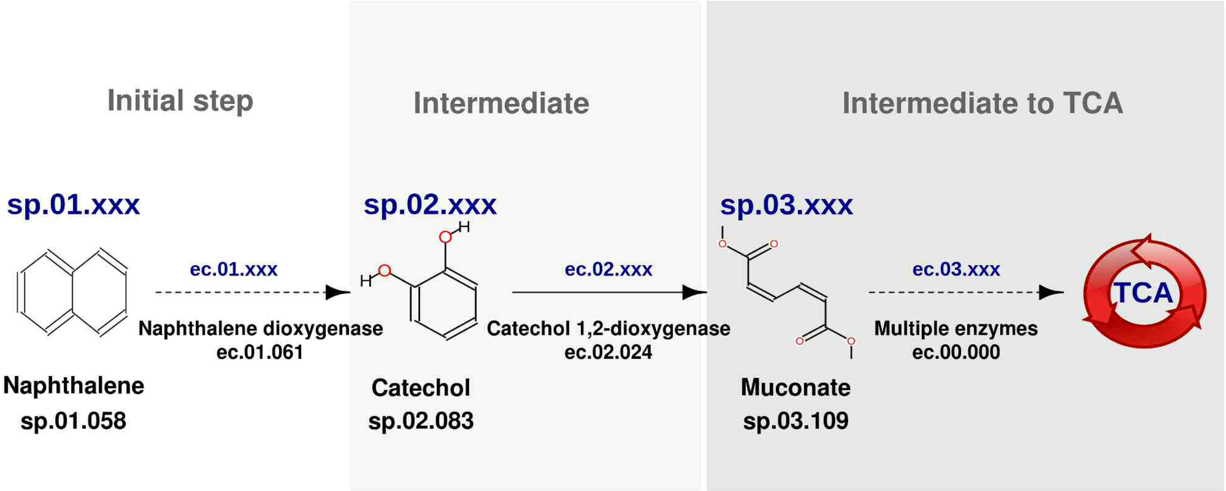

Scheme 1: Description of the codes assignation to catabolic genes/enzymes and compounds in the network. General pattern for the assignation is highlighted in blue, while in black we described an example with the naphthalene degradation pathway. Code ec.00.000 is assigned to existing reaction/s without any representative sequence in AromaDeg database[24,27]. Single step reactions are represented by solid lines, while transformations involving multiple reactions are represented by dashed lines.

Relative abundance for each type of gene encoding catabolic enzymes found for each sample (according to the list of detected gene sequences encoding enzymes potentially involved in degradation) is used to set up the nodes Table (Supplementary Table S5A), resulting in a list of weights that specifies the size of the connections in each step of the network for each sample, as exemplified below.

EC code	Substrate code	Product code	MGS-HAV	MGS-PRI	MGS-MES	MGS-MCH	MGS-BIZ	MGS-ELMAX	MGS-AQ
ec.01.061	sp.01.058	sp.01.003	0,024	0,000	0,052	0,013	0,144	0,072	0,067
ec.01.006	sp.01.003	sp.01.014	0,000	0,000	0,000	0,000	0,000	0,000	0,000
ec.00.000	sp.01.014	sp.01.067	0,000	0,000	0,000	0,000	0,000	0,000	0,000
ec.01.023	sp.01.067	sp.02.083	0,000	0,000	0,002	0,000	0,007	0,000	0,000
ec.02.024	sp.02.083	sp.03.109	0,000	0,000	0,015	0,007	0,004	0,008	0,011
ec.02.017	sp.02.083	sp.03.124	0,024	0,000	0,062	0,063	0,155	0,095	0,107
ec.00.000	sp.03.109	sp.04.000	0,000	0,000	0,000	0,000	0,000	0,000	0,000

Example of a part of the nodes Table. Codes for the genes/enzymes, substrates and products (intermediates) are shown in grey. Weights (relative abundance of catabolic genes) for each reaction in each sample are shown in blue. For complete set of data see Supplementary Table S5A.

Step 2: Setting up the nodes of the network

Network structure is set up under the programming language *R*[78,79], using the functions provided by the package *igraph* and the information given in the nodes Table. The process starts calling the functions of the package, opening the Table under the *R* environment and creating a new graph object with the substrates/products of the Table like nodes:

```
> library(igraph)
> edgelist <-read.table("NodesTable.txt",
+    header=TRUE,dec=",",sep="\t",check.names=FALSE)
> g <-graph.empty(directed=TRUE)
> u <-unique(c(as.character(edgelist[,2]),
+                 as.character(edgelist[,3])))
>  g<-add.vertices(g,length(u),name=u,
+                     size=size,degree=degree,dist=dist)
```

After creating a new graph with `graph.empty`, all the substrate/product names are listed in a value with unique and added as nodes of the new graph with `add.vertices`, where some attributes like the size of the node or the position for the labels can be set up using values like *size*, *degree* or *dist*.

Step 3: Adding the connections between nodes to the network

There are two different types of connections, those with 0 abundance in all samples (empty connections) and the connections with at least one sample with an abundance > 0 (positive connections). We make this difference in order to set up independently the drawing attributes of both types of connections, like the type and curve of the line in the arrows. See scheme 2 below.

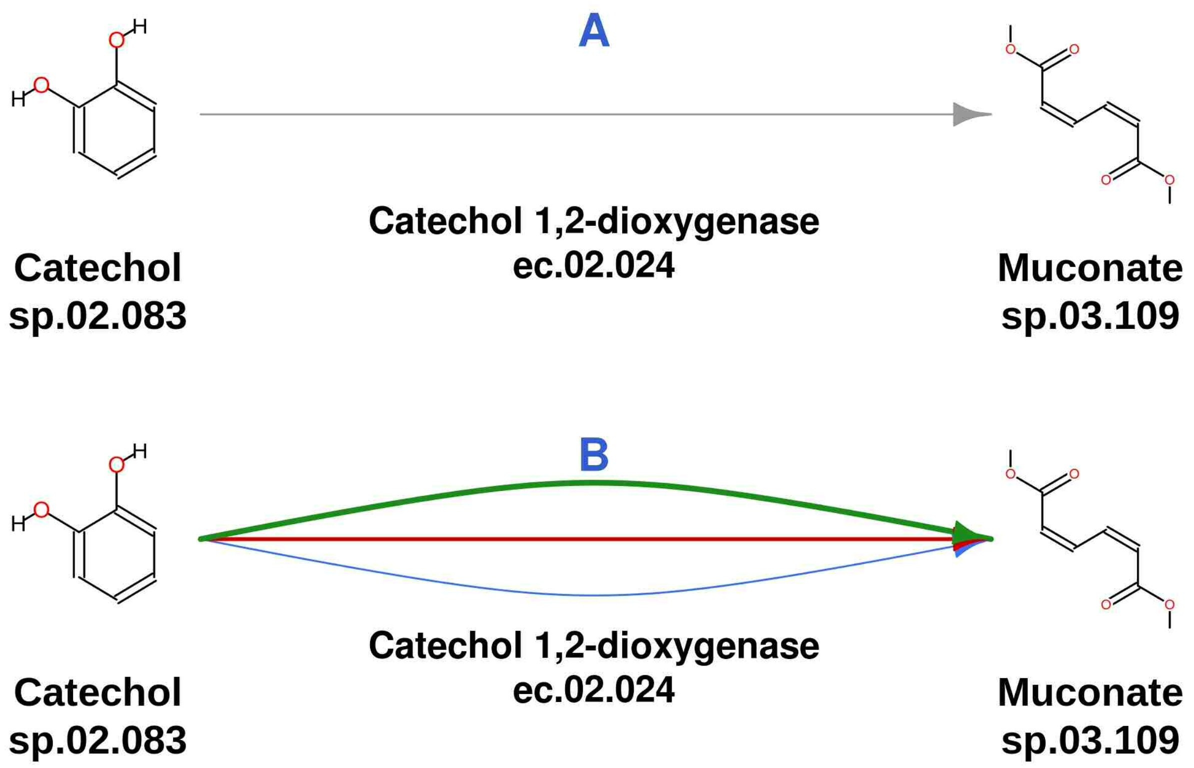

Scheme 2: Description of the two different types of connections. A, an empty connection with 0 abundance in all the samples. B, a positive connection with abundance >0 in three different samples (represented in green, red and blue line), with the size of the line according to the relative abundance value of catabolic gene for each sample.

Empty connections are added first. A loop checking the data for each sample is needed:

```
> for(i in 1:nrow(edgelist)){
+    if (sum(edgelist[i,4:ncol(edgelist)])==0){
+              g<- add.edges(g,rbind(edgelist[i,2],edgelist[i,3]),
+                           attr=list(color="grey60",curve=0,
+                              name=as.character(edgelist[i,1])))
+    }
+}
```

Connections are introduced in the graph with the function `add.edges`. Calculating the total abundance in each network step (row) from the nodes Table, one can see whether the abundance is 0 in all the samples (from the fourth to the final column in the Table [Supplementary Table S5A]); in this case a simple connection is added to the graph with a grey arrow. Another loop is run to add the positive connections (abundance >0 at least in one sample):

```
> curve<-0
> for(i in 4:ncol(edgelist)){
+           from<-NA
+           to<-NA
+           weights<-NA
+           name<-NA
+           newfrom<-na.omit(from)
+           newto<-na.omit(to)
+           weights<-na.omit(weights)
+           name<-na.omit(name)

+      for(j in 1:nrow(edgelist)){
+           if (edgelist[j,i] > 0){
+              from<-append(from,
+                              as.character(edgelist[j,2]),
+                              after=length(from))

+             to<-append(to,
+                           as.character(edgelist[j,3]),
+                           after=length(to))
+             weights<-append(weights,
+                              edgelist[j,i],
+                              after=length(weights))

+             name<-append(name,
+                           as.character(edgelist[j,1]),
+                           after=length(name))
+         }
+      }
+    g<- add.edges(g,rbind(from,to),
+                    attr=list(weight=weights,
+                             color=color,curve=curve),name=name)
+
+           if (curve%%2==0){
```

```
+                   curve<-abs(curve)
+               }
+               else{
+                   curve<- -curve
+               }
+
+               if (curve<0){
+                  curve<-curve
+               }
+               else{
+                  curve<-abs(curve)+0.2
+               }
+
+}
```

In this case (abundance >0 at least in one sample) the loop is more complicated. In the first part, empty vectors for each sample (from the fourth to the last column in the Table [Supplementary Table S5A]) are created to save (using the function `append`) the different attributes of the connections in each case (name, weight and nodes of the connections). The connections for each sample are added to the graph at the final of the loop again with `add.edges`. The attribute *curve* is configured before running this step and is changed at the end of the loop to set the curve for the next sample.

Reason for running two independent `for` loops, checking twice the whole table, is simple. Checking empty connections needs to look over the table row by row, like in the first loop, but checking the positive connections needs to look over the table column by column (sample by sample), like in the second loop.

Note that the line for empty connections is drawn in grey color, which means that abundance in this case is 0, and the width of the line is not representing any percentage of gene presence. Also, these connections can represent a single step in the pathway (straight line) or multiple reactions (dashed line).

Step 4: Setting up the coordinates of the nodes in the network

Coordinates of the nodes determine the position of each node (substrate/product) in the final draw of the network. This coordinates can be set manually, in order to obtain a customized layout for the network, saved in a file and use this file when is needed to draw a new network, without a new manual set up:

```
> p <- tkplot(g)
> Coords <- tkplot.getcoords(p)
> write.table(Coords,”Coords.txt”,row.names=FALSE,col.names=FALSE)
> Coords<- matrix(scan(“Coords.txt”),nc=2,byrow=TRUE)
```

Function `tkplot` displays a new interactive screen where we can point each node in the desired position and then save the coordinates in a value with `tkplot.getcoords`. Using `write.table` is possible to print the value with the coordinates in an output file, and read it again using `matrix` and `scan`.

Step 5: Drawing the network

Network can be drawn using the coordinates and the configuration in the prior steps:

```
>  jpeg("Network.jpg",width=5796,height=3561,
+    res=300,quality=100,units="px")

> par(mar=c(0,0,0,0),xpd=TRUE)
>  plot.igraph(g,
+            layout=Coords,
+    vertex.shape=shape,
+    vertex.size=size1,
+    vertex.size2=size2,
+    vertex.size2=size2,
+    vertex.label=labels,
+    vertex.label.dist=V(g)$dist,
+    vertex.label.degree=V(g)$degree,
+    vertex.label.dist=V(g)$dist,
```

```
+     vertex.label.degree=V(g)$degree,
+     edge.width=ifelse(E(g)$weight<=0.01,1,
+                     ifelse(E(g)$weight>0.10,10,E(g)$weight*100)),
+     edge.lty=lty
+     )

+ dev.off()
```

Functions `jpeg` and `dev.off` are used to save the plot in a jpeg file. The main function to draw the network is `plot.igraph`, using the coordinates saved before as layout, and the parameters provided when the vertex were added to the graph object to set up the different options of the function. Other options can be modified using vector objects with values for the different vertex/connections. Abundances for each node in each sample are used as the width of the connections (saved as connection *weight* in the step 2) but are adapted, with a conditional loop (`ifelse`), to make them fit in the plot. Herein, an abundance of 0.01 is equal 1 in the ***edge.width*** parameter, so this value will be the abundance multiply by 100 (0.02 is equal to 2, 0.05 is equal to 5). When the ***edge.width*** value is higher than 10 (for abundances > 0.1) the value is set in 10, and if the value is lower than 1 (abundances <0.01) the width is set in 1. For empty connections abundance is set as 0, but in the network will be drawn with a size of 1 as is configured in the parameter *edge.width.* This is because a size >0 must be indicated in the script in order to draw a visible connection; however a grey color is used in these connections to specify the absence of abundance in these reaction/s.

Target metabolomics for experimental validations of predicted biodegradation capacities

The ability of each of the microbial communities to grow on a mix of distinct pollutants as the sole source of carbon and energy was evaluated in 1 l Erlenmeyer flasks containing 100 mL of ONR7a[80] minimal medium, supplemented with 10 mM total substrate mix. The sediment samples used for enrichment cultures were exactly the same than those used for environmental measurements and nucleic acid extractions; note that in this case, the sediments were stored at −20 °C prior to use. The following pollutants, all from Fluka-Aldrich-Sigma Chemical Co. (St. Louis, MO, USA), were used: naphthalene, tetradecane, benzoate, 4-chlorobenzoate, 3-nitrobenzoate, 4-hydroxybenzoate, phthalate, isophthalate, terephthalate, anthracene, 2,3-dihydroxybiphenyl, 4-hydroxyphenylpyruvate, 3,4-phenoxybenzoate, carbazole, phenol, 2,4,5-trihydroxytoluene and gallate. One liter Erlenmeyer flasks were filled with 100 mL of sediment,

sterilized, and spiked with 10 mL of sterile-filtered Arabian light crude oil. The sediment sample was used to inoculate 300 mL of modified ONR7a medium (omitting ammonium chloride and disodium hydrogen phosphate). We used an amount of sediment corresponding to approximately $2.0e^{+05}$ cells per g, so the same amount of bacterial cells was used in each of the microcosm experiments. To calculate cell numbers in the sediments, cell counts were performed over fixed (4% formaldehyde for 4 h at 4°C), 4',6-diamidino-2-phenylindole (DAPI)-stained samples immobilized onto 8-well teflon printed slides by manual [Cell Counter plugin incorporated in ImageJ v1.47 (http://imagej.nih.gov/ij/)[81]] or automated counting of single color images (ImageJ v1.47), depending on the sample's characteristics. To achieve statistical significance, 50 fields were examined per sample. Cell numbers were obtained by referencing the counts to the screened area and the amount of sediment used

We added the substrate mix to give a final concentration of 10 mM (from a 100 mM stock solution each in methanol). A total of 5 mM NH_4Cl and 0.5 mM Na_2HPO_4 to a molar N:P ratio of approx. 10:1, were added. Enrichment cultures, performed in triplicates per each of the duplicate sediments, were incubated at 250 rpm for up to 3 weeks. Samples were taken after incubation for 1 day (time zero in our assay) and 3 weeks. Oxygen and temperature concentrations were maintained at values corresponding to environmental values (see the Supplementary Table S1), except for sediment sample from Priolo Gargallo for which 1.0 mg L^{-1} O_2 was used; note that in this case, the sediment sample was anaerobic and, in order to detect aerobic degradation products, low O_2 concentration was added for the enrichments. Two control experiments (in triplicates) were used under each of the conditions tested: i) cultures without the addition of sediments but with chemicals; ii) cultures plus sediments but without the addition of chemicals. The extent of degradation and transformation in test and control samples was quantified in a solution containing 1 mL of the culture medium (previously separated by centrifugation at 13,000 g for 5 min) and 1 mL of a methanol solution prepared as follows. Briefly, microbial cells were harvested from the enrichment by centrifugation at 13,000 g for 5 min; the metabolites were then extracted by adding 1.2 mL of cold (-80°C) methanol as previously described[82]. The presence of each of the 17 pollutant molecules as well as key 9 degradation products produced after their degradation, namely, catechol, chlorocatechol, salicylate, muconate, gentisate, protocatechuate, homogentisate, myristate and homoprotocatechuate, was determined by target analysis by gas chromatography-mass spectrometry (GC-Q-MS) and liquid chromatography-mass spectrometry (LC-QTOF-MS) using the following reagents: O-methoxyamine hydrochloride (Sigma-Aldrich - Taufkirchen, Germany), 15 mg/mL in pyridine (Silylation grade - Taufkirchen, Germany); N,O-Bis(trimethylsilyl)trifluoroacetamide (BSTFA) with 1% trimethylchlorosilane (TMCS; Pierce Chemical Co, Rockford, IL, USA); C18:0 methyl ester (Sigma-Aldrich - Taufkirchen, Germany) in

heptane (GC-MS grade – Sigma-Aldrich - Taufkirchen, Germany); and isopropanol (HLPC-MS grade – Sigma-Aldrich - Taufkirchen, Germany), in addition to the appropriate standards. Napthalene, tetradecane and toluene were added to the assay, but the analytic method used did not permit the quantification of these compounds, so their relative concentrations during the assay could not be determined; however, the identification and quantification of degradation intermediates could demonstrate the capacity of microbial communities to degrade them.

The presence (no degradation), absence (total degradation) and abundance (partial degradation) level of mass signatures of all tested pollutants and key degradation intermediates can be linked to the presence of 21 key genes encoding catabolic enzymes involved either in their degradation (in case of initial pollutants) or their production (in case of intermediates). The links between the presence or absence of substrate pollutants and intermediate (in a degradation reaction), and a gene encoding a catabolic enzyme assigned to the degradation reactions where both chemical species participates, were stablished on the basis of AromaDeg[24] and nodes described in Supplementary Table S5A. Links are summarizes in the Scheme 3 as follows:

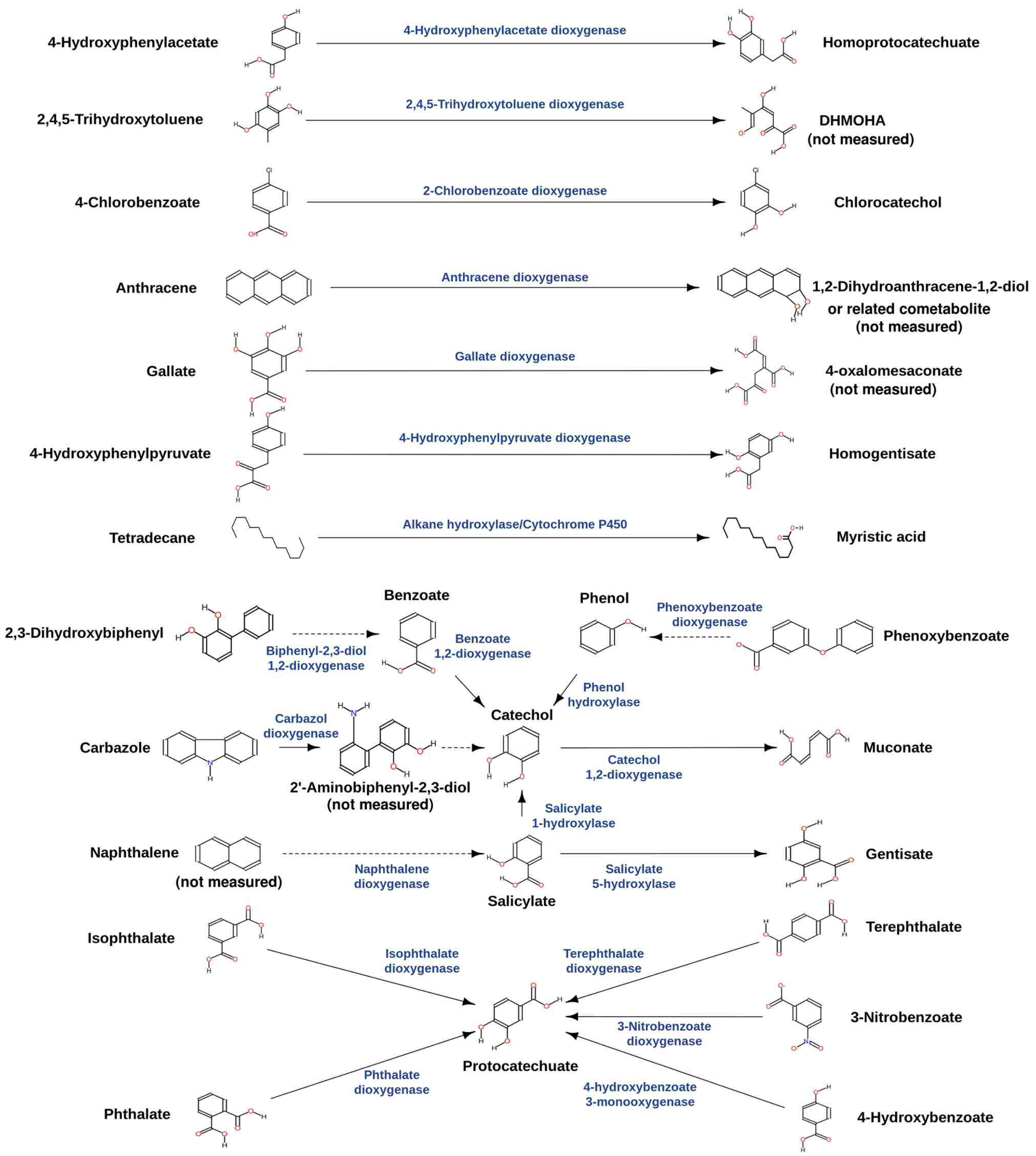

Scheme 3. Association between biodegradation reactions and genes encoding catabolic enzymes. Abbreviations as follows: DHMOHA, 2,4-Dihydroxy-5-methyl-6-oxohexa-2,4-dienoate. Note, that "not measured" indicates metabolites which were not measured by target metabolomics.

We are aware about the fact that in the enrichment assays we used artificial incubations and conditions to determine the catabolic potential of the microbial communities from sediments samples herein investigated. Such enrichment cultures may have a number of limitations to predict the actual catabolic potential or microbial communities as might be expected in the field sediment. However, here we would like to point to the fact that the reason of these experiments

are not to identify the most active genes encoding catabolic enzymes or the abundance level of them in each of the microbial communities after enrichments with particular substrates, but rather to demonstrate the capacity to degrade distinct substrates. Although, the abundance level in field sediments might be different to the one in the enrichments cultures[29], it is highly likely that the presence of a catabolic activity in an enrichment culture implies that such activity also exist, albeit at different level, in the natural environment.

Target analysis by GC-Q-MS: Samples for GC-MS analysis were prepared from 100 µL volumes of the methanol extracts. Blanks reflecting the matrices of the samples were prepared and treated in the same manner as the samples. Standards were prepared at a concentration of 100 ppm. All samples were evaporated to dryness using a Speedvac Concentrator (Thermo Fisher Scientific Inc., Waltham, MA) and derived using a two-stage process of methoximation and silylation. For methoxymation, 10 µL of O-methoxyamine hydrochloride (15 mg/mL) in pyridine was added to each of the samples, which were then subjected to three cycles of vortex mixing and ultra-sonication and kept in the dark at room temperature for 16 h. For silylation, 10 µL of BSTFA with 1% TMCS was added to the samples, which were then subjected to three cycles of vortex mixing and ultra-sonication before incubation at 70 °C for 1 h. Finally, 100 µL of 10 ppm C18:0 methyl stearate in heptane (internal standard) was added to each sample, and all samples were vortex mixed for 2 min. The analytical run started with the injection of C18 (10 ppm), followed by three blanks to equilibrate the column. Subsequently, samples were analyzed in random order, followed by the standards. The run terminated with the injection of the final blank. The GC-MS system (Agilent Technologies 7890A) consisted of an autosampler (Agilent Technologies 7693) and an inert mass selective detector (MSD) with Quadrupole (Agilent Technologies 5975). The derived samples were injected in 2 µL volumes onto a GC-Column DB5-MS (30 m length, 0.25 mm i.d., 0.25 µm film 95% dimethyl/5% diphenyl polysiloxane) with a pre-column (10 m J&W integrated with Agilent 122-5532G). The helium carrier gas flow rate was set at 1 mL/min, and the injector temperature was set at 250°C. The split ratio was 1:10 flowing into a Restek 20782 deactivated glass-wool split liner. The temperature gradient was programmed as follows: the initial oven temperature was set at 60°C (held for 1 min) and then increased to 325°C at the rate of 10°C/min. Finally, a cool-down period was applied for 10 min before the subsequent injection. The total analysis time for each sample was 37.5 min. The detector transfer line, the filament source, and the quadrupole temperatures were set at 290°C, 230°C, and 150°C, respectively. The electron ionization (EI) source was operated at 70 eV. The mass spectrometer was operated in scan mode over a mass range of m/z 50-600 at a rate of 2 spectra/s. Peak detection and spectra processing were obtained using Agilent ChemStation Software (G1701EA E.02.00.493, Agilent). Compound identification was performed using the NIST 08 Library

(National Institute of Standards and Technology, U.S. Department of Commerce) with ChemStation software (G1701EA E.02.00.493, Agilent). As soon as they were properly characterized in the chromatograms of the standards (retention time and spectrum), a target analysis method was created in the ChemStation software (G1701EA E.02.00.493, Agilent) to identify and integrate the corresponding peaks in the chromatograms of the samples.

Target analysis by LC-QTOF-MS: Samples for LC-MS analysis were prepared by filtering the methanol extracts using 0.2 µm nylon syringe filters (Thermo Scientific, Rockwood, USA). The analytical run began with the analysis of ten QCs, followed by the samples in random order; a QC sample was injected in between blocks of four samples until the end of the run. Finally, the corresponding standards (100 ppm) were analyzed. All vials were maintained at 4°C throughout the run. Each metabolic fingerprint was achieved using a liquid chromatography system consisting of a degasser, binary pump, and autosampler (1290 infinity, Agilent). A total of 0.5 µL was applied to a reverse-phase column (Zorbax Extend C_{18} 50 × 2.1 mm, 3 µm; Agilent), which was maintained at 60°C during the analysis. The system was operated in positive and negative ion mode at a flow rate of 0.6 mL/min with solvent A (water with 0.1% formic acid) and solvent B (acetonitrile with 0.1% formic acid). The gradient was 5% B (0-1 min), 5 to 80% B (1-7 min), 80 to 100% B (7-11.5 min), and 100 to 5% B (11.5-12 min), followed by reequilibration at 5% B for 3 min (15 min of total analysis time). Data were collected in positive and negative ESI mode in separate runs on a QTOF (Agilent 6550 iFunnel). Both ion modes were operated in full scan mode (*m/z* 50 to 1,000 in positive and *m/z* 50 to 1,100 in negative ion mode). For each mode, the capillary voltage was 3,000 V, the scan rate was 1.0 spectra/s, the gas temperature was 250°C, the drying gas flow was 12 L/min, and the nebulizer was 52 psi. The MS-TOF parameters for positive ion mode were as follows: fragmentor 175 V, skimmer 65 V, and octopole radio frequency voltage (OCT RF Vpp) 750 V. The same MS-TOF parameters were used in negative ion mode, except the fragmentor, which was set to 250 V. Two reference masses were used for each mode: *m/z* 121.0509 ($[C_5H_4N_4+H]^+$) and *m/z* 922.0098 ($[C_{18}H_{18}O_6N_3P_3F_{24}+H]^+$) during positive analysis and *m/z* 112.9855 ($[C_2O_2F_3-H]^-$) and *m/z* 1033.9881 ($[C_{18}H_{18}O_6N_3P_3F_{24}+TFA-H]^-$) during negative analysis. The reference masses were continuously infused into the system to permit constant mass correction[83]. Compound identification and peak integration were performed using Mass Hunter Qualitative Analysis (B.05.00, Agilent). Following their identification in the chromatograms of the standards (retention time and spectrum), the molecular ion of each compound that was not previously identified in GC-MS was searched for in the chromatograms of the samples.

Metabolomic fingerprint analysis of sediment samples. The metabolites were extracted in triplicates from sediment samples as follows. In a 100 mL Erlenmeyer flask, 5 g of sediments were mixed with 10 mL of cold (-80°C) HPLC-grade methanol (MeOH). The samples were sonicated for 120 sec (at 15 W) on an ice water bath. This protocol was repeated 4 times, and the samples were kept on ice for at least 2 min between each step. After sonication, the supernatant was removed by centrifugation at 10,000 *g* for 30 min at 4°C, and the supernatant was stored in 20-mL penicillin vials at -80°C. Methanol extracts were filtered using 0.2 µm nylon syringe filters (Thermo Scientific, Rockwood, USA) and analyzed by LC-QTOF-MS as described above.

Supplementary References

32. Hedlund, B. P. & Staley, J. T. Isolation and characterization of *Pseudoalteromonas* strains with divergent polycyclic aromatic hydrocarbon catabolic properties. *Environ. Microbiol.* **8,** 178-182 (2006).
33. Yakimov, M. M., Timmis, K. N. & Golyshin, P. N. Obligate oil-degrading marine bacteria. *Curr. Opin. Biotech.* **18,** 257-266 (2007).
34. Pepi, M. *et al.* Mercury-resistant bacterial strains *Pseudomonas* and *Psychrobacter* spp. isolated from sediments of Orbetello Lagoon (Italy) and their possible use in bioremediation processes. *Int. Biodet. Biodeg.* **65,** 85-91 (2011).
35. Michaud, L., Di Marco, G., Bruni, V. & Lo Giudice, A. Biodegradative potential and characterization of psychrotolerant polychlorinated biphenyl-degrading marine bacteria isolated from a coastal station in the Terra Nova Bay (Ross Sea, Antarctica). *Mar. Pollut. Bull.* **54,** 1754-1761 (2007).
36. Jin, H. M., Kim, J. M., Lee, H. J., Madsen, E. L. & Jeon, C. O. *Alteromonas* as a key agent of polycyclic aromatic hydrocarbon biodegradation in crude oil-contaminated coastal sediment. *Environ. Sci. Technol.* **46,** 7731-7740 (2012).
37. Abed, R. M. M., Musat, N., Musat, F. & Mußmann. M. Structure of microbial communities and hydrocarbon-dependent sulfate reduction in the anoxic layer of a polluted microbial mat. *Mar. Pollut. Bull.* **62,** 539-546 (2011).
38. Jaekel, U., Musat, N., Adam, B., Kuypers, M., Grundmann, O. & Musat, F. Anaerobic degradation of propane and butane by sulfate-reducing bacteria enriched from marine hydrocarbon cold seeps. *ISME J.* **7,** 885-895 (2013).
39. Sitte, J. *et al.* Microbial links between sulfate reduction and metal retention in uranium- and heavy metal-contaminated soil. *Appl. Environ. Microbiol.* **76,** 3143-3152 (2010).

40. Isaac, P., Sánchez, L. A., Bourguignon, N., Cablar M. E. & Ferrero M. A. Indigenous PAH-degrading bacteria from oil-polluted sediments in Caleta Cordova, Partagonia Argentina. *Int. Biodet. Biodeg.* **82,** 207-214 (2013).

41. Gilbert, J. A. *et al.* Defining seasonal marine microbial community dynamics. *ISME J.* **6,** 298-308 (2012).

42. Zahed, M. A. *et al.* Kinetic modeling and half life study on bioremediation of crude oil dispersed by Corexit 9500. *J. Hazard Mater.* **185,** 1027-1031 (2011).

43. Bauer, M. *et al.* Whole genome analysis of the marine Bacteroidetes '*Gramella forsetii*' reveals adaptations to degradation of polymeric organic matter. *Environ. Microbiol.* **8,** 2201-2213 (2006).

44. Chen, Y. *et al. Salinimicrobium terrae* sp. nov., isolated from saline soil, and emended description of the genus *Salinimicrobium. Int. J. Syst. Evol. Microbiol.* **58,** 2501-2504 (2008).

45. Peura, S. *et al.* Distinct and diverse anaerobic bacterial communities in boreal lakes dominated by candidate division OD1. *ISME J.* **6,** 1640-1652 (2012).

46. Seifert, J. *et al.* Bioinformatic progress and applications in metaproteogenomics for bridging the gap between genomic sequences and metabolic functions in microbial communities. *Proteomics* **13,** 2786-2804 (2013).

47. Attias, L. *et al.* Crude oil spill in sea water: an assessment of the risk for bathers correlated to benzo(a)pyrene exposure. *Cent. Eur. J. Public. Health* **3,** 142-145 (1995).

48. Genovese, M. *et al.* Effective bioremediation strategy for rapid in situ cleanup of anoxic marine sediments in mesocosm oil spill simulation. *Front. Microbiol.* **5,** 162 (2014).

49. Denaro, R. *et al.* Assessing terminal restriction fragment length polymorphism suitability for the description of bacterial community structure and dynamics in hydrocarbon-polluted marine environments. *Environ. Microbiol.* **7,** 78-87 (2005).

50. Nikolopoulou, M., Eickenbusch, P., Pasadakis, N., Venieri, D. & Kalogerakis, N. Microcosm evaluation of autochthonous bioaugmentation to combat marine oil spills. *N. Biotechnol.* **30,** 734-742 (2013).

51. Cappello, S. *et al.* Characterisation of oil-degrading bacteria isolated from Bilge water. *Water Air. Soil Poll.* **223,** 3219-3226 (2012).

52. Ben Said, O., Goñi-Urriza, M., El Bour, M., Aissa, P. & Duran, R. Bacterial community structure of sediments of the bizerte lagoon (Tunisia), a southern Mediterranean coastal anthropized lagoon. *Microb Ecol.* **59,** 445-456 (2010).

53. Barhoumi, B. *et al.* Polycyclic aromatic hydrocarbons (PAHs) in surface sediments from the Bizerte Lagoon, Tunisia: levels, sources, and toxicological significance. *Environ. Monit. Assess.*

186, 2653-2669 (2014).

54. Ben Chekroun, K. *et al.* Role of macroalgae in biomonitoring of pollution in «Marchica», the Nador lagoon. *Phyton (B. Aires)* **82,** 31-34 (2013).
55. Ruiz, F., *et al.* (2006). The present environ-mental scenario of the Nador Lagoon (Morocco). *Environ. Res.* **102,** 215-229 (2006).
56. González, I., Águila. E. & Galán E. Partitioning, bioavailability and origin of heavy metals from the Nador Lagoon sedi-ments (Morocco) as a basis for their management. *Environ. Geol.* **52,** 1581-1593 (2007).
57. Bloundi, M. K., Faure, P. & Duplay, J.Organic contamination identification in sediments from a Mediterranean coastal ecosystem: The case of the Nador lagoon (Eastern Morocco). *C. R. Geoscience* **340,** 840-849 (2008).
58. Piazza, R. *et al.* Polychlorinated biphenyls in sediments of selected coastal en-vironments in northern Morocco. *Mar. Poll. Bull.* **58,** 431-438 (2009).
59. Ranya, A. *et al.* Hydrocarbonoclastic marine bacteria in Mediterranean Sea, El-Max, Egypt: isolation, identification and site characterization. *JÖKULL* **64,** 223-249 (2014).
60. Ibrahim, H. A., Farag, A. M., Beltagy, E. A. & El-Shenawy, M. A. Microbial pollution indicators along the Egyptian coastal waters of Suez and Aqaba Gulfs and Red Sea. *J. Egypt. Public Health Assoc.* **86,** 111-118 (2011).
61. Al-Rousan, S. A., Al-Shloul, R. N., Al-Horani, F. A. & Abu-Hilal, A. H. Heavy metal contents in growth bands of Porites corals: record of anthropogenic and human developments from the Jordanian Gulf of Aqaba. *Mar. Pollut. Bull.* **54,** 1912-1922 (2007).
62. Al-Taani, A. A. *et al.* Status of trace metals in surface seawater of the Gulf of Aqaba, Saudi Arabia. *Mar. Pollut. Bull.* **86,** 582-590 (2014).
63. Al-Najjar, T., Rasheed, M., Ababneh, Z., Ababneh, A. & Al-Omarey, H. Heavy metals pollution in sediment cores from the Gulf of Aqaba, Red Sea. *Natural Science* **3,** 775-782 (2011).
64. Grasshoff, K. *et al. Methods of Seawater Analysis,* 3rd edn. Weinheim: WILEY-VCH, ch. 10, p. 162, ch. 11, p. 230, ch. 12, pp. 263–271 (1999).
65. Menzel, D.W. & Vaccaro R.F. The measurement of dissolved organic and particulate carbon in seawater. *Limnol. Oceanog.* **9,** 138-142 (1964).
66. Kamaruzzaman, B.Y., Siti Waznah, A., Ong, M.C., Shahbudin, S. & Jalal, K.C.A. Variability of Organic Carbon Content in Bottom Sediment of Pahang River Estuary, Pahang, Malaysia. *J Appl. Sci.* **9,** 4253-4257 (2009).

67. Van de Peer, Y., Chapelle, S. & De Wachter, R. A. Quantitative map of nucleotide substitution rates in bacterial rRNA. *Nucleic Acids Res.* **24,** 3381-3391 (1996).

68. Chakravorty, S., Helb, D., Burday, M., Connell, N. & Alland, D. A detailed analysis of 16S ribosomal gene segments for the diagnosis of pathogenic bacteria. *J. Microbiol. Methods* **69,** 330–339 (2007).

69. Caporaso, J. G. *et al.* QIIME allows analysis of high–throughput community sequencing data. *Nat. Meth.* **7,** 335-336 (2010).

70. Edgar, R. C. Search and clustering orders of magnitude faster than BLAST. *Bioinformatics* **26,** 2460-2461 (2010).

71. Kunin, V., Engelbrektson, A., Ochman, H., Hugenholtz, P. Wrinkles in the rare biosphere: pyrosequencing errors can lead to artificial inflation of diversity estimates. *Environ. Microbiol.* **12,** 118-123 (2010).

72. Wang, Q., Garrity, G. M., Tiedje, J. M. & Cole, J. R. Naive Bayesian classifier for rapid assignment of rRNA sequences into the new bacterial taxonomy. *Appl. Environ. Microbiol.* **73,** 5261-5267 (2007).

73. Lozupone, C., Lladser, M. E., Knights, D., Stombaugh, J. & Knight, R. UniFrac: an effective distance metric for microbial community comparison. *ISME J.* **5,** 169-172 (2011).

74. Hammer, Ø., Harper, D. A. T. & Ryan, P. D. PAST: paleontological statistics software package for education and data analysis. *Palaeontol. Electronica* **4,** 1 (2001).

75. Méndez-García, C. *et al.* Microbial stratification in low pH oxic and suboxic macroscopic growths along an acid mine drainage. *ISME J.* **8,** 1259-1274 (2014).

76. Li, R. *et al.* De novo assembly of human genomes with massively parallel short read sequencing. *Genome Res.* **20,** 265-272 (2010).

77. Li, R., Li, Y., Kristiansen, K. & Wang, J. SOAP: short oligonucleotide alignment program. *Bioinformatics* 24, 713-714 (2008).

78. Csardi, G. & Nepusz T. The igraph software package for complex network research. InterJournal, Complex systems, 1695 (2006).

79. R Development Core Team. R: A language and environment for statistical computing. R Foundation for Statistical Computing, Vienna, Austria. ISBN 3-900051-07-0. 2011; http://www.R-project.org/.

80. Rasband, W.S. ImageJ, U. S. National Institutes of Health, Bethesda, Maryland, USA, http://imagej.nih.gov/ij/, 1997-2012.

81. Dyksterhouse, S.E., Gray, J.P., Herwig, R.P., Lara, J.C. & Staley, J.T. *Cycloclasticus pugetii* gen. nov., sp. nov., an aromatic hydrocarbon-degrading bacterium from marine sediments. *Int. J.*

Syst. Bacteriol. **45,** 116–123 (1995).

82. Pérez-Cobas, A. E. *et al.* Gut microbiota disturbance during antibiotic therapy: a multi-omic approach. *Gut* **62,** 1591-1601 (2013).
83. Dunn, W. B., *et al.* Procedures for large-scale metabolic profiling of serum and plasma using gas chromatography and liquid chromatography coupled to mass spectrometry. *Nat. Protoc.* **6,** 1060-1083 (2011)

Supplementary Table S1 Overall physico-chemical characteristics of the investigated sediment samples

Location	Mediterranean Sea							Red Sea
Sample code	ELF	HAV	PRI	BIZ	ELMAX	MCh	MES	AQ
Depth (m)	15.7	78.0	6.0	1.0	9.2	32.0	1.0	18.0
[Pet Hyd] (ppm)[1,2]	500	260,000	4,000	116	1,822	5,100	1,000	2,400
Temperature (°C)	13.0	15.0	19.0	19.3	20.0	21.3	23.0	26.5
Dissolved O_2 (mg/L)[3]	6.04	6.0-6.5	0[3]	3.0	18.0	22.0	1.0-2.2 [3]	20.0
pH	7.5	8.05	6.85	7.76	7.59	8.62	7.37	8.30
Conductivity (Ms/cm)	57.0	49.0	49.0	13.1	77.0	53.6	70.0	89.0
Ammonium (mkmol/L)	20.18	0.6-0.7	420	8.4	8.8	60.0	7.0	8.5
Calcium (mg/L)	50.94	420	408	35.80	71.3	87.37	430	125.78
Diss_org_carb (mg/L)	143.0	5.00	125.00	1.00	59.53	130.00	50.00	26.00
Part_org _carb (µM)	1.20	1.40	1.89	2.80	1.37	2.01	1.44	2.29
[Microelements] (nM)[4]	150.3	392.0	883.0	238.03	411.0	67.3	408.0	238.03
Cells per g of sediment[5]	~2.30e^{+08} (1.40e^{+08})	~1.90e^{+09} (1.15e^{+09})	~4.04e $^{+08}$ (3.67e $^{+08}$)	~2.63e^{+08} (1.44e^{+08})	~3.03e^{+08} (1.98e^{+08})	~2.63e^{+08} (2.29e^{+08})	~2.22e^{+08} (1.41e^{+08})	~3.43e^{+08} (1.93e^{+08})

[1]Total petroleum hydrocarbon concentration

[2]BIZ site is chronically polluted; however, total hydrocarbon concentration is low as compared to other highly polluted sites herein investigated. BIZ sediments were collected near an Oil Refinery Industry. The site is characterized by a marked pollution due to the release of the refinery wastewater containing residual hydrocarbons. Since this is the seashore/beach, the sea waves could have an influence on the turnover of the pollutants, that can be spread through the seawater more rapidly, compared to common chronically polluted sediment, thus explaining the low total hydrocarbon concentration.

[3]Oxygen concentration as measured in the seawater immediately above the sediment sample. PRI is an anoxic site; MES is a micro-anaerobic environment.

[4]Microelements include Sc, Cr, Mn, Fe, Ni, Co, As, Se, Mo, Ag, Sn, Sb, Ba, La, Ce, Sm, Eu, Tb, Hf, Au, Hg, as well as heavy metals such as Zn, Cd, Pb and Cu.

[5]Standard deviation of triplicates per each of the duplicates is shown in brackets. For all other parameters, triplicate measurements per each of the sediment site duplicates were performed, with standard deviations lower than 5%.

Supplementary Table S2 Library coverage estimation and sequence diversity of 16S rRNA

Sample	Nr. reads per sample	N. OTU_{97}	% Coverage*	Shannon index**
ELF	20454	7679	0.79	8.10
PRI	6303	3012	0.69	7.39
HAV	7365	3054	0.75	7.32
MES	8772	1858	0.88	5.73
BIZ	9972	3058	0.81	6.75
AQ	6816	406	0.97	3.18
ElMAX	8243	478	0.98	4.24
MCh	14535	1065	0.96	4.07

*Library coverage was calculated as C = 1-n/N, where n is the number of OTUs without a replicate, and N is the total number of sequences; **Shannon diversity index calculated using PAST

Supplementary Table S3 List of the taxonomic groups, identified based on the results of 16S rRNA pyrosequencing, composing the bacterial communities in the polluted sediments collected in the Mediterranean Sea and the Aqaba Gulf (Red Sea). Data indicate the relative percentage of sequences associated to each taxonomic group referred to the total number of sequences.

	Mediterranean Sea							Red Sea
**TAXONOMIC CLASSIFICATION**	**ELF**	**HAV**	**PRI**	**BIZ**	**ELMAX**	**MCh**	**MES**	**AQ**
Temperature (°C)	13.0	15.0	19.0	19.3	20.0	21.3	23.0	26.5
Uncl. Acidimicrobiales	0.326	0.186	0.377	2.698	1.638	0.000	0.000	0.000
Corynebacterium	0.000	0.000	0.000	0.000	0.646	0.000	0.000	0.000
Propionibacterium	0.000	0.000	0.000	0.000	4.267	0.000	0.000	0.000
Uncl. Bacteroidales	0.148	0.371	1.508	0.502	0.000	0.000	1.308	0.000
Uncl. *Flavobacteriaceae*	2.370	2.414	3.676	37.527	1.181	0.000	21.661	0.000
Arenibacter	0.000	0.000	0.094	0.000	0.000	0.000	1.629	0.000
Gramella	0.000	0.000	0.000	0.000	0.000	4.152	0.000	0.000
Salinimicrobium	0.000	0.000	0.000	0.000	0.441	73.791	0.000	0.000
Uncl. *Chitinophagaceae*	0.000	0.000	0.000	0.000	2.267	0.000	0.000	0.000
Uncl. Ignavibacteriales	0.000	0.000	0.000	2.165	0.803	0.000	0.115	0.000
Uncl. Cyanobacteria	0.237	0.000	0.000	1.224	1.102	0.000	0.000	0.000
Uncl. *Bacillaceae*	0.000	0.000	0.000	0.000	0.000	9.376	0.000	0.000
Bacillus	0.000	0.000	0.000	0.000	13.998	1.162	0.000	0.000
Planomicrobium	0.000	0.000	0.000	0.000	2.724	0.000	0.000	0.000
Staphylococcus	0.000	0.000	0.000	0.000	1.417	0.000	0.000	0.000
Uncl. *Clostridiaceae*	0.000	0.000	0.094	0.000	0.756	2.225	0.344	0.000
Anaerococcus	0.000	0.000	0.000	0.000	1.086	0.000	0.000	0.000
Uncl. *Peptostreptococcaceae*	0.000	0.093	0.000	0.000	0.724	0.000	0.000	0.000
Propionigenium	1.303	0.000	0.377	0.000	0.000	0.000	0.000	0.000
Psychrilyobacter	0.415	0.186	4.147	0.000	0.000	0.000	0.023	0.000
Uncl. *Thermodesulfovibrionaceae*	1.244	0.093	0.754	0.000	0.000	0.000	0.000	0.000
Uncl. *Phycisphaerae*	3.110	0.093	0.000	0.000	0.000	0.000	0.000	0.000
Uncl. Phycisphaerales	0.000	0.650	13.666	0.000	0.000	0.000	9.890	0.000
Uncl. *Pirellulaceae*	0.000	0.000	0.000	0.031	2.047	0.000	0.000	0.000
Planctomyces	0.000	0.093	0.000	0.000	0.000	0.000	15.856	0.000
Uncl. Alphaproteobacteria	0.000	3.993	0.000	0.000	0.000	0.000	0.000	0.000
Uncl. *Caulobacteraceae*	0.000	0.000	0.000	0.000	21.083	0.000	0.000	0.000
Uncl. *Bradyrhizobiaceae*	0.000	0.000	0.000	0.000	10.219	0.000	0.000	0.000
Bradyrhizobium	0.000	0.000	0.000	0.000	3.196	0.000	0.000	0.000

Uncl. *Methylobacteriaceae*	0.000	0.000	0.000	0.000	8.235	0.000	0.000	0.000
Methylobacterium	0.000	0.000	0.000	0.000	3.527	0.000	0.000	0.000
Mesorhizobium	0.000	0.000	0.000	0.000	0.000	0.000	0.918	0.000
Uncl. *Rhodobacteraceae*	1.037	1.114	0.471	6.997	0.047	0.000	0.780	2.111
Nautella	0.000	0.000	0.000	0.000	0.000	0.000	0.000	0.704
Paracoccus	0.000	0.000	0.189	0.000	0.000	0.018	0.000	6.668
Phaeobacter	0.000	0.093	0.094	0.345	0.000	0.000	0.023	1.689
Ruegeria	0.237	0.093	0.283	1.130	0.000	0.000	0.000	0.018
Thalassobius	0.000	4.457	0.000	0.000	0.000	0.000	3.006	1.161
Uncl. *Rhodospirillaceae*	0.977	0.464	0.189	0.000	0.000	0.000	0.000	0.000
Uncl. Betaproteobacteria	0.000	4.271	0.000	0.000	0.000	0.000	0.000	0.000
Uncl. *Desulfobulbaceae*	6.309	1.021	11.027	1.255	0.047	0.000	0.000	0.000
Uncl. *Desulfuromonadaceae*	0.415	2.693	0.189	2.071	0.000	0.000	0.000	0.000
Uncl. *Desulfobacteraceae*	2.281	0.279	0.000	0.000	0.000	0.000	0.000	0.000
Desulfococcus	11.463	1.764	1.885	0.063	0.000	0.000	0.000	0.000
Desulfosarcina	1.866	0.650	4.430	3.765	0.000	0.000	0.000	0.000
Uncl. *Syntrophobacteraceae*	24.704	0.186	0.566	0.000	0.000	0.000	0.000	0.000
Arcobacter	2.251	0.093	0.000	0.000	0.000	7.106	0.000	0.000
Uncl. *Helicobacteraceae*	2.607	14.578	18.096	3.044	0.031	0.000	0.000	0.000
Sulfuricurvum	0.000	0.464	10.933	0.220	0.000	0.000	1.056	0.000
Sulfurimonas	0.000	0.000	4.430	11.672	0.000	0.000	0.000	0.000
Uncl. Gammaproteobacteria	4.532	42.154	9.331	0.157	0.000	0.000	0.184	0.000
Alteromonas	0.000	0.000	0.000	0.000	0.000	0.036	0.000	20.549
Marinobacter	0.000	0.000	0.000	2.855	0.000	0.000	0.207	0.000
Pseudoalteromonas	0.000	1.671	0.000	0.000	0.000	0.000	0.000	1.970
Uncl. Chromatiales	8.916	0.557	4.713	13.367	0.047	0.000	0.115	0.000
Uncl. Methylococcales	2.873	0.093	1.225	1.067	0.000	0.000	1.446	0.000
Uncl. *Halomonadaceae*	0.000	0.000	0.000	0.000	0.000	0.703	0.000	0.035
Psychrobacter	0.000	0.000	0.000	0.000	0.000	0.000	0.000	63.688
Pseudomonas	0.000	0.000	0.000	0.000	0.031	1.045	0.069	1.390
Uncl. *Piscirickettsiaceae*	0.000	4.550	0.943	0.000	0.000	0.000	24.254	0.000
Cycloclasticus	0.000	2.971	0.189	0.471	0.000	0.000	0.413	0.000
Methylophaga	0.000	0.371	0.000	0.031	0.000	0.000	3.580	0.000
Thiomicrospira	0.000	0.000	0.000	0.000	0.661	0.000	0.000	0.000
Uncl. *Vibrionaceae*	2.518	0.000	0.000	0.000	0.000	0.000	0.046	0.000
Vibrio	0.030	0.000	0.000	0.031	0.000	0.387	0.000	0.018
Uncl. Xanthomonadales	17.832	3.528	6.126	5.460	0.031	0.000	1.056	0.000

Uncl. *Sinobacteraceae*	0.000	0.000	0.000	0.000	7.794	0.000	0.000	0.000
Uncl. Spirochaetes	0.000	0.000	0.000	1.851	0.000	0.000	0.000	0.000
Uncl. OD1	0.000	0.000	0.000	0.000	0.000	0.000	10.55	0.000
Uncl. TM7	0.000	0.000	0.000	0.000	4.282	0.000	0.000	0.000
Uncl. AB16	0.000	3.714	0.000	0.000	0.000	0.000	0.000	0.000
Uncl. Bacteria	0.000	0.000	0.000	0.000	5.668	0.000	1.469	0.000

Supplementary Table S4 Enrichment in KEGG functional classes by meaning of the percentage of genes belonging to KEGG categories based on PICRUSt's prediction. As shown, no significant differences, including in biodegradation pathways (see in grey and bold), were observed.

**KEGG PATHWAY**	**KEGG PATHWAY**	**METABOLISM**	**PERCENTAGE OF GENES**							
			**ELF**	**HAV**	**PRI**	**BIZ**	**ELMAX**	**MCh**	**MES**	**AQ**
Cellular Processes	Cell Communication	Adherens junction	0,0000	0,0000	0,0000	0,0000	0,0000	0,0000	0,0000	0,0000
Cellular Processes	Cell Communication	Focal adhesion	0,0000	0,0000	0,0000	0,0001	0,0000	0,0000	0,0000	0,0000
Cellular Processes	Cell Communication	Tight junction	0,0000	0,0000	0,0000	0,0000	0,0000	0,0000	0,0000	0,0000
Cellular Processes	Cell Growth and Death	Apoptosis	0,0042	0,0314	0,0080	0,0210	0,0336	0,0340	0,0230	0,0527
Cellular Processes	Cell Growth and Death	Cell cycle	0,0000	0,0000	0,0000	0,0000	0,0000	0,0000	0,0000	0,0000
Cellular Processes	Cell Growth and Death	Cell cycle - Caulobacter	0,4735	0,5461	0,4866	0,4529	0,3920	0,4294	0,4902	0,3911
Cellular Processes	Cell Growth and Death	Cell cycle - yeast	0,0000	0,0000	0,0000	0,0000	0,0000	0,0000	0,0000	0,0000
Cellular Processes	Cell Growth and Death	Meiosis - yeast	0,0452	0,0400	0,0360	0,0415	0,0575	0,0324	0,0308	0,0333
Cellular Processes	Cell Growth and Death	Oocyte meiosis	0,0000	0,0000	0,0000	0,0000	0,0000	0,0000	0,0000	0,0000
Cellular Processes	Cell Growth and Death	p53 signaling pathway	0,0044	0,0314	0,0092	0,0210	0,0336	0,0340	0,0243	0,0527
Cellular Processes	Cell Motility	Bacterial chemotaxis	0,8726	0,7980	0,8039	0,5265	0,5051	0,2429	0,8249	0,5801
Cellular Processes	Cell Motility	Bacterial motility proteins	2,5071	2,0881	2,1701	1,3529	1,1616	0,3788	2,0324	1,4942
Cellular Processes	Cell Motility	Cytoskeleton proteins	0,2376	0,2165	0,2271	0,1975	0,2005	0,2137	0,2305	0,2323
Cellular Processes	Cell Motility	Flagellar assembly	1,0149	0,9070	0,8821	0,5533	0,5864	0,2099	0,7631	0,7310
Cellular Processes	Cell Motility	Regulation of actin cytoskeleton	0,0000	0,0000	0,0000	0,0000	0,0000	0,0000	0,0000	0,0000
Cellular Processes	Transport and Catabolism	Endocytosis	0,0005	0,0026	0,0018	0,0019	0,0061	0,0005	0,0030	0,0243
Cellular Processes	Transport and Catabolism	Lysosome	0,0269	0,1008	0,0408	0,0837	0,0383	0,1254	0,0652	0,0119
Cellular Processes	Transport and Catabolism	Peroxisome	0,2392	0,2077	0,2437	0,2455	0,2598	0,2541	0,2173	0,2680
Cellular Processes	Transport and Catabolism	Phagosome	0,0000	0,0000	0,0000	0,0000	0,0000	0,0000	0,0000	0,0000
Environmental Information Processing	Membrane Transport	ABC transporters	2,5575	2,8415	2,7343	2,6935	4,2431	1,2844	2,5389	3,7578
Environmental Information Processing	Membrane Transport	Bacterial secretion system	0,8171	0,8650	0,7856	0,7404	0,7023	0,5415	0,9955	0,7248
Environmental Information Processing	Membrane Transport	Phosphotransferase system (PTS)	0,0989	0,1074	0,0967	0,0622	0,1308	0,0299	0,0853	0,0811
Environmental Information Processing	Membrane Transport	Secretion system	2,0251	1,9260	1,8174	1,4516	1,3307	0,7669	2,0641	1,6424
Environmental Information Processing	Membrane Transport	Transporters	3,8496	4,2962	4,1925	4,2195	6,5019	2,2727	3,9000	5,6296
Environmental Information Processing	Signal Transduction	Calcium signaling pathway	0,0001	0,0002	0,0000	0,0000	0,0000	0,0000	0,0003	0,0000

Environmental Information Processing	Signal Transduction	ErbB signaling pathway	0,0000	0,0000	0,0000	0,0000	0,0000	0,0000	0,0000	0,0000
Environmental Information Processing	Signal Transduction	Hedgehog signaling pathway	0,0000	0,0000	0,0000	0,0000	0,0000	0,0000	0,0000	0,0000
Environmental Information Processing	Signal Transduction	MAPK signaling pathway	0,0000	0,0000	0,0000	0,0000	0,0000	0,0000	0,0000	0,0000
Environmental Information Processing	Signal Transduction	MAPK signaling pathway - yeast	0,0294	0,0346	0,0272	0,0458	0,0523	0,0703	0,0335	0,0462
Environmental Information Processing	Signal Transduction	Notch signaling pathway	0,0000	0,0000	0,0000	0,0000	0,0000	0,0000	0,0000	0,0000
Environmental Information Processing	Signal Transduction	Phosphatidylinositol signaling system	0,1007	0,1094	0,1030	0,1040	0,1154	0,0767	0,1240	0,0928
Environmental Information Processing	Signal Transduction	TGF-beta signaling pathway	0,0000	0,0000	0,0000	0,0000	0,0000	0,0000	0,0000	0,0000
Environmental Information Processing	Signal Transduction	Two-component system	2,2834	2,1741	2,1898	1,7531	1,8288	1,1684	2,3171	2,0243
Environmental Information Processing	Signal Transduction	VEGF signaling pathway	0,0000	0,0000	0,0000	0,0002	0,0028	0,0000	0,0000	0,0000
Environmental Information Processing	Signal Transduction	Wnt signaling pathway	0,0000	0,0000	0,0000	0,0000	0,0000	0,0000	0,0000	0,0000
Environmental Information Processing	Signal Transduction	mTOR signaling pathway	0,0000	0,0000	0,0000	0,0000	0,0000	0,0000	0,0000	0,0000
Environmental Information Processing	Signaling Molecules and Interaction	Bacterial toxins	0,0329	0,0369	0,0397	0,0397	0,0472	0,0372	0,0278	0,0254
Environmental Information Processing	Signaling Molecules and Interaction	CAM ligands	0,0000	0,0000	0,0000	0,0001	0,0000	0,0000	0,0000	0,0000
Environmental Information Processing	Signaling Molecules and Interaction	Cell adhesion molecules (CAMs)	0,0000	0,0000	0,0000	0,0000	0,0000	0,0000	0,0000	0,0000
Environmental Information Processing	Signaling Molecules and Interaction	Cellular antigens	0,0602	0,0549	0,0555	0,0998	0,0869	0,1646	0,0856	0,0971
Environmental Information Processing	Signaling Molecules and Interaction	Cytokine receptors	0,0000	0,0000	0,0000	0,0000	0,0000	0,0000	0,0000	0,0000
Environmental Information Processing	Signaling Molecules and Interaction	Cytokine-cytokine receptor interaction	0,0000	0,0000	0,0000	0,0000	0,0000	0,0000	0,0000	0,0000
Environmental Information Processing	Signaling Molecules and Interaction	ECM-receptor interaction	0,0000	0,0000	0,0000	0,0001	0,0000	0,0000	0,0000	0,0000
Environmental Information Processing	Signaling Molecules and Interaction	G protein-coupled receptors	0,0000	0,0000	0,0000	0,0000	0,0000	0,0000	0,0000	0,0000
Environmental Information Processing	Signaling Molecules and Interaction	GTP-binding proteins	0,0000	0,0000	0,0000	0,0000	0,0000	0,0000	0,0000	0,0089
Environmental Information Processing	Signaling Molecules and Interaction	Glycan bindng proteins	0,0000	0,0000	0,0000	0,0000	0,0000	0,0000	0,0000	0,0000

Environmental Information Processing	Signaling Molecules and Interaction	Ion channels	0,0033	0,0053	0,0045	0,0243	0,0126	0,0340	0,0066	0,0103
Environmental Information Processing	Signaling Molecules and Interaction	Neuroactive ligand-receptor interaction	0,0000	0,0000	0,0000	0,0000	0,0000	0,0000	0,0000	0,0000
Genetic Information Processing	Folding, Sorting and Degradation	Chaperones and folding catalysts	1,0328	0,9356	0,9794	0,8937	0,7336	0,7630	1,0505	0,7924
Genetic Information Processing	Folding, Sorting and Degradation	Proteasome	0,0313	0,0246	0,0291	0,0270	0,0164	0,0340	0,0287	0,0108
Genetic Information Processing	Folding, Sorting and Degradation	Protein export	0,5455	0,5653	0,5511	0,5446	0,4635	0,5022	0,5518	0,4424
Genetic Information Processing	Folding, Sorting and Degradation	Protein processing in endoplasmic reticulum	0,0679	0,0474	0,0635	0,0510	0,0364	0,0348	0,0467	0,0105
Genetic Information Processing	Folding, Sorting and Degradation	RNA degradation	0,4672	0,4786	0,4553	0,4532	0,3836	0,5199	0,4869	0,3819
Genetic Information Processing	Folding, Sorting and Degradation	Sulfur relay system	0,3759	0,3454	0,3381	0,3177	0,2804	0,1686	0,4082	0,2902
Genetic Information Processing	Folding, Sorting and Degradation	Ubiquitin system	0,0029	0,0035	0,0035	0,0059	0,0051	0,0002	0,0123	0,0097
Genetic Information Processing	RNA family	Non-coding RNAs	0,0000	0,0000	0,0000	0,0000	0,0009	0,0000	0,0000	0,0000
Genetic Information Processing	Replication and Repair	Base excision repair	0,3198	0,3717	0,3435	0,3610	0,3771	0,3620	0,3783	0,3091
Genetic Information Processing	Replication and Repair	Chromosome	1,3403	1,2858	1,2872	1,1837	1,0976	1,2343	1,3636	1,1835
Genetic Information Processing	Replication and Repair	DNA repair and recombination proteins	2,2391	2,2973	2,2780	2,2546	1,9933	2,6049	2,3406	1,9101
Genetic Information Processing	Replication and Repair	DNA replication	0,5098	0,5168	0,5184	0,5602	0,4542	0,6911	0,5270	0,4176
Genetic Information Processing	Replication and Repair	DNA replication proteins	0,8641	0,9029	0,8716	0,8965	0,7336	1,0523	0,9260	0,7259
Genetic Information Processing	Replication and Repair	Homologous recombination	0,7254	0,7207	0,7273	0,7528	0,5929	0,8637	0,7291	0,5714
Genetic Information Processing	Replication and Repair	Mismatch repair	0,6012	0,6072	0,6193	0,6290	0,5261	0,7342	0,6561	0,4938
Genetic Information Processing	Replication and Repair	Non-homologous end-joining	0,0090	0,0244	0,0137	0,0187	0,0397	0,0353	0,0155	0,0289
Genetic Information Processing	Replication and Repair	Nucleotide excision repair	0,2965	0,3003	0,3011	0,2849	0,2570	0,3177	0,2694	0,2147
Genetic Information Processing	Transcription	Basal transcription factors	0,0004	0,0005	0,0006	0,0018	0,0009	0,0015	0,0005	0,0003
Genetic Information Processing	Transcription	RNA polymerase	0,1320	0,1410	0,1339	0,1288	0,1149	0,1098	0,1410	0,1039
Genetic Information Processing	Transcription	Spliceosome	0,0001	0,0002	0,0004	0,0009	0,0000	0,0000	0,0047	0,0000

Genetic Information Processing	Transcription	Transcription factors	1,0167	1,0460	1,0523	1,2022	1,4289	1,1971	1,2313	1,5142
Genetic Information Processing	Transcription	Transcription machinery	0,7184	0,7328	0,7445	0,8303	0,6733	1,0922	0,7833	0,5241
Genetic Information Processing	Translation	Aminoacyl-tRNA biosynthesis	1,0350	1,0745	1,0556	0,9693	0,8738	0,8387	1,0024	0,8062
Genetic Information Processing	Translation	RNA transport	0,1155	0,0862	0,1088	0,1145	0,0799	0,2013	0,1186	0,0608
Genetic Information Processing	Translation	Ribosome	1,8221	1,9109	1,8719	1,8059	1,5176	1,9261	1,8525	1,3974
Genetic Information Processing	Translation	Ribosome Biogenesis	1,1894	1,1516	1,1508	1,1324	0,9518	1,1769	1,3417	1,0904
Genetic Information Processing	Translation	Ribosome biogenesis in eukaryotes	0,0478	0,0399	0,0434	0,0439	0,0322	0,0369	0,0610	0,0362
Genetic Information Processing	Translation	Translation factors	0,4433	0,4502	0,4522	0,4143	0,3402	0,4166	0,4229	0,3267
Genetic Information Processing	Translation	mRNA surveillance pathway	0,0021	0,0019	0,0023	0,0000	0,0000	0,0000	0,0000	0,0000
Human Diseases	Cancers	Bladder cancer	0,0019	0,0051	0,0043	0,0077	0,0150	0,0024	0,0204	0,0146
Human Diseases	Cancers	Chronic myeloid leukemia	0,0000	0,0000	0,0000	0,0000	0,0000	0,0000	0,0000	0,0000
Human Diseases	Cancers	Colorectal cancer	0,0040	0,0310	0,0078	0,0109	0,0308	0,0028	0,0213	0,0438
Human Diseases	Cancers	Glioma	0,0000	0,0000	0,0000	0,0000	0,0000	0,0000	0,0000	0,0000
Human Diseases	Cancers	Pancreatic cancer	0,0000	0,0000	0,0000	0,0000	0,0000	0,0000	0,0000	0,0000
Human Diseases	Cancers	Pathways in cancer	0,0599	0,0806	0,0602	0,0652	0,0710	0,0720	0,0832	0,0795
Human Diseases	Cancers	Prostate cancer	0,0247	0,0187	0,0217	0,0236	0,0136	0,0340	0,0275	0,0097
Human Diseases	Cancers	Renal cell carcinoma	0,0312	0,0310	0,0307	0,0304	0,0238	0,0351	0,0344	0,0260
Human Diseases	Cancers	Small cell lung cancer	0,0040	0,0310	0,0078	0,0112	0,0336	0,0028	0,0213	0,0438
Human Diseases	Cardiovascular Diseases	Arrhythmogenic right ventricular cardiomyopathy (ARVC)	0,0000	0,0000	0,0000	0,0000	0,0000	0,0000	0,0000	0,0000
Human Diseases	Cardiovascular Diseases	Dilated cardiomyopathy (DCM)	0,0000	0,0000	0,0000	0,0000	0,0000	0,0000	0,0000	0,0000
Human Diseases	Cardiovascular Diseases	Hypertrophic cardiomyopathy (HCM)	0,0005	0,0011	0,0012	0,0005	0,0000	0,0000	0,0060	0,0089
Human Diseases	Cardiovascular Diseases	Viral myocarditis	0,0040	0,0310	0,0078	0,0109	0,0308	0,0028	0,0213	0,0438
Human Diseases	Immune System Diseases	Primary immunodeficiency	0,0216	0,0215	0,0330	0,0376	0,0392	0,0368	0,0395	0,0495
Human Diseases	Immune System Diseases	Rheumatoid arthritis	0,0000	0,0000	0,0000	0,0000	0,0000	0,0000	0,0000	0,0000
Human Diseases	Immune System Diseases	Systemic lupus erythematosus	0,0004	0,0006	0,0000	0,0005	0,0000	0,0000	0,0014	0,0000
Human Diseases	Infectious Diseases	African trypanosomiasis	0,0013	0,0123	0,0025	0,0115	0,0093	0,0334	0,0117	0,0222
Human Diseases	Infectious Diseases	Amoebiasis	0,0011	0,0016	0,0010	0,0058	0,0154	0,0013	0,0030	0,0133
Human Diseases	Infectious Diseases	Bacterial invasion of epithelial cells	0,0043	0,0016	0,0049	0,0018	0,0000	0,0000	0,0000	0,0000
Human Diseases	Infectious Diseases	Chagas disease (American trypanosomiasis)	0,0017	0,0132	0,0037	0,0120	0,0084	0,0333	0,0177	0,0216

Human Diseases	Infectious Diseases	Epithelial cell signaling in Helicobacter pylori infection	0,0483	0,0375	0,0428	0,0400	0,0355	0,0056	0,0372	0,0360
Human Diseases	Infectious Diseases	Hepatitis C	0,0000	0,0000	0,0000	0,0000	0,0000	0,0000	0,0000	0,0000
Human Diseases	Infectious Diseases	Influenza A	0,0043	0,0310	0,0098	0,0111	0,0318	0,0029	0,0216	0,0438
Human Diseases	Infectious Diseases	Leishmaniasis	0,0000	0,0000	0,0000	0,0002	0,0028	0,0000	0,0000	0,0089
Human Diseases	Infectious Diseases	Measles	0,0000	0,0000	0,0000	0,0000	0,0000	0,0000	0,0000	0,0000
Human Diseases	Infectious Diseases	Pathogenic Escherichia coli infection	0,0000	0,0000	0,0000	0,0000	0,0000	0,0000	0,0000	0,0000
Human Diseases	Infectious Diseases	Pertussis	0,0385	0,0343	0,0387	0,0459	0,0313	0,0680	0,0511	0,0311
Human Diseases	Infectious Diseases	Shigellosis	0,0000	0,0000	0,0000	0,0000	0,0000	0,0000	0,0000	0,0000
Human Diseases	Infectious Diseases	Staphylococcus aureus infection	0,0001	0,0005	0,0025	0,0001	0,0065	0,0023	0,0021	0,0005
Human Diseases	Infectious Diseases	Toxoplasmosis	0,0040	0,0310	0,0078	0,0109	0,0308	0,0028	0,0213	0,0438
Human Diseases	Infectious Diseases	Tuberculosis	0,1501	0,1715	0,1491	0,1228	0,1341	0,0797	0,1346	0,1460
Human Diseases	Infectious Diseases	Vibrio cholerae infection	0,0005	0,0011	0,0012	0,0006	0,0000	0,0000	0,0059	0,0008
Human Diseases	Infectious Diseases	Vibrio cholerae pathogenic cycle	0,1386	0,0937	0,1147	0,0865	0,0579	0,0489	0,1370	0,1163
Human Diseases	Metabolic Diseases	Type I diabetes mellitus	0,0492	0,0472	0,0543	0,0462	0,0425	0,0355	0,0404	0,0352
Human Diseases	Metabolic Diseases	Type II diabetes mellitus	0,0491	0,0399	0,0438	0,0506	0,0341	0,0370	0,0466	0,0249
Human Diseases	Neurodegenerative Diseases	Alzheimer's disease	0,1304	0,1631	0,1264	0,1478	0,1439	0,1165	0,1801	0,1915
Human Diseases	Neurodegenerative Diseases	Amyotrophic lateral sclerosis (ALS)	0,0115	0,0420	0,0178	0,0188	0,0570	0,0707	0,0419	0,0879
Human Diseases	Neurodegenerative Diseases	Huntington's disease	0,0966	0,1382	0,0985	0,0897	0,1149	0,0817	0,1596	0,1688
Human Diseases	Neurodegenerative Diseases	Parkinson's disease	0,0685	0,1035	0,0672	0,0592	0,0818	0,0118	0,1159	0,1268
Human Diseases	Neurodegenerative Diseases	Prion diseases	0,0005	0,0016	0,0018	0,0009	0,0042	0,0324	0,0041	0,0165
Metabolism	Amino Acid Metabolism	Alanine, aspartate and glutamate metabolism	0,9059	0,9043	0,8966	0,9095	0,8541	1,0603	0,9137	0,8714
Metabolism	Amino Acid Metabolism	Amino acid related enzymes	1,3214	1,3183	1,3273	1,2839	1,1406	1,3191	1,2873	1,1018
Metabolism	Amino Acid Metabolism	Arginine and proline metabolism	1,1807	1,2406	1,1803	1,3513	1,3952	1,3716	1,3701	1,5088
Metabolism	Amino Acid Metabolism	Cysteine and methionine metabolism	0,7235	0,7246	0,7230	0,7685	0,6668	0,9166	0,7833	0,6829
Metabolism	Amino Acid Metabolism	Glycine, serine and threonine metabolism	0,7753	0,8647	0,7787	0,9992	1,0214	1,1366	0,9236	1,1956
Metabolism	Amino Acid Metabolism	Histidine metabolism	0,5248	0,5579	0,5739	0,6484	0,6341	0,8930	0,5887	0,6469
Metabolism	Amino Acid Metabolism	Lysine biosynthesis	0,6140	0,6765	0,6347	0,6237	0,5892	0,6734	0,6446	0,5904
Metabolism	Amino Acid Metabolism	Lysine degradation	0,5590	0,5830	0,5954	0,6336	0,7079	0,8030	0,6019	0,7851
Metabolism	Amino Acid Metabolism	Phenylalanine metabolism	0,3067	0,3499	0,3213	0,3793	0,4472	0,3925	0,3544	0,4186

Metabolism	Amino Acid Metabolism	Phenylalanine, tyrosine and tryptophan biosynthesis	0,8067	0,7814	0,7942	0,7740	0,6186	0,7425	0,7482	0,6412
Metabolism	Amino Acid Metabolism	Tryptophan metabolism	0,5812	0,6265	0,6337	0,7052	0,8612	0,7203	0,6438	0,9287
Metabolism	Amino Acid Metabolism	Tyrosine metabolism	0,3807	0,4630	0,4330	0,4939	0,5406	0,8599	0,4657	0,5733
Metabolism	Amino Acid Metabolism	Valine, leucine and isoleucine biosynthesis	0,6730	0,7087	0,6978	0,7712	0,7364	0,6824	0,6576	0,6837
Metabolism	Amino Acid Metabolism	Valine, leucine and isoleucine degradation	0,8343	0,9388	0,9239	1,0488	1,2695	1,0021	0,8910	1,4050
Metabolism	Biosynthesis of Other Secondary Metabolites	Betalain biosynthesis	0,0046	0,0038	0,0059	0,0036	0,0112	0,0003	0,0036	0,0076
Metabolism	Biosynthesis of Other Secondary Metabolites	Butirosin and neomycin biosynthesis	0,0401	0,0386	0,0453	0,0388	0,0374	0,0039	0,0315	0,0233
Metabolism	Biosynthesis of Other Secondary Metabolites	Caffeine metabolism	0,0003	0,0003	0,0012	0,0045	0,0033	0,0002	0,0051	0,0008
Metabolism	Biosynthesis of Other Secondary Metabolites	Clavulanic acid biosynthesis	0,0000	0,0000	0,0000	0,0000	0,0000	0,0000	0,0000	0,0003
Metabolism	Biosynthesis of Other Secondary Metabolites	Flavone and flavonol biosynthesis	0,0002	0,0026	0,0004	0,0004	0,0028	0,0311	0,0003	0,0000
Metabolism	Biosynthesis of Other Secondary Metabolites	Flavonoid biosynthesis	0,0215	0,0096	0,0172	0,0085	0,0206	0,0634	0,0117	0,0024
Metabolism	Biosynthesis of Other Secondary Metabolites	Indole alkaloid biosynthesis	0,0046	0,0038	0,0059	0,0036	0,0112	0,0003	0,0036	0,0076
Metabolism	Biosynthesis of Other Secondary Metabolites	Isoflavonoid biosynthesis	0,0000	0,0000	0,0000	0,0000	0,0009	0,0001	0,0000	0,0000
Metabolism	Biosynthesis of Other Secondary Metabolites	Isoquinoline alkaloid biosynthesis	0,0604	0,0637	0,0643	0,0633	0,0687	0,0687	0,0637	0,0654
Metabolism	Biosynthesis of Other Secondary Metabolites	Novobiocin biosynthesis	0,1544	0,1428	0,1475	0,1487	0,1257	0,1390	0,1437	0,1477
Metabolism	Biosynthesis of Other Secondary Metabolites	Penicillin and cephalosporin biosynthesis	0,0244	0,0289	0,0324	0,0401	0,0392	0,0389	0,0324	0,0446
Metabolism	Biosynthesis of Other Secondary Metabolites	Phenylpropanoid biosynthesis	0,0686	0,0735	0,0793	0,0973	0,0860	0,1998	0,0797	0,0938
Metabolism	Biosynthesis of Other	Stilbenoid, diarylheptanoid and gingerol	0,0203	0,0266	0,0176	0,0391	0,0425	0,0978	0,0228	0,0433

	Secondary Metabolites	biosynthesis								
Metabolism	Biosynthesis of Other Secondary Metabolites	Streptomycin biosynthesis	0,3009	0,2891	0,3136	0,2877	0,2589	0,2445	0,2454	0,1972
Metabolism	Biosynthesis of Other Secondary Metabolites	Tropane, piperidine and pyridine alkaloid biosynthesis	0,1482	0,1385	0,1497	0,1669	0,1346	0,1439	0,1635	0,1588
Metabolism	Biosynthesis of Other Secondary Metabolites	beta-Lactam resistance	0,0078	0,0147	0,0115	0,0175	0,0215	0,0355	0,0212	0,0119
Metabolism	Carbohydrate Metabolism	Amino sugar and nucleotide sugar metabolism	0,9509	0,9354	0,9730	0,9435	0,8481	1,0119	0,7987	0,7348
Metabolism	Carbohydrate Metabolism	Ascorbate and aldarate metabolism	0,1082	0,1294	0,1227	0,1327	0,1846	0,1851	0,1239	0,1693
Metabolism	Carbohydrate Metabolism	Butanoate metabolism	1,1473	1,1235	1,1846	1,1952	1,3163	0,9799	0,9627	1,2495
Metabolism	Carbohydrate Metabolism	C5-Branched dibasic acid metabolism	0,3113	0,3267	0,3285	0,3360	0,3355	0,2878	0,2998	0,3005
Metabolism	Carbohydrate Metabolism	Citrate cycle (TCA cycle)	0,8769	0,8813	0,8895	0,9371	0,8612	0,8804	0,8786	0,7242
Metabolism	Carbohydrate Metabolism	Fructose and mannose metabolism	0,5845	0,6283	0,6046	0,6321	0,5710	1,0648	0,5279	0,5512
Metabolism	Carbohydrate Metabolism	Galactose metabolism	0,2485	0,2578	0,3017	0,3228	0,3000	0,2849	0,2078	0,2650
Metabolism	Carbohydrate Metabolism	Glycolysis / Gluconeogenesis	1,0031	1,0330	1,0242	1,1208	1,1032	1,1666	0,9711	0,9747
Metabolism	Carbohydrate Metabolism	Glyoxylate and dicarboxylate metabolism	0,8344	0,7708	0,7926	0,8650	0,8644	0,7233	0,6904	0,8892
Metabolism	Carbohydrate Metabolism	Inositol phosphate metabolism	0,1060	0,1343	0,1227	0,1338	0,2056	0,0605	0,1194	0,1836
Metabolism	Carbohydrate Metabolism	Pentose and glucuronate interconversions	0,2119	0,2926	0,2566	0,4091	0,4163	0,4391	0,2688	0,4368
Metabolism	Carbohydrate Metabolism	Pentose phosphate pathway	0,5899	0,5830	0,6263	0,6415	0,6229	0,7813	0,5147	0,5655
Metabolism	Carbohydrate Metabolism	Propanoate metabolism	1,0416	1,0634	1,0920	1,1243	1,2807	0,8715	1,0035	1,2938
Metabolism	Carbohydrate Metabolism	Pyruvate metabolism	1,2236	1,1888	1,1985	1,2954	1,2775	1,2602	1,1181	1,2311
Metabolism	Carbohydrate Metabolism	Starch and sucrose metabolism	0,4850	0,4217	0,5024	0,5109	0,4173	0,7800	0,3923	0,3959
Metabolism	Energy Metabolism	Carbon fixation in photosynthetic organisms	0,5715	0,5476	0,5585	0,5603	0,4953	0,5942	0,4852	0,4373
Metabolism	Energy Metabolism	Carbon fixation pathways in prokaryotes	1,3421	1,2524	1,3656	1,3221	1,1952	1,0826	1,1621	1,1583
Metabolism	Energy Metabolism	Methane metabolism	1,1283	1,0249	1,1217	1,0117	1,0513	0,6871	0,9296	1,0015
Metabolism	Energy Metabolism	Nitrogen metabolism	0,7525	0,7521	0,7375	0,8545	0,7714	0,8184	0,8607	0,7508
Metabolism	Energy Metabolism	Oxidative phosphorylation	1,6082	1,6521	1,5227	1,3937	1,3214	1,0390	1,5134	1,2335
Metabolism	Energy Metabolism	Photosynthesis	0,3883	0,3740	0,3465	0,3084	0,2504	0,2823	0,2922	0,2277
Metabolism	Energy Metabolism	Photosynthesis - antenna proteins	0,0134	0,0000	0,0000	0,0000	0,0000	0,0000	0,0000	0,0000

Metabolism	Energy Metabolism	Photosynthesis proteins	0,4328	0,4121	0,3727	0,3286	0,2696	0,2861	0,3229	0,2596
Metabolism	Energy Metabolism	Sulfur metabolism	0,3261	0,3218	0,3009	0,3284	0,2509	0,3539	0,3522	0,2761
Metabolism	Enzyme Families	Cytochrome P450	0,0001	0,0000	0,0004	0,0000	0,0000	0,0002	0,0000	0,0005
Metabolism	Enzyme Families	Peptidases	1,4152	1,3429	1,4101	1,4870	1,3097	1,6304	1,4585	1,2981
Metabolism	Enzyme Families	Protein kinases	0,3670	0,3132	0,3344	0,2783	0,2827	0,1153	0,4180	0,3235
Metabolism	Glycan Biosynthesis and Metabolism	Glycosaminoglycan biosynthesis - chondroitin sulfate	0,0000	0,0000	0,0000	0,0000	0,0000	0,0000	0,0000	0,0000
Metabolism	Glycan Biosynthesis and Metabolism	Glycosaminoglycan biosynthesis - heparan sulfate	0,0000	0,0000	0,0000	0,0000	0,0000	0,0000	0,0000	0,0000
Metabolism	Glycan Biosynthesis and Metabolism	Glycosaminoglycan degradation	0,0137	0,0475	0,0193	0,0420	0,0229	0,0628	0,0314	0,0108
Metabolism	Glycan Biosynthesis and Metabolism	Glycosphingolipid biosynthesis - ganglio series	0,0009	0,0034	0,0020	0,0245	0,0173	0,0316	0,0047	0,0019
Metabolism	Glycan Biosynthesis and Metabolism	Glycosphingolipid biosynthesis - globo series	0,0079	0,0140	0,0182	0,0459	0,0341	0,0325	0,0083	0,0081
Metabolism	Glycan Biosynthesis and Metabolism	Glycosphingolipid biosynthesis - lacto and neolacto series	0,0000	0,0000	0,0000	0,0000	0,0000	0,0000	0,0000	0,0000
Metabolism	Glycan Biosynthesis and Metabolism	Glycosylphosphatidylinositol(GPI)-anchor biosynthesis	0,0089	0,0019	0,0029	0,0008	0,0000	0,0000	0,0002	0,0000
Metabolism	Glycan Biosynthesis and Metabolism	Glycosyltransferases	0,4314	0,3733	0,4366	0,3594	0,3121	0,3741	0,3511	0,2934
Metabolism	Glycan Biosynthesis and Metabolism	Lipopolysaccharide biosynthesis	0,5610	0,5123	0,5335	0,4284	0,3121	0,4486	0,5187	0,2956
Metabolism	Glycan Biosynthesis and Metabolism	Lipopolysaccharide biosynthesis proteins	0,6768	0,5988	0,6427	0,5271	0,3920	0,5507	0,6082	0,3619
Metabolism	Glycan Biosynthesis and Metabolism	N-Glycan biosynthesis	0,0355	0,0408	0,0442	0,0357	0,0332	0,0323	0,0329	0,0114
Metabolism	Glycan Biosynthesis and Metabolism	Other glycan degradation	0,0120	0,0268	0,0242	0,0945	0,0635	0,1882	0,0207	0,0262
Metabolism	Glycan Biosynthesis and Metabolism	Other types of O-glycan biosynthesis	0,0057	0,0016	0,0049	0,0018	0,0000	0,0000	0,0000	0,0000

Metabolism	Glycan Biosynthesis and Metabolism	Peptidoglycan biosynthesis	0,6444	0,6138	0,6449	0,6663	0,5504	0,6390	0,6177	0,4995
Metabolism	Glycan Biosynthesis and Metabolism	Various types of N-glycan biosynthesis	0,0001	0,0005	0,0012	0,0038	0,0056	0,0002	0,0048	0,0000
Metabolism	Lipid Metabolism	Arachidonic acid metabolism	0,0572	0,0592	0,0483	0,0540	0,0720	0,0744	0,0698	0,0960
Metabolism	Lipid Metabolism	Biosynthesis of unsaturated fatty acids	0,2721	0,2650	0,2679	0,3121	0,2958	0,2930	0,3166	0,3345
Metabolism	Lipid Metabolism	Ether lipid metabolism	0,0084	0,0051	0,0109	0,0143	0,0182	0,0008	0,0048	0,0252
Metabolism	Lipid Metabolism	Fatty acid biosynthesis	0,5446	0,6111	0,5763	0,6144	0,5378	0,7288	0,5581	0,4673
Metabolism	Lipid Metabolism	Fatty acid elongation in mitochondria	0,0089	0,0211	0,0139	0,0158	0,0290	0,0333	0,0162	0,0311
Metabolism	Lipid Metabolism	Fatty acid metabolism	0,8439	0,8754	0,9024	0,9009	1,1074	0,6204	0,7577	1,1756
Metabolism	Lipid Metabolism	Glycerolipid metabolism	0,2919	0,2818	0,3047	0,3136	0,3233	0,2706	0,2879	0,3132
Metabolism	Lipid Metabolism	Glycerophospholipid metabolism	0,5293	0,5123	0,5141	0,4831	0,4616	0,3855	0,5512	0,4497
Metabolism	Lipid Metabolism	Linoleic acid metabolism	0,0578	0,0831	0,0731	0,0941	0,0827	0,1667	0,0951	0,0852
Metabolism	Lipid Metabolism	Lipid biosynthesis proteins	0,8850	0,8437	0,8927	0,8713	0,8448	0,7957	0,7805	0,7464
Metabolism	Lipid Metabolism	Primary bile acid biosynthesis	0,0116	0,0101	0,0127	0,0146	0,0168	0,0022	0,0081	0,0065
Metabolism	Lipid Metabolism	Secondary bile acid biosynthesis	0,0041	0,0053	0,0049	0,0053	0,0093	0,0015	0,0047	0,0003
Metabolism	Lipid Metabolism	Sphingolipid metabolism	0,0515	0,1149	0,0758	0,1075	0,0701	0,0960	0,0656	0,0468
Metabolism	Lipid Metabolism	Steroid biosynthesis	0,0289	0,0233	0,0242	0,0148	0,0079	0,0014	0,0122	0,0005
Metabolism	Lipid Metabolism	Steroid hormone biosynthesis	0,0247	0,0450	0,0330	0,0424	0,0360	0,0023	0,0285	0,0252
Metabolism	Lipid Metabolism	Synthesis and degradation of ketone bodies	0,1382	0,1457	0,1544	0,1800	0,1981	0,2156	0,1301	0,2226
Metabolism	Lipid Metabolism	alpha-Linolenic acid metabolism	0,0442	0,0384	0,0500	0,0527	0,0491	0,0997	0,0623	0,0654
Metabolism	Metabolism of Cofactors and Vitamins	Biotin metabolism	0,2030	0,1832	0,1913	0,1647	0,1168	0,4240	0,1967	0,0811
Metabolism	Metabolism of Cofactors and Vitamins	Folate biosynthesis	0,4821	0,4694	0,4649	0,4898	0,3939	0,5586	0,5091	0,3832
Metabolism	Metabolism of Cofactors and Vitamins	Lipoic acid metabolism	0,0651	0,0683	0,0651	0,0674	0,0631	0,0706	0,0631	0,0527
Metabolism	Metabolism of Cofactors and Vitamins	Nicotinate and nicotinamide metabolism	0,4258	0,4177	0,4043	0,3818	0,3925	0,4004	0,3992	0,4186
Metabolism	Metabolism of Cofactors and	One carbon pool by folate	0,5215	0,5412	0,5274	0,5491	0,4920	0,6014	0,5503	0,4790

	Vitamins									
Metabolism	Metabolism of Cofactors and Vitamins	Pantothenate and CoA biosynthesis	0,5639	0,5787	0,5775	0,5595	0,5486	0,5425	0,5078	0,5301
Metabolism	Metabolism of Cofactors and Vitamins	Porphyrin and chlorophyll metabolism	0,9564	0,9924	0,9231	0,8413	0,9135	0,6591	0,8898	0,9179
Metabolism	Metabolism of Cofactors and Vitamins	Retinol metabolism	0,0812	0,1091	0,0920	0,0905	0,1182	0,1131	0,0996	0,1466
Metabolism	Metabolism of Cofactors and Vitamins	Riboflavin metabolism	0,2931	0,2999	0,2910	0,2699	0,2575	0,2863	0,2977	0,2699
Metabolism	Metabolism of Cofactors and Vitamins	Thiamine metabolism	0,4002	0,4091	0,4065	0,3272	0,3892	0,3373	0,3726	0,3218
Metabolism	Metabolism of Cofactors and Vitamins	Ubiquinone and other terpenoid-quinone biosynthesis	0,3287	0,2952	0,3101	0,3032	0,2495	0,3527	0,3406	0,2837
Metabolism	Metabolism of Cofactors and Vitamins	Vitamin B6 metabolism	0,1450	0,1662	0,1516	0,1647	0,1500	0,2103	0,1700	0,1655
Metabolism	Metabolism of Other Amino Acids	Cyanoamino acid metabolism	0,1563	0,1528	0,1712	0,2114	0,2425	0,3092	0,1721	0,2096
Metabolism	Metabolism of Other Amino Acids	D-Alanine metabolism	0,0970	0,0866	0,0889	0,0860	0,0850	0,0721	0,0948	0,0849
Metabolism	Metabolism of Other Amino Acids	D-Arginine and D-ornithine metabolism	0,0167	0,0128	0,0123	0,0101	0,0140	0,0014	0,0231	0,0241
Metabolism	Metabolism of Other Amino Acids	D-Glutamine and D-glutamate metabolism	0,1079	0,1061	0,1139	0,1249	0,1089	0,2016	0,1159	0,0949
Metabolism	Metabolism of Other Amino Acids	Glutathione metabolism	0,3588	0,4293	0,3373	0,3773	0,4467	0,3705	0,4654	0,5433
Metabolism	Metabolism of Other Amino Acids	Phosphonate and phosphinate metabolism	0,0239	0,0566	0,0326	0,0610	0,0766	0,0376	0,0542	0,0641
Metabolism	Metabolism of Other Amino Acids	Selenocompound metabolism	0,3319	0,3427	0,3430	0,4298	0,3341	0,5193	0,3399	0,2723
Metabolism	Metabolism of Other Amino Acids	Taurine and hypotaurine metabolism	0,1312	0,1423	0,1196	0,1456	0,1729	0,1114	0,1337	0,2115

Metabolism	Metabolism of Other Amino Acids	beta-Alanine metabolism	0,4695	0,5056	0,5159	0,5405	0,7013	0,3332	0,4747	0,7316
Metabolism	Metabolism of Terpenoids and Polyketides	Biosynthesis of 12-, 14- and 16-membered macrolides	0,0043	0,0016	0,0049	0,0018	0,0000	0,0001	0,0000	0,0000
Metabolism	Metabolism of Terpenoids and Polyketides	Biosynthesis of ansamycins	0,0637	0,0704	0,0702	0,0521	0,0533	0,0721	0,0532	0,0498
Metabolism	Metabolism of Terpenoids and Polyketides	Biosynthesis of siderophore group nonribosomal peptides	0,0147	0,0126	0,0203	0,0324	0,0383	0,0421	0,0198	0,0103
Metabolism	Metabolism of Terpenoids and Polyketides	Biosynthesis of type II polyketide backbone	0,0003	0,0010	0,0004	0,0044	0,0079	0,0000	0,0036	0,0076
Metabolism	Metabolism of Terpenoids and Polyketides	Biosynthesis of type II polyketide products	0,0000	0,0002	0,0000	0,0000	0,0000	0,0000	0,0003	0,0003
Metabolism	Metabolism of Terpenoids and Polyketides	Biosynthesis of vancomycin group antibiotics	0,0680	0,0614	0,0688	0,0630	0,0449	0,0662	0,0466	0,0262
Metabolism	Metabolism of Terpenoids and Polyketides	Carotenoid biosynthesis	0,0372	0,0313	0,0336	0,0423	0,0294	0,0956	0,0242	0,0211
Metabolism	Metabolism of Terpenoids and Polyketides	Geraniol degradation	0,3940	0,4273	0,4485	0,4828	0,5789	0,4340	0,4184	0,6566
Metabolism	Metabolism of Terpenoids and Polyketides	Limonene and pinene degradation	0,3897	0,4462	0,4465	0,5061	0,5953	0,7269	0,4504	0,6772
Metabolism	Metabolism of Terpenoids and Polyketides	Polyketide sugar unit biosynthesis	0,1730	0,1709	0,1765	0,1741	0,1201	0,2301	0,1389	0,0803
Metabolism	Metabolism of Terpenoids and Polyketides	Prenyltransferases	0,3790	0,3409	0,3557	0,3157	0,2607	0,2572	0,3101	0,2293
Metabolism	Metabolism of Terpenoids and Polyketides	Sesquiterpenoid biosynthesis	0,0000	0,0000	0,0000	0,0000	0,0000	0,0000	0,0000	0,0000
Metabolism	Metabolism of Terpenoids and Polyketides	Terpenoid backbone biosynthesis	0,5890	0,5484	0,5941	0,5180	0,4995	0,3807	0,5001	0,4500
Metabolism	Metabolism of Terpenoids and Polyketides	Tetracycline biosynthesis	0,1569	0,1675	0,1587	0,1719	0,1425	0,2099	0,1723	0,1431
Metabolism	Metabolism of Terpenoids	Zeatin biosynthesis	0,0347	0,0377	0,0352	0,0340	0,0280	0,0350	0,0347	0,0260

	and Polyketides									
Metabolism	Nucleotide Metabolism	Purine metabolism	1,9181	1,9038	1,9065	2,0164	1,8597	2,0126	2,0078	1,8669
Metabolism	Nucleotide Metabolism	Pyrimidine metabolism	1,3089	1,3635	1,3447	1,4889	1,2976	1,7997	1,4006	1,1775
**Metabolism**	**Xenobiotics Biodegradation and Metabolism**	**1,1,1-Trichloro-2,2-bis(4-chlorophenyl)ethane (DDT) degradation**	**0,0006**	**0,0014**	**0,0029**	**0,0222**	**0,0131**	**0,0625**	**0,0074**	**0,0160**
**Metabolism**	**Xenobiotics Biodegradation and Metabolism**	**Aminobenzoate degradation**	**0,3909**	**0,4118**	**0,4305**	**0,5046**	**0,6014**	**0,5728**	**0,3830**	**0,5990**
**Metabolism**	**Xenobiotics Biodegradation and Metabolism**	**Atrazine degradation**	**0,0601**	**0,0346**	**0,0453**	**0,0722**	**0,0892**	**0,1059**	**0,0455**	**0,0993**
**Metabolism**	**Xenobiotics Biodegradation and Metabolism**	**Benzoate degradation**	**0,4877**	**0,5147**	**0,5595**	**0,6407**	**0,7621**	**0,7149**	**0,4340**	**0,7234**
**Metabolism**	**Xenobiotics Biodegradation and Metabolism**	**Bisphenol degradation**	**0,1235**	**0,1597**	**0,1444**	**0,2305**	**0,1850**	**0,3645**	**0,1916**	**0,2126**
**Metabolism**	**Xenobiotics Biodegradation and Metabolism**	**Caprolactam degradation**	**0,2786**	**0,3001**	**0,3136**	**0,3020**	**0,3934**	**0,1647**	**0,2703**	**0,4419**
**Metabolism**	**Xenobiotics Biodegradation and Metabolism**	**Chloroalkane and chloroalkene degradation**	**0,2576**	**0,3647**	**0,2923**	**0,3120**	**0,3747**	**0,4265**	**0,3436**	**0,4384**
**Metabolism**	**Xenobiotics Biodegradation and Metabolism**	**Chlorocyclohexane and chlorobenzene degradation**	**0,0600**	**0,0796**	**0,0682**	**0,1120**	**0,1420**	**0,1049**	**0,0752**	**0,1431**
**Metabolism**	**Xenobiotics Biodegradation and Metabolism**	**Dioxin degradation**	**0,0209**	**0,0332**	**0,0250**	**0,0418**	**0,0678**	**0,0053**	**0,0230**	**0,0306**
**Metabolism**	**Xenobiotics**	**Drug metabolism - cytochrome P450**	**0,1318**	**0,2159**	**0,1423**	**0,1516**	**0,2514**	**0,1237**	**0,2128**	**0,3594**

	Biodegradation and Metabolism									
Metabolism	Xenobiotics Biodegradation and Metabolism	Drug metabolism - other enzymes	0,1597	0,1658	0,1747	0,2352	0,2397	0,3471	0,1976	0,2653
Metabolism	Xenobiotics Biodegradation and Metabolism	Ethylbenzene degradation	0,0885	0,0991	0,1079	0,1364	0,1280	0,3303	0,1015	0,1241
Metabolism	Xenobiotics Biodegradation and Metabolism	Fluorobenzoate degradation	0,0418	0,0429	0,0436	0,0547	0,0720	0,0390	0,0389	0,0617
Metabolism	Xenobiotics Biodegradation and Metabolism	Metabolism of xenobiotics by cytochrome P450	0,1253	0,2090	0,1346	0,1457	0,2364	0,1223	0,2100	0,3294
Metabolism	Xenobiotics Biodegradation and Metabolism	Naphthalene degradation	0,2306	0,3074	0,2708	0,3420	0,3944	0,6149	0,2962	0,4089
Metabolism	Xenobiotics Biodegradation and Metabolism	Nitrotoluene degradation	0,1843	0,1474	0,1796	0,1271	0,1420	0,0714	0,0861	0,1417
Metabolism	Xenobiotics Biodegradation and Metabolism	Polycyclic aromatic hydrocarbon degradation	0,1005	0,1466	0,1368	0,1749	0,1911	0,3374	0,1427	0,2199
Metabolism	Xenobiotics Biodegradation and Metabolism	Styrene degradation	0,0669	0,0670	0,0676	0,1032	0,1336	0,1107	0,0686	0,1166
Metabolism	Xenobiotics Biodegradation and Metabolism	Toluene degradation	0,1895	0,1817	0,1931	0,2219	0,2159	0,1484	0,2006	0,1993
Metabolism	Xenobiotics Biodegradation and	Xylene degradation	0,0166	0,0233	0,0184	0,0461	0,0458	0,0031	0,0185	0,0208

	Metabolism									
Organismal Systems	Circulatory System	Cardiac muscle contraction	0,0536	0,0645	0,0469	0,0473	0,0509	0,0088	0,0936	0,0773
Organismal Systems	Circulatory System	Vascular smooth muscle contraction	0,0000	0,0000	0,0000	0,0000	0,0000	0,0000	0,0000	0,0000
Organismal Systems	Development	Axon guidance	0,0000	0,0000	0,0000	0,0000	0,0000	0,0000	0,0000	0,0000
Organismal Systems	Digestive System	Bile secretion	0,0016	0,0040	0,0010	0,0051	0,0093	0,0001	0,0030	0,0097
Organismal Systems	Digestive System	Carbohydrate digestion and absorption	0,0084	0,0072	0,0072	0,0125	0,0075	0,0327	0,0033	0,0014
Organismal Systems	Digestive System	Fat digestion and absorption	0,0000	0,0000	0,0000	0,0000	0,0000	0,0000	0,0000	0,0000
Organismal Systems	Digestive System	Gastric acid secretion	0,0006	0,0019	0,0000	0,0002	0,0000	0,0000	0,0000	0,0000
Organismal Systems	Digestive System	Mineral absorption	0,0049	0,0048	0,0043	0,0022	0,0103	0,0015	0,0032	0,0162
Organismal Systems	Digestive System	Pancreatic secretion	0,0006	0,0019	0,0000	0,0002	0,0000	0,0000	0,0000	0,0000
Organismal Systems	Digestive System	Protein digestion and absorption	0,0022	0,0045	0,0043	0,0213	0,0028	0,0316	0,0125	0,0095
Organismal Systems	Digestive System	Salivary secretion	0,0006	0,0019	0,0000	0,0002	0,0000	0,0000	0,0000	0,0000
Organismal Systems	Endocrine System	Adipocytokine signaling pathway	0,1250	0,0892	0,1255	0,0996	0,1028	0,0406	0,0605	0,0673
Organismal Systems	Endocrine System	GnRH signaling pathway	0,0005	0,0026	0,0018	0,0019	0,0061	0,0005	0,0030	0,0243
Organismal Systems	Endocrine System	Insulin signaling pathway	0,0814	0,0578	0,0766	0,0571	0,0430	0,0346	0,0734	0,0435
Organismal Systems	Endocrine System	Melanogenesis	0,0000	0,0000	0,0000	0,0000	0,0000	0,0000	0,0000	0,0000
Organismal Systems	Endocrine System	PPAR signaling pathway	0,2411	0,2131	0,2519	0,2377	0,2668	0,0875	0,1757	0,2283
Organismal Systems	Endocrine System	Progesterone-mediated oocyte maturation	0,0247	0,0187	0,0217	0,0236	0,0136	0,0340	0,0275	0,0097
Organismal Systems	Endocrine System	Renin-angiotensin system	0,0006	0,0013	0,0012	0,0005	0,0009	0,0000	0,0060	0,0089
Organismal Systems	Environmental Adaptation	Circadian rhythm - mammal	0,0000	0,0000	0,0000	0,0000	0,0000	0,0000	0,0000	0,0000
Organismal Systems	Environmental Adaptation	Circadian rhythm - plant	0,0028	0,0027	0,0051	0,0039	0,0107	0,0322	0,0039	0,0000
Organismal Systems	Environmental Adaptation	Plant-pathogen interaction	0,1555	0,1567	0,1497	0,1331	0,1191	0,0812	0,1457	0,1320
Organismal Systems	Excretory System	Aldosterone-regulated sodium reabsorption	0,0050	0,0035	0,0049	0,0019	0,0000	0,0000	0,0000	0,0000
Organismal Systems	Excretory System	Endocrine and other factor-regulated calcium reabsorption	0,0006	0,0019	0,0000	0,0002	0,0000	0,0000	0,0000	0,0000
Organismal Systems	Excretory System	Proximal tubule bicarbonate reclamation	0,0286	0,0308	0,0250	0,0279	0,0346	0,0661	0,0234	0,0419
Organismal Systems	Excretory System	Vasopressin-regulated water reabsorption	0,0023	0,0024	0,0008	0,0037	0,0033	0,0002	0,0008	0,0005
Organismal Systems	Immune System	Antigen processing and presentation	0,0247	0,0187	0,0217	0,0236	0,0136	0,0340	0,0275	0,0097
Organismal Systems	Immune System	Complement and coagulation cascades	0,0000	0,0000	0,0000	0,0000	0,0000	0,0000	0,0000	0,0000
Organismal Systems	Immune System	Cytosolic DNA-sensing pathway	0,0000	0,0000	0,0000	0,0000	0,0000	0,0000	0,0000	0,0000

Organismal Systems	Immune System	Fc epsilon RI signaling pathway	0,0000	0,0000	0,0000	0,0000	0,0000	0,0000	0,0000	0,0000
Organismal Systems	Immune System	Fc gamma R-mediated phagocytosis	0,0005	0,0026	0,0018	0,0019	0,0061	0,0005	0,0030	0,0243
Organismal Systems	Immune System	Hematopoietic cell lineage	0,0000	0,0000	0,0000	0,0000	0,0000	0,0000	0,0000	0,0000
Organismal Systems	Immune System	Leukocyte transendothelial migration	0,0000	0,0000	0,0000	0,0000	0,0000	0,0000	0,0000	0,0000
Organismal Systems	Immune System	NOD-like receptor signaling pathway	0,0247	0,0187	0,0217	0,0237	0,0136	0,0340	0,0275	0,0103
Organismal Systems	Immune System	RIG-I-like receptor signaling pathway	0,0001	0,0003	0,0008	0,0012	0,0065	0,0016	0,0006	0,0011
Organismal Systems	Nervous System	Cholinergic synapse	0,0000	0,0000	0,0000	0,0000	0,0000	0,0000	0,0000	0,0000
Organismal Systems	Nervous System	Glutamatergic synapse	0,0627	0,0806	0,0705	0,0813	0,1009	0,1066	0,0829	0,1390
Organismal Systems	Nervous System	Long-term depression	0,0000	0,0000	0,0000	0,0000	0,0000	0,0000	0,0000	0,0000
Organismal Systems	Nervous System	Long-term potentiation	0,0000	0,0000	0,0000	0,0000	0,0000	0,0000	0,0000	0,0000
Organismal Systems	Nervous System	Neurotrophin signaling pathway	0,0000	0,0000	0,0000	0,0000	0,0000	0,0000	0,0000	0,0000
Organismal Systems	Sensory System	Olfactory transduction	0,0000	0,0000	0,0000	0,0000	0,0000	0,0000	0,0000	0,0000
Organismal Systems	Sensory System	Phototransduction	0,0000	0,0000	0,0000	0,0000	0,0000	0,0000	0,0000	0,0000
Organismal Systems	Sensory System	Phototransduction - fly	0,0000	0,0000	0,0000	0,0000	0,0000	0,0000	0,0000	0,0000
Unclassified	Cellular Processes and Signaling	Cell division	0,0887	0,0932	0,0801	0,0704	0,0607	0,0687	0,0806	0,0773
Unclassified	Cellular Processes and Signaling	Cell motility and secretion	0,3765	0,3587	0,3488	0,2679	0,2224	0,2147	0,3322	0,2337
Unclassified	Cellular Processes and Signaling	Electron transfer carriers	0,0676	0,0533	0,0662	0,0488	0,0551	0,0054	0,0206	0,0557
Unclassified	Cellular Processes and Signaling	Germination	0,0001	0,0000	0,0014	0,0001	0,0280	0,0157	0,0002	0,0000
Unclassified	Cellular Processes and Signaling	Inorganic ion transport and metabolism	0,3648	0,4006	0,3557	0,3897	0,3551	0,3311	0,4283	0,4308
Unclassified	Cellular Processes and Signaling	Membrane and intracellular structural molecules	0,8182	0,7526	0,7125	0,6745	0,6014	0,6573	0,8590	0,7426
Unclassified	Cellular Processes and Signaling	Other ion-coupled transporters	0,9446	0,9089	0,9310	0,9712	0,9742	1,2714	1,0478	1,0372
Unclassified	Cellular Processes and Signaling	Other transporters	0,2483	0,2143	0,2370	0,2099	0,2126	0,2777	0,2558	0,2118
Unclassified	Cellular Processes and	Pores ion channels	0,5956	0,4858	0,5249	0,4764	0,3841	0,6508	0,6389	0,5141

	Signaling									
Unclassified	Cellular Processes and Signaling	Signal transduction mechanisms	0,5981	0,4059	0,5005	0,4231	0,2990	0,4638	0,4749	0,4584
Unclassified	Cellular Processes and Signaling	Sporulation	0,0481	0,0239	0,0563	0,0324	0,1486	0,0635	0,0221	0,0095
Unclassified	Genetic Information Processing	Protein folding and associated processing	1,0307	0,8607	0,9337	0,8861	0,8037	0,8883	0,8910	0,8835
Unclassified	Genetic Information Processing	Replication, recombination and repair proteins	0,8231	0,7481	0,7549	0,6443	0,7228	0,7312	0,6308	0,6664
Unclassified	Genetic Information Processing	Restriction enzyme	0,2195	0,1388	0,1866	0,1211	0,0453	0,3159	0,1464	0,0381
Unclassified	Genetic Information Processing	Transcription related proteins	0,0058	0,0073	0,0039	0,0036	0,0056	0,0018	0,0011	0,0108
Unclassified	Genetic Information Processing	Translation proteins	0,8131	0,7586	0,7889	0,7777	0,6415	0,8807	0,8305	0,6574
Unclassified	Metabolism	Amino acid metabolism	0,3112	0,2952	0,3078	0,2735	0,2682	0,3834	0,2733	0,2842
Unclassified	Metabolism	Biosynthesis and biodegradation of secondary metabolites	0,0445	0,0456	0,0506	0,0600	0,0673	0,0737	0,0758	0,0892
Unclassified	Metabolism	Carbohydrate metabolism	0,0913	0,0858	0,0838	0,0682	0,0785	0,1066	0,0638	0,1071
Unclassified	Metabolism	Energy metabolism	0,9523	0,8965	0,9269	0,9331	0,7836	0,8825	0,9613	0,9252
Unclassified	Metabolism	Glycan biosynthesis and metabolism	0,0926	0,0751	0,0846	0,0698	0,0617	0,0380	0,0833	0,0768
Unclassified	Metabolism	Lipid metabolism	0,0999	0,1024	0,1010	0,0843	0,0841	0,0780	0,0832	0,1117
Unclassified	Metabolism	Metabolism of cofactors and vitamins	0,2393	0,2085	0,2206	0,2432	0,1986	0,1893	0,2482	0,2426
Unclassified	Metabolism	Nucleotide metabolism	0,0291	0,0177	0,0283	0,0229	0,0075	0,0020	0,0215	0,0187
Unclassified	Metabolism	Others	0,9212	1,0029	1,0085	1,5291	1,2083	4,2271	1,1118	1,2105
Unclassified	Poorly Characterized	Function unknown	1,7350	1,4927	1,5989	1,5933	1,5485	1,6406	1,8226	1,8655
Unclassified	Poorly Characterized	General function prediction only	3,3816	3,1066	3,3610	3,5606	3,3212	3,6420	3,3555	3,0344
			100	100	100	100	100	100	100	100

Supplementary Table S5 Complete information for catabolic network reconstruction.

(A) In-house codes used for graphical visualization of degradation networks. As described in text, each query sequence from the metagenome that matches a given protein family of the AromaDeg[24] is associated to a key catabolic enzyme for an aromatic degradation reaction. Based on bibliographic records[24-27], the substrates and intermediates products can be linked to form a biodegradation network[25-27]. For network reconstruction, each query sequence encoding a catabolic enzyme (with an in-house given "ec code") was assigned to a degradation reaction involving a metabolic substrate and a product with appropriated in-house assigned codes ("sp" codes"). The codes were further used for the network reconstruction using appropriated scripts and commands described in the Supplementary Methods. Panel A also shows the relative abundance (rel. ab.) of genes encoding each of the catabolic enzymes (to avoid artifacts due to differences in sample size) as found in the DNA (used for creating Supplementary Fig. S2) or 16 rRNA (used for creating Supplementary Fig. S3) data sets. The total rel. ab. for the both previous data sets ($\Sigma_{DNA+16SrRNA}$) is also shown (used to create Fig. 3). The data sets (only for DNA sequences) regarding Deepwater OV11 and BM058 sites, are also shown. Note that in all cases, the absolute number of genes corresponding to each rel. ab. is also shown. Genes/enzymes name abbreviations, corresponding to those mentioned in the text, are shown in brackets in the column D "Enzyme".

(B) List of substrate pollutants or intermediates possibly degraded by each of the communities herein examined, as inferred from the rel. ab. level (and absolute number, also shown) of genes encoding enzymes involved in their biodegradation in DNA (see Supplementary Fig. S2), 16S rRNA (Supplementary Fig. S3) and $\Sigma_{DNA+16SrRNA}$ (Fig. 3) data sets. Presumptive pollutants degraded by communities at Deepwater OV11 and BM058 sites (based on DNA data sets) are also shown. The total number of chemicals being potentially degraded by each of the communities, as well as the total number of ORF examined in each dataset, is shown at the bottom in the three datasets. Those pollutants from which validation were conducted are shown in shadowed grey color (column A, Panel B). The confidence value, that gives an estimation of the possibility that a given chemical is degraded, is also given, based on the data statistics reported in Supplementary Fig. S4.

The Table has been provided as a separate Excel table due to the extensive size. For raw data, please contact authors directly.

Supplementary Table S6 Data statistics for samples obtained by Illumina or 454 sequencing from the bacterial communities in the polluted sediments collected in the Mediterranean Sea and the Aqaba Gulf (Red Sea). For comparison data regarding samples at the Deepwater Horizon oil spill (panel E).

Supplementary Table S6A Raw sequencing data

Sample name	Insert size (bp)	Sequence type (bp)	Raw reads (Mbp)	High quality reads (Mbp)	Clean data rate (%)
MGS-HAV	170	Index 91 PE	2,000	1,981	99.00
MGS-MES	170	Index 91 PE	2,000	1,982	99.00
MGS-MCh	170	Index 91 PE	2,000	1,982	99.00
MGS-PRI	170	Index 91 PE	2,000	1,981	99.00
MGS-BIZ	170	Index 91 PE	1,987	1,962	99.00
	350	Index 91 PE	1,266	1,100	86.88
MGS-ELMAX	170	Index 91 PE	1,584	1,420	89.64
	350	Index 91 PE	1,149	1,100	95.73
MGS-AQ	564	454 GS FLX Ti	0.207	0.0517	24.98
	350	Index 91 PE	1,163	1,100	94.60

Supplementary Table S6B Data statistics for the best assembly results

Sample name	Contig	Total bp	N50	N90	Max	Min	Map to own
MGS-HAV	4,412	5,426,170	1,611	574	66,436	500	3.29
MGS-MES	20,103	28,484,805	2,019	593	102,834	500	15.86
MGS-MCh	24,955	27,378,482	1,164	572	241,119	500	20.42
MGS-PRI	1,855	4,379,134	7,634	710	62,941	500	4.35
MGS-BIZ	16,792	16,779,454	1,034	558	50,777	500	6.76
	12,691	19,364,101	2,076	622	62,304	500	66.99
MGS-ELMAX	3,133	2,982,628	1,629	367	14,075	200	4.50
	12,814	20,253,283	2,117	667	73,362	500	77.39
MGS-AQ	11,041	15,528,589	1,888		22,380	500	63.53
	8,371	21,504,196	12,101	753	273,210	500	86.23

Supplementary Table S6C Number of Open Reading Frames (ORF) and total assembled sequence

Sample name	ORFs	Total length (bp)	Avg. length (bp)
MGS-HAV	8,388	4,695,330	560.00
MGS-MES	40,077	25,562,526	638.00
MGS-MCh	44,522	24,811,614	557.00
MGS-PRI	5,858	3,872,385	661.00
MGS-BIZ	27,708	15,323,475	553.00
	27,893	17,270,982	619.19
MGS-ELMAX	3,481	2,346,126	674.00
	28,698	17,782,842	619.65
MGS-AQ	24,958	15,528,589	435.00
	26,866	19,154,985	712.98

Supplementary Table S6D Number of Open Reading Frames (ORF) with assigned function

Sample name	ORFs	Assigned to COG	Assigned to KEGG	Nr of COG	Nr of KEGG
MGS-HAV	8,388	4,500	3,797	1,990	1,719
MGS-MES	40,077	23,458	19,654	3,167	3,049
MGS-MCh	44,522	25,079	20,397	2,935	2,999
MGS-PRI	5,858	2,602	1,893	1,308	914
MGS-BIZ (total)	55,601	34,439	28,456	2,954	2,925
MGS-ELMAX (total)	32,179	41,869	38,223	3,125	3,245
MGS-AQ (total)	51,824	29,871	24,434	2,948	3,307

Supplementary Table S6E Number of Open Reading Frames (ORF) for samples BM058 (Longitude: -88.4375; Latitude: 28.672222; JGI project ID 403207; taxon IDs 2088090017 and 2081372002) and OV011 (Longitude: -88.4375; Latitude: 28.672222; JGI project ID 403191; taxon ID 2081372001) obtained from the Joint Genome Institute webpage (https://img.jgi.doe.gov/).

Sample name	ORFs	Total length (bp)
BM058	83,920	50,620,616
OV011	54,273	23,846,686

Supplementary Table S7 Metabolomic target data sets displaying the relative degradation of initial substrate pollutants (A) and the relative presence of key chemical intermediates (B), in the investigated enrichments. In panel A, the remaining relative concentration of the initial pollutants (referred to as "P" in the table) used to set up enrichment cultures is shown; 100%, no degradation of initial substrate pollutant; 0%, total degradation (absence of pollutant). In Panel B, values represent the area of the peak area (abbreviated as PA in the table) of key degradation intermediates (referred to as "I" in the table) in arbitrary units (a.u.), calculated on the basis of appropriate standards. The values were calculated, in triplicates per each of the duplicates sediment samples, by comparing the presence and abundance level after 3-weeks of the microcosm experiment compared to the initial point and after considering the controls assays. Standard deviations (SD) are shown.

Panel A	PRI		HAV		MES		MCh		BIZ		ELMAX		AQ	
	Rel. ab. (%)	SD	Rel. ab. (%)	SD	Rel. ab. (%)	SD	Rel. ab. (%)	SD	Rel. ab. (%)	SD	Rel. ab. (%)	SD	Rel. ab. (%)	SD
Benzoic acid (P)	100,00	0,00	36,17	7,03	10,86	1,60	44,96	4,78	21,85	4,61	22,49	11,75	5,24	3,83
Chlorobenzoic acid (P)	100,00	0,00	100,00	0,00	47,24	3,79	100,00	0,00	50,30	2,41	38,12	6,59	7,30	2,19
Nitrobenzoic (P)	62,14	5,01	69,61	5,16	16,91	0,37	28,18	2,45	51,50	2,86	26,07	1,77	4,77	0,77
Hydroxybenzoic acid (P)	63,06	7,11	66,89	5,28	15,18	1,18	31,57	1,91	48,87	3,06	34,20	2,07	4,63	0,54
Phthalic acid (P)	59,75	8,41	51,49	6,01	52,94	4,22	43,93	4,03	61,72	5,50	16,01	0,90	12,65	0,77
Isophthalic acid (P)	57,81	7,82	50,85	5,63	49,55	0,75	36,09	5,65	56,78	3,83	23,82	1,65	8,60	0,64
Terephthalic acid (P)	14,34	1,90	55,87	21,96	11,80	0,20	100,00	0,00	25,08	1,78	24,23	1,58	17,32	1,56
Anthracene (P)	69,86	4,80	100,00	0,00	100,00	0,00	24,26	12,09	5,18	1,52	14,92	4,85	5,96	0,67
2,3-Dihydroxybiphenyl (P)	100,00	0,00	95,61	10,61	17,15	0,40	45,97	0,13	57,74	9,89	60,51	4,36	16,42	1,68
4-Hydroxyphenylpyruvic acid (P)	70,34	4,15	69,59	24,05	10,38	10,46	16,77	1,72	27,35	5,88	29,13	4,38	3,10	0,03
Phenoxybenzoic acid (P)	11,25	1,01	73,63	5,96	10,51	0,45	23,12	2,23	30,95	2,02	70,86	6,23	58,70	4,45
Carbazole (P)	100,00	0,00	100,00	0,00	100,00	0,00	100,00	0,00	96,31	12,12	100,00	4,19	56,25	0,27
Phenol (P)	42,72	26,76	100,00	0,00	12,54	2,06	19,17	3,70	9,11	0,33	15,32	19,53	5,34	8,46
Trihydroxytoluene (P)	11,80	5,09	1,62	0,94	1,44	0,57	4,22	1,43	2,94	0,67	2,37	2,53	1,04	1,63
Gallic acid (P)	13,54	3,57	3,13	1,30	3,25	0,72	4,01	0,74	3,46	0,14	2,61	0,88	4,45	9,59
Panel B	PRI		HAV		MES		MCh		BIZ		ELMAX		AQ	
	PA (a.u.)	SD	PA (a.u.)	SD	PA (a.u.)	SD	PA (a.u.)	SD	PA (a.u.)	SD	PA (a.u.)	SD	PA (a.u.)	SD
Catechol (I)	10,76	0,78	9,51	1,34	6,53	0,87	8,46	1,48	5,22	2,05	6,18	2,30	6,93	2,23
Chlorocatechol (I)	3,15	0,08	2,67	0,28	0,75	0,00	0,50	0,01	0,43	0,09	0,57	0,10	1,27	0,28
Salicylic acid (I)	0,05	0,04	10,54	1,56	9,57	0,27	6,53	1,05	6,12	1,73	7,53	1,19	11,12	1,80
Muconic acid (I)	0,42	0,02	0,34	0,07	0,67	0,03	0,20	0,04	0,33	0,01	0,20	0,01	0,10	0,14
Gentisic acid (I)	2,03	0,96	0,00	0,00	21,02	0,48	14,07	1,41	17,10	0,88	16,89	1,08	19,96	1,69
Protocatechuic acid (I)	1,13	0,64	1,00	0,05	9,89	0,04	5,98	0,50	2,45	0,19	9,44	0,84	7,05	0,76
Homogentisic acid (I)	2,15	0,29	1,89	0,04	8,11	0,12	7,45	0,50	9,16	0,48	7,27	0,65	12,69	1,43
Myristic acid (I)	9,11	1,41	3,65	4,10	15,28	0,26	8,70	1,19	12,07	1,37	11,36	0,84	15,65	1,45
Homoprotocatechuic acid (I)	1,69	0,23	1,50	0,07	1,01	0,03	0,92	0,08	1,13	0,10	0,87	0,15	1,53	0,18

Supplementary Table S8 Putative metabolites identified and quantified by metabolomic approaches by LC-MS (-) and LC-MS (+) in the sediment samples. For differential quantitative metabolomics, we compared the metabolomes (in triplicates) of sediment samples by evaluating the peak area from the chromatographic peaks. A list of masses identified by LC-MS using positive and negative polarities following alignment are presented for samples HAV, MES, PRI and AQ. Because the samples interact during the separation technique and MS, it is crucial to employ quality controls (QCs) during LC-MS to ensure analytical reproducibility. Indeed, QC samples are required throughout the analytical runs at periodic intervals of time to monitor variations in signal across time and at the beginning of the sequence to stabilize the system QC samples were prepared for LC-MS by pooling and mixing equal volumes of each sample[83]. After gently vortexing, the mix was also filtered and subsequently transferred to an analytical vial and analysed. In all cases, the technique (LC-MS positive (+) or negative (-) mode), mass (in ppm) and retention time (RT; as ppm@RT), and the abundance level per sample are shown. Panel abbreviations and content as follows: LC(-) total and LC(+) total, list of statistically different masses obtained after alignment in LC-MS using negative (-) and positive (+) polarities, respectively; LC(-) HAV, LC(-)MES, LC(-)PRI and LC(-)AQ, list of statistically different masses obtained after alignment identified in HAV, MES, PRI and AQ samples as obtained using LC-MS using negative polarity; LC(+) HAV, LC(+)MES, LC(+)PRI and LC(+)AQ, list of statistically different masses obtained after alignment identified in HAV, MES, PRI and AQ samples in LC-MS using positive polarity. The Table has been provided as a separate Excel table due to the extensive size. For raw data, please contact authors directly.

Supplementary Fig. S1 Relative abundances of the different bacterial taxonomic groups at the (a) phylum and (b) class levels identified in the polluted sediments collected in the Mediterranean Sea and the Aqaba Gulf (Red Sea), as determined by pyrosequencing targeting the 16S rRNA gene. Bacterial sequences were classified based on the RDP Classifier through Qiime 1.6 (http://www.qiime.org/).

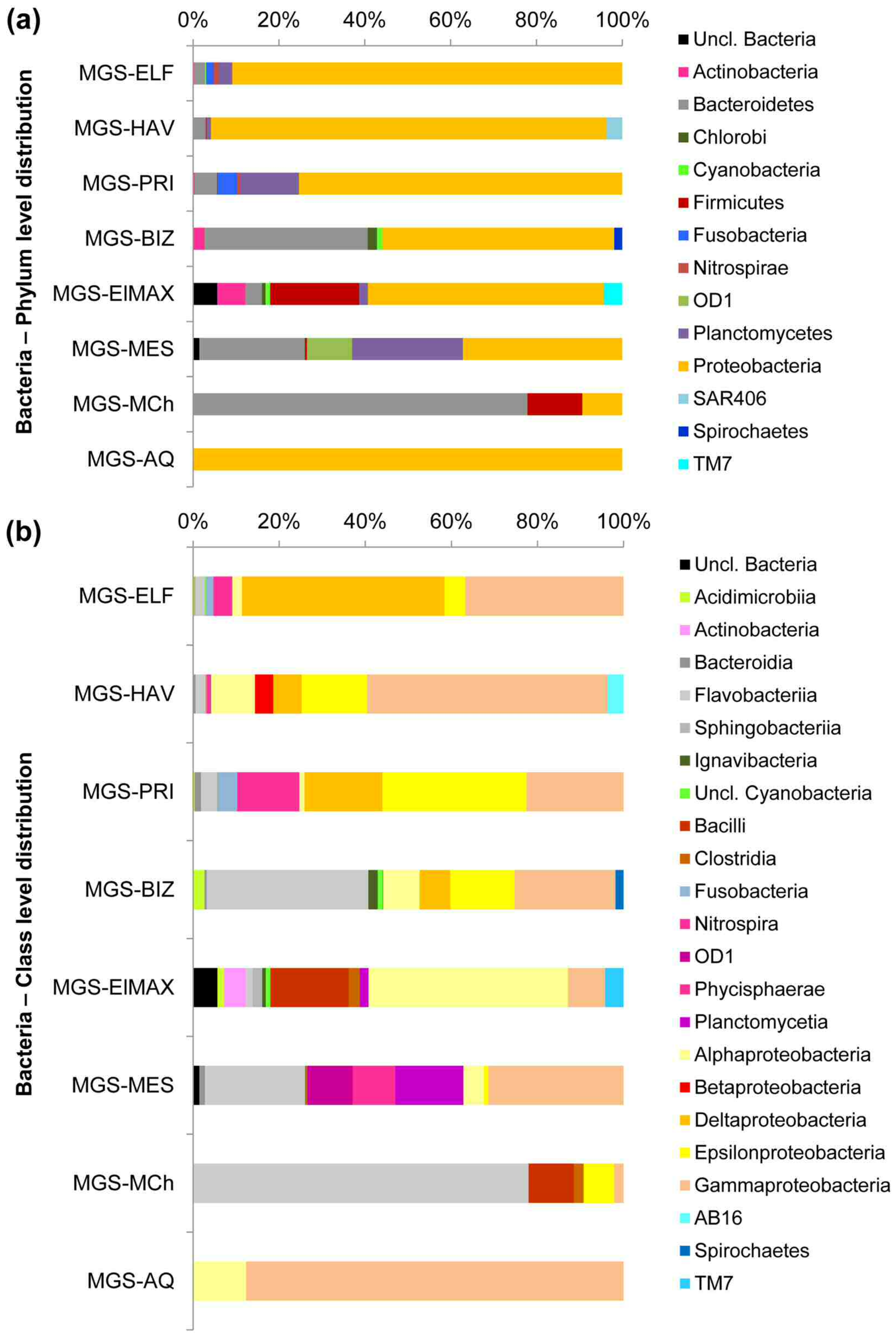

(a)
Bacteria – Phylum level distribution
0%
20%
40%
60%
80%
100%
MGS-ELF
MGS-HAV
MGS-PRI
MGS-BIZ
MGS-EIMAX
MGS-MES
MGS-MCh
MGS-AQ
Uncl. Bacteria
Actinobacteria
Bacteroidetes
Chlorobi
Cyanobacteria
Firmicutes
Fusobacteria
Nitrospirae
OD1
Planctomycetes
Proteobacteria
SAR406
Spirochaetes
TM7
(b)
Bacteria – Class level distribution
0%
20%
40%
60%
80%
100%
MGS-ELF
MGS-HAV
MGS-PRI
MGS-BIZ
MGS-EIMAX
MGS-MES
MGS-MCh
MGS-AQ
Uncl. Bacteria
Acidimicrobiia
Actinobacteria
Bacteroidia
Flavobacteriia
Sphingobacteriia
Ignavibacteria
Uncl. Cyanobacteria
Bacilli
Clostridia
Fusobacteria
Nitrospira
OD1
Phycisphaerae
Planctomycetia
Alphaproteobacteria
Betaproteobacteria
Deltaproteobacteria
Epsilonproteobacteria
Gammaproteobacteria
AB16
Spirochaetes
TM7

Supplementary Fig. S2 Potential key catabolic networks of alkanes and aromatics via di- and trihydroxylated intermediates in the investigated communities based on the metagenome sequences derived from sequenced DNA. The color code used for the respective pathways is shown. Codes for chemical species per pathway, as follows. *Napthalene biodegradation*: 3, 1,2-dihydroxynaphthalene; 14, 2-hydroxychromene-2-carboxylate. *Aniline biodegradation:* 32, 4-aminobenzene-1,2-diol. *Biphenyl biodegradation:* 51, biphenyl-2-3-diol; 39, benzoate. *Benzene biodegradation:* 48, cis-1,2-dihydrobenzene-1,2-diol. *Dibenzofuran/Dibenzo-p-dioxin biodegradation:* 5, 2,2',3-trihydroxybiphenyl; 101, 2-hydroxy-6-oxo-(2-hydroxyphenyl)-hexa-2,4-dienoic acid. *Chlorobenzoate biodegradation:* 84, 2-chlorocatechol; 107, chloromuconate. *Gallate biodegradation:* 76, (1E)-4-oxobut-1-ene-1,2,4-tricarboxylate. *Carbazole biodegradation:* 9, 2'-aminobiphenyl-2,3-diol; 13, 2-Hydroxy-6-oxo-6-(2'-aminophenyl)-hexa-2,4-dienoate (HOADA); 12, 2-hydroxypenta-2,4-dienoate; 37, anthranilate. *Cinnamate biodegradation:* 16, 2,3-dihydroxycinnamate. *Toluene and Xylene biodegradation:* 49, benzyl alcohol; 41, benzylsuccinate; 47, benzoyl-CoA; 22, toluene-cis-dihydrodiol; 31, 3-methylcatechol; 108, cis,cis-2-hydroxy-6-oxohepta-2,4-dienoate. *2-Aminophenol biodegradation:* 96, 2-aminomuconate 6-semialdehyde. *2,4-Dichlorophenoxyacetic acid biodegradation:* 18, 2,4-chlorophenol; 6, 2-chloromaleylacetic acid. *4-Hydroxypehnylpyruvate biodegradation:* 88, homogentisate; 106, maleylacetoacetate. *Alkane biodegradation:* 90, hydroxy alkane; 53, fatty acid. *2,4,5-Trichlorophenoxyacetic acid biodegradation:* 25, 2,4,5-trichlorophenol / 4-chlorocatechol / chlorohydroquinone. *4-Hydroxyphenylacetate biodegradation:* 89, homoprotocatechuate; 100, 2-hydroxy-5-carboxymethylmuconate semialdehyde. *Ibuprofen biodegradation:* 91, ibuprofen-CoA; 110, cis-1,2-diol-2-hydroibuprofen-CoA. *Quinoline biodegradation:* 15, 2-oxo-1,2-dihydroquinoline; 33, 8-hydroxy-2-oxo-1,2-dihydroquinoline. *p-Cumate biodegradation:* 75, 2,3-dihydroxy-p-cumate; 98, 2-hydroxy-3-carboxy-6-oxo-7-methylocta-2,4-dienoate. *Indole biodegradation:* 10, 2-formylaminobenzaldehyde. *Orcinol biodegradation:* 73, 2,3,5-trihydroxytoluene; 94, 2,4,6-trioxoheptanoate. *3-Methylgallate biodegradation:* 34, 4-carboxy-2-hydroxy-6-methoxy-6-oxohexa-2,4-dienoate; *2,4-Dinitrotoluene biodegradation:* 77, 2,4,5-trihydroxytoluene; 95, 2,4-dihydroxy-5-methyl-6-oxohexa-2,4-dienoate. *Hydroxyquinol biodegradation:* 111, 2-maleylacetate; 113, 3-oxoadipate. *Tetralin biodegradation:* 45, cis-1,2-Dihydroxy-1,2,5,6,7,8-hexahydronaphthalene. *PAH biodegradation:* 103, PAH dihydrodiol. *Abietane diterpenoid biodegradation*: 115, abietane diterpenoid intermediate (abietic acid/pallustric acid). *3-Nitrobenzoate biodegradation:* 21, p-hydroxyaminobenzoate. *2,4'-Dihydroxyacetophenone and p-Cresol biodegradation:* 28, 4-hydroxybenzoate. *Cholesterol/Sterol biodegradation:* 116, cholesterol/sterol intermediate. *Gentisate biodegradation*: 112, maleylpyruvate. *Phenanthrene biodegradation:* 17, 3,4-dihydroxyphenanthrene; 4, 1-hydroxy-2-naphthoate; 2, 2-carboxy-4-(2'-oxo-3,5-cyclohexadienyl)-buta-2,4-dienoic acid.

Protocatechuate biodegradation: 104, 3-carboxy-cis,cis-muconate; 99, 2-hydroxy-4-carboxymuconate-6-semialdehyde. *Catechol and 4-Methylcatechol biodegradation:* 124, cis,cis-2-hydroxy-6-oxohexa-2,4-dienoate; 109, cis,cis-muconic acid. The rel. ab. of each catabolic gene assigned to degradation reactions, as represented by the thickness of the lines in the figure, and the complete list of substrates possibly degraded by the communities are summarized in Supplementary Table S5.

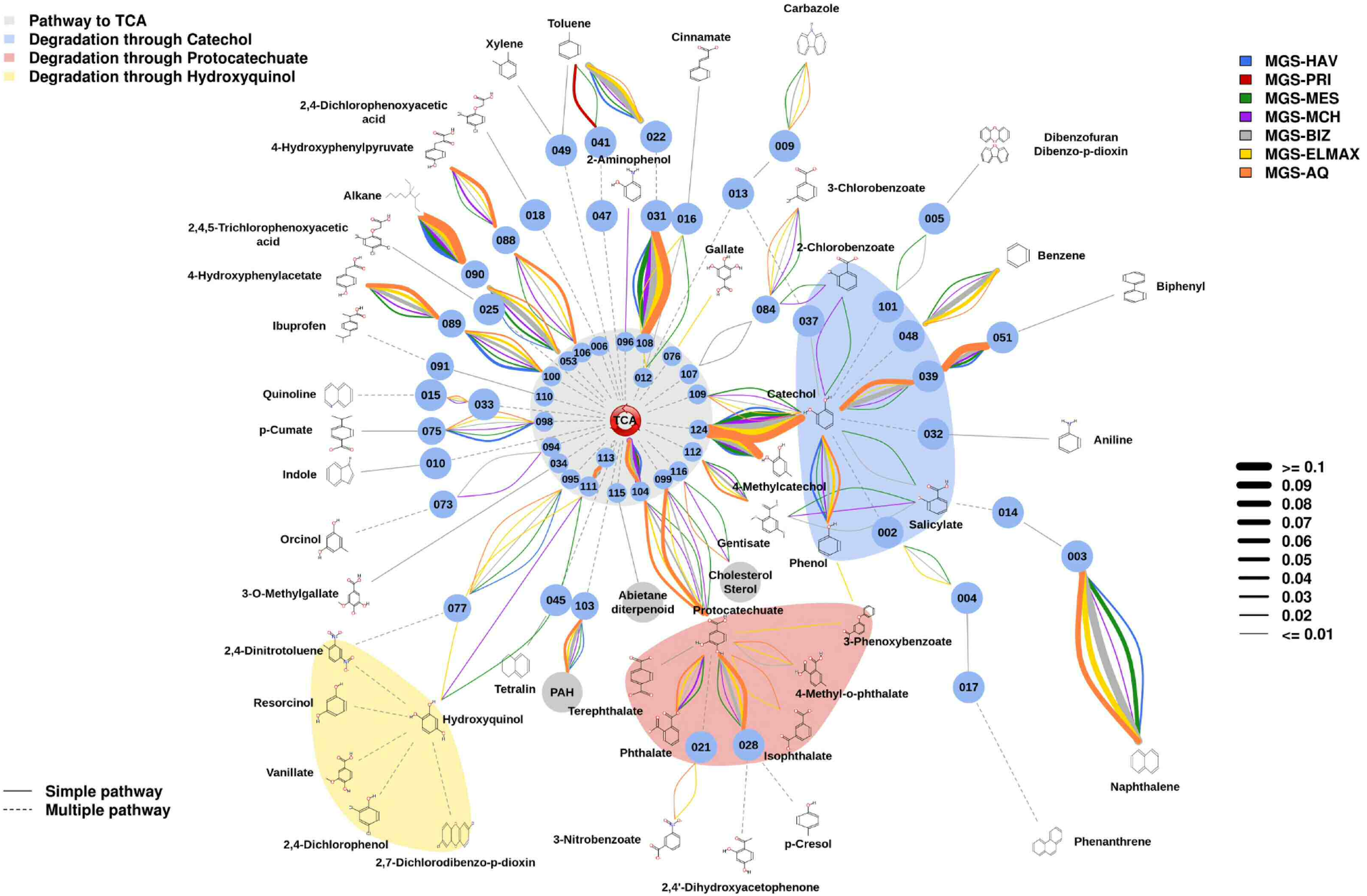

Pathway to TCA
Degradation through Catechol
Degradation through Protocatechuate
Degradation through Hydroxyquinol
MGS-HAV
MGS-PRI
MGS-MES
MGS-MCH
MGS-BIZ
MGS-ELMAX
MGS-AQ
>= 0.1
0.09
0.08
0.07
0.06
0.05
0.04
0.03
0.02
<= 0.01
Simple pathway
Multiple pathway
TCA
Toluene
Xylene
Cinnamate
Carbazole
2,4-Dichlorophenoxyacetic acid
4-Hydroxyphenylpyruvate
2-Aminophenol
3-Chlorobenzoate
Dibenzofuran
Dibenzo-p-dioxin
Alkane
2,4,5-Trichlorophenoxyacetic acid
Gallate
2-Chlorobenzoate
Benzene
4-Hydroxyphenylacetate
Biphenyl
Ibuprofen
Quinoline
Catechol
p-Cumate
Aniline
Indole
4-Methylcatechol
Orcinol
Gentisate
Salicylate
Phenol
Cholesterol
Sterol
3-O-Methylgallate
Abietane diterpenoid
Protocatechuate
3-Phenoxybenzoate
2,4-Dinitrotoluene
Tetralin
PAH
4-Methyl-o-phthalate
Resorcinol
Hydroxyquinol
Terephthalate
Phthalate
Isophthalate
Vanillate
Naphthalene
3-Nitrobenzoate
p-Cresol
Phenanthrene
2,4-Dichlorophenol
2,7-Dichlorodibenzo-p-dioxin
2,4'-Dihydroxyacetophenone
049
041
022
009
013
005
018
047
031
016
088
090
084
037
101
025
089
048
051
091
039
015
033
032
075
010
014
073
002
003
004
077
045
103
017
021
028
006
096
108
106
053
076
100
012
107
110
109
098
124
094
112
113
034
116
095
099
111
115
104

Supplementary Fig. S3 Potential key catabolic networks of alkanes and aromatics via di- and trihydroxylated intermediates in the investigated communities based on the 16S rRNA *in silico*-based metagenome. The color code used for the respective pathways is shown. The codes for the chemical species in each pathway are as described in Supplementary Fig. S2. The rel. ab. of each catabolic gene assigned to degradation reactions, as represented by the thickness of the lines in the figure, and the complete list of substrates possibly degraded by the communities are summarized in Supplementary Table S5. Note that the presumptive network includes an additional sample (ELF) for which DNA sequences could not be obtained.

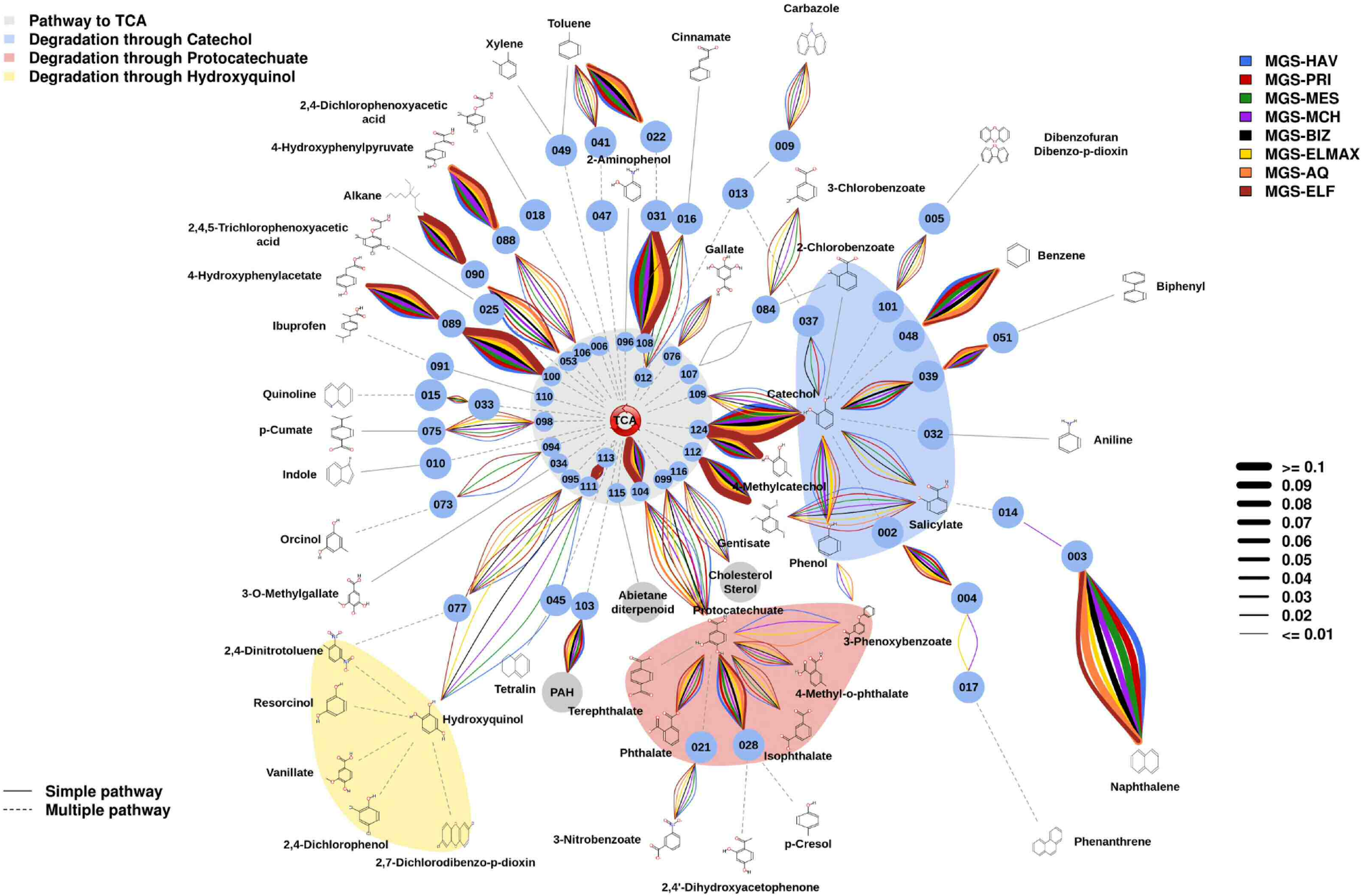

Pathway to TCA
Degradation through Catechol
Degradation through Protocatechuate
Degradation through Hydroxyquinol
MGS-HAV
MGS-PRI
MGS-MES
MGS-MCH
MGS-BIZ
MGS-ELMAX
MGS-AQ
MGS-ELF
>= 0.1
0.09
0.08
0.07
0.06
0.05
0.04
0.03
0.02
<= 0.01
Simple pathway
Multiple pathway
TCA
Toluene
Xylene
Cinnamate
Carbazole
2,4-Dichlorophenoxyacetic acid
4-Hydroxyphenylpyruvate
2-Aminophenol
3-Chlorobenzoate
Dibenzofuran
Dibenzo-p-dioxin
Alkane
2,4,5-Trichlorophenoxyacetic acid
Gallate
2-Chlorobenzoate
Benzene
Biphenyl
4-Hydroxyphenylacetate
Ibuprofen
Quinoline
p-Cumate
Indole
Catechol
Aniline
4-Methylcatechol
Salicylate
Orcinol
Gentisate
Phenol
Cholesterol
Sterol
3-O-Methylgallate
Abietane diterpenoid
Protocatechuate
3-Phenoxybenzoate
2,4-Dinitrotoluene
Tetralin
PAH
Terephthalate
4-Methyl-o-phthalate
Resorcinol
Hydroxyquinol
Phthalate
Isophthalate
Naphthalene
Vanillate
2,4-Dichlorophenol
2,7-Dichlorodibenzo-p-dioxin
3-Nitrobenzoate
p-Cresol
Phenanthrene
2,4'-Dihydroxyacetophenone
049
041
022
009
013
018
047
031
016
005
088
090
025
084
037
101
048
051
089
091
015
033
075
010
073
039
032
014
002
003
004
017
077
045
103
021
028
053
106
006
096
108
012
076
107
109
100
110
098
094
034
113
095
111
115
104
099
116
112
124

Supplementary Fig. S4 Summary statistics on the distribution of the confidence scores based on the number of genes associated to the degradation of a given chemical. Confidence was calculated on the basis of the minimum number of genes encoding catabolic enzymes involved in the degradation of a given chemical, and for which experimental metabolomics evidences were found (see Fig. 2). Based on the calculations, confidence intervals for each of the genes associated with pollutant degradation can be obtained and are given in Supplementary Table S5. As example, as shown in the figure, depending on the number of genes confidence can be calculated, and above a certain threshold a 100% confidence was achieved (the number above which pollutant degradation was confirmed in all cases). Statistics are given considering independent DNA, 16S rRNA and DNA + 16S rRNA datasets.

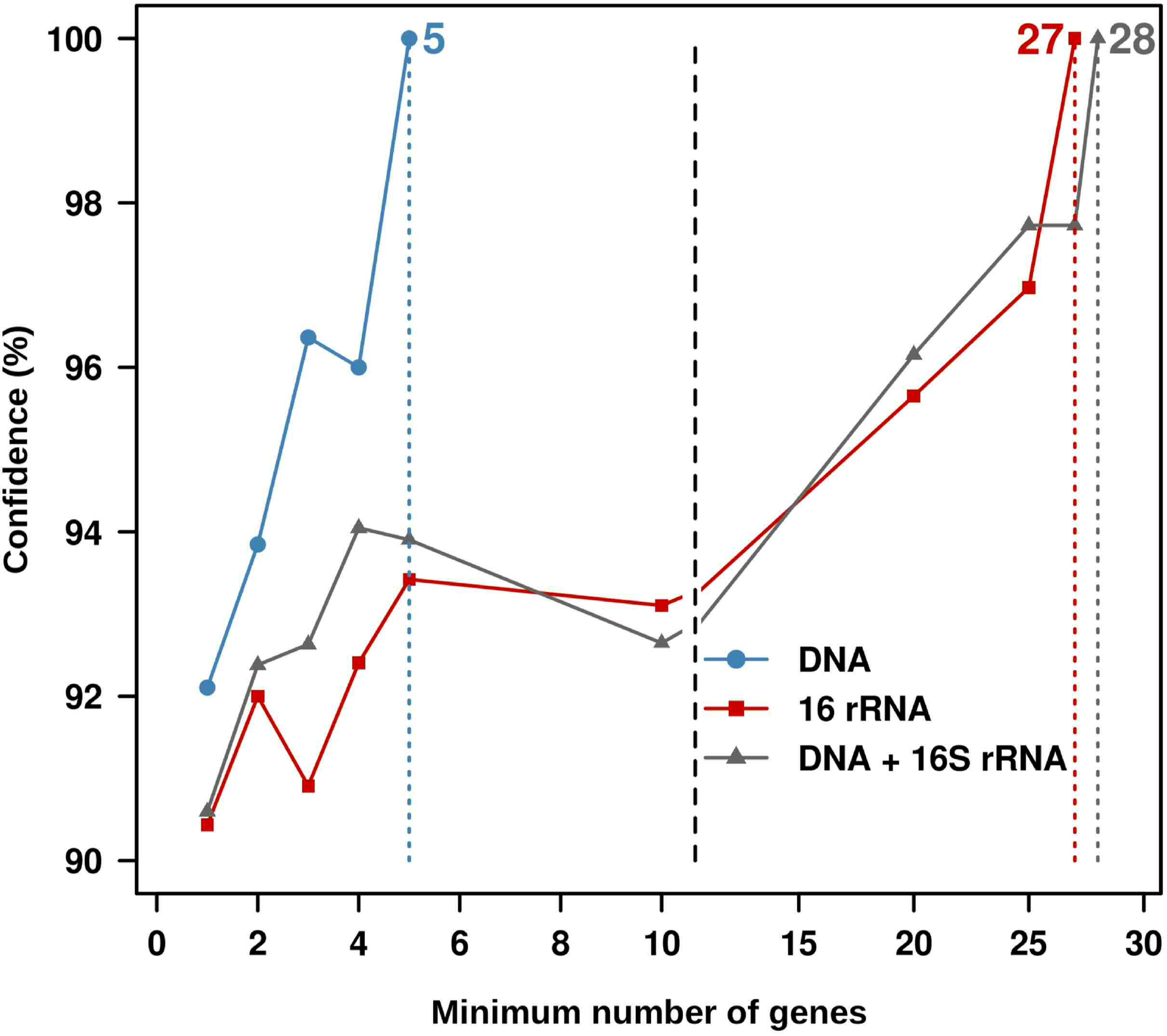
Confidence (%)
100
98
96
94
92
90
5
27
28
DNA
16 rRNA
DNA + 16S rRNA
0
2
4
6
8
10
15
20
25
30
Minimum number of genes

Supplementary Fig. S5 Temperature as environmental factor driving the size of biodegradation meta-webs at the eight studied sites. A significant positive correlation (r^2 ~ 0.8; $P = 3.78 \times 10^{-3}$; *t*-test) has been found between the relative percentage of genes encoding enzymes participating in biodegradation steps (DEGgp) based on the total number of genes (to avoid artifacts due to differences in sample size) identified as using 16S rRNA data sets (Panel A). When only gene percentages based on DNA datasets were considered (Panel B), the correlation was found at lower extend (r^2 ~ 0.42; $P = 4.194 \times 10^{-1}$; *t*-test); this is most likely due to the differences in sequence coverage. Both, the R-squared (r^2) and the *P*-value (*t*-test) for the regression are shown for data in Panels A and B.

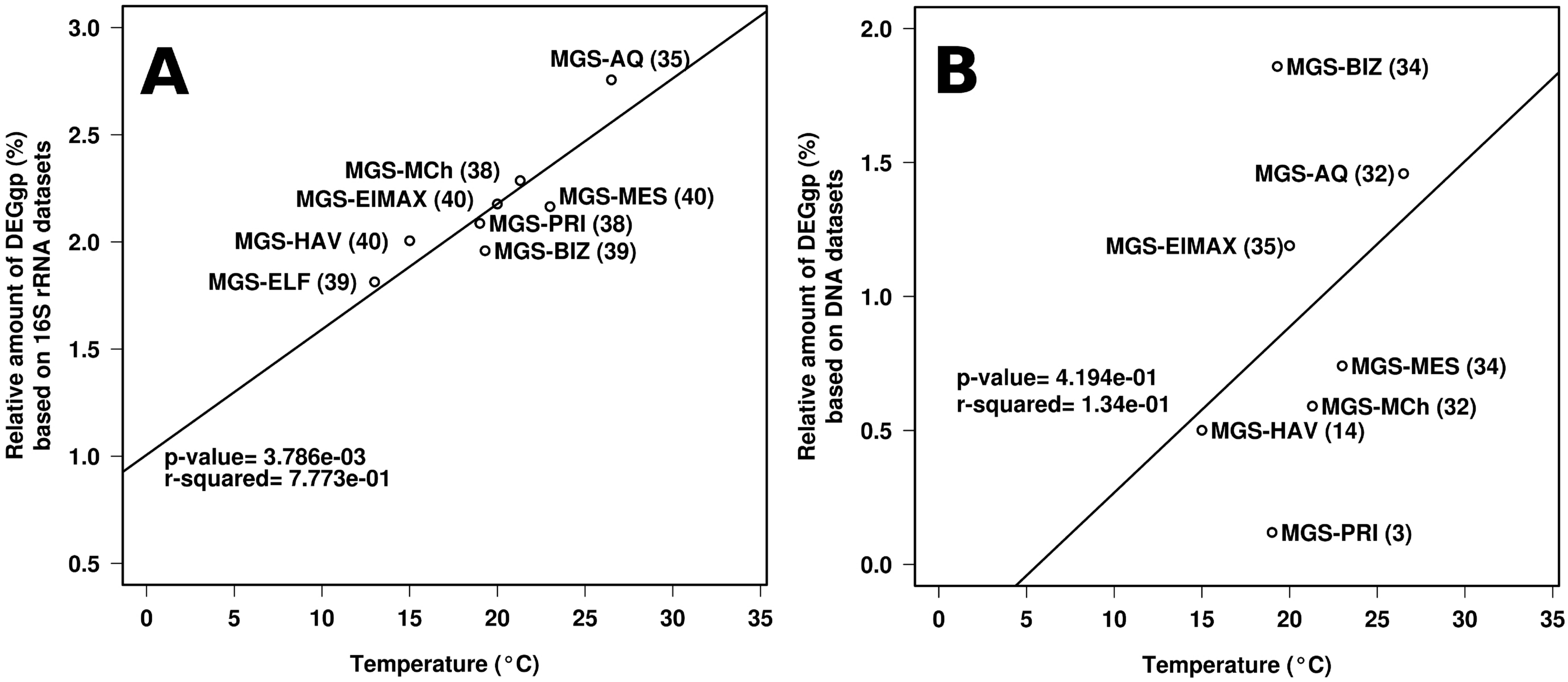
A
MGS-AQ (35)
MGS-MCh (38)
MGS-EIMAX (40)
MGS-MES (40)
MGS-PRI (38)
MGS-HAV (40)
MGS-BIZ (39)
MGS-ELF (39)
p-value= 3.786e-03
r-squared= 7.773e-01
Relative amount of DEGgp (%) based on 16S rRNA datasets
Temperature (°C)
B
MGS-BIZ (34)
MGS-AQ (32)
MGS-EIMAX (35)
MGS-MES (34)
MGS-MCh (32)
MGS-HAV (14)
MGS-PRI (3)
p-value= 4.194e-01
r-squared= 1.34e-01
Relative amount of DEGgp (%) based on DNA datasets
Temperature (°C)

Supplementary Fig. S6 Multi-panel map of the spatial distribution of the relative abundance level of genes encoding catabolic enzymes in the study area, referred to the total number of genes, as detected in the meta-sequences. Values are represented by colored dots. See legend in each panel as a reference. Spatial distributions of genes encoding catabolic enzyme percentages (on the basis of DNA + 16S rRNA data sets) in the study area were produced using Golden Software Surfer 8.0. The data are plotted as colored dots showing the true values at each sampling station. Note that, genomic evidence for the conversion of carbazole to anthranilate (via CarA and 2'-aminobiphenyl-2,3-diol 1,2-dioxygenase) was observed at all sites; however, further conversion to catechol (via AndA) was not detected in the MCh, ELMAX, ELF and AQ samples, suggesting that the complete mineralization of this pollutant most likely does not occur at those sites. Site temperatures are indicated in the panels. Reactions associated to genes encoding enzymes in panels, as follows:

Phenol hydroxylase (PhO): phenol ⇒ catechol

Carbazol dioxygenase (CarA): carbazol ⇒ 2'-aminobiphenyl-2,3-diol (code 009, Fig. 3)

Antranilate-1,2-dioxygenase (AndA): anthranilate (code 37, Fig.3) ⇒ catechol

Benzene dioxygenase (Ben): benzene ⇒ cis-1,2-dihydrobenzene-1,2-diol (code 048, Fig. 3)

Benzoate-1,2-dioxygenase: benzoate (code 039, Fig. 3) ⇒ catechol

2-Chlorobenzoate-1,2-dioxygenase: 2-chlorobenzoate (code 044, Fig. 3) ⇒ 2-chlorocatechol (code 084, Fig. 3)

Aromatic demethylase: 4-methyl-o-phthalate ⇒ protocatechuate

3-Nitrobenzoate-1,2-dioxygenase: 3-nitrobenzoate ⇒ *p*-hydroxyaminobenzoate (code 021, Fig. 3)

4-Hydroxybenzoate 3-monoxygenase: 4-hydroxybenzoate (code 028, Fig. 3) ⇒ protocatechuate

Toluene dioxygenase: toluene ⇒ toluene-cis-dihydrodiol (code 049, Fig. 3)

Benzyl succinate synthase: benzylsuccinate (code 041, Fig. 3) ⇒ benzoyl-CoA (code 047, Fig. 3).

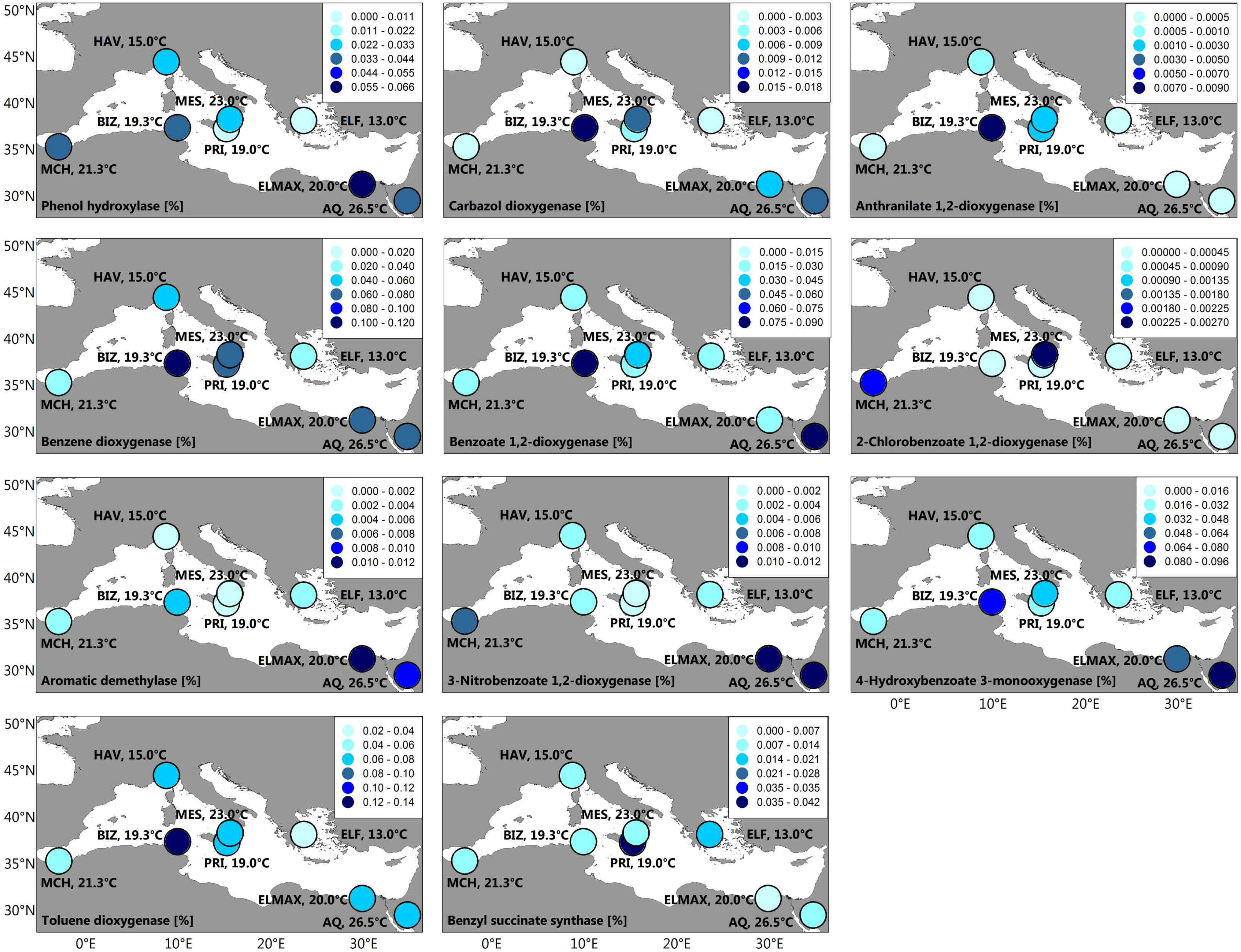

50°N
45°N
40°N
35°N
30°N
HAV, 15.0°C
MES, 23.0°C
BIZ, 19.3°C
ELF, 13.0°C
PRI, 19.0°C
MCH, 21.3°C
ELMAX, 20.0°C
AQ, 26.5°C
Phenol hydroxylase [%]
0.000 - 0.011
0.011 - 0.022
0.022 - 0.033
0.033 - 0.044
0.044 - 0.055
0.055 - 0.066
Carbazol dioxygenase [%]
0.000 - 0.003
0.003 - 0.006
0.006 - 0.009
0.009 - 0.012
0.012 - 0.015
0.015 - 0.018
Anthranilate 1,2-dioxygenase [%]
0.0000 - 0.0005
0.0005 - 0.0010
0.0010 - 0.0030
0.0030 - 0.0050
0.0050 - 0.0070
0.0070 - 0.0090
Benzene dioxygenase [%]
0.000 - 0.020
0.020 - 0.040
0.040 - 0.060
0.060 - 0.080
0.080 - 0.100
0.100 - 0.120
Benzoate 1,2-dioxygenase [%]
0.000 - 0.015
0.015 - 0.030
0.030 - 0.045
0.045 - 0.060
0.060 - 0.075
0.075 - 0.090
2-Chlorobenzoate 1,2-dioxygenase [%]
0.00000 - 0.00045
0.00045 - 0.00090
0.00090 - 0.00135
0.00135 - 0.00180
0.00180 - 0.00225
0.00225 - 0.00270
Aromatic demethylase [%]
0.000 - 0.002
0.002 - 0.004
0.004 - 0.006
0.006 - 0.008
0.008 - 0.010
0.010 - 0.012
3-Nitrobenzoate 1,2-dioxygenase [%]
0.000 - 0.002
0.002 - 0.004
0.004 - 0.006
0.006 - 0.008
0.008 - 0.010
0.010 - 0.012
4-Hydroxybenzoate 3-monooxygenase [%]
0.000 - 0.016
0.016 - 0.032
0.032 - 0.048
0.048 - 0.064
0.064 - 0.080
0.080 - 0.096
Toluene dioxygenase [%]
0.02 - 0.04
0.04 - 0.06
0.06 - 0.08
0.08 - 0.10
0.10 - 0.12
0.12 - 0.14
Benzyl succinate synthase [%]
0.000 - 0.007
0.007 - 0.014
0.014 - 0.021
0.021 - 0.028
0.035 - 0.035
0.035 - 0.042
0°E
10°E
20°E
30°E
